# Adjuvant chemoradiotherapy versus radiotherapy alone in women with high-risk endometrial cancer (PORTEC-3): 10-year clinical outcomes and post-hoc analysis by molecular classification from a randomised phase 3 trial

**DOI:** 10.1016/S1470-2045(25)00379-1

**Published:** 2025-10

**Authors:** Cathalijne C B Post, Stephanie M de Boer, Melanie E Powell, Linda Mileshkin, Dionyssios Katsaros, Paul Bessette, Alexandra Leary, Petronella B Ottevanger, Mary McCormack, Pearly Khaw, Romerai D'Amico, Anthony Fyles, Cyrus Chargari, Henry C Kitchener, Viet Do, Andrea Lissoni, Diane Provencher, Catherine Genestie, Hans W Nijman, Karen Whitmarsh, Ina M Jürgenliemk-Schulz, Amanda Feeney, Ludy C H W Lutgens, Jeanette Bouma, Alicia Leon-Castillo, Remi A Nout, Hein Putter, Tjalling Bosse, Carien L Creutzberg

**Affiliations:** a Department of Radiation Oncology, Leiden University Medical Centre, Leiden, Netherlands; b Department of Clinical Oncology, Barts Health NHS Trust, London, UK; c Department of Medical Oncology, Peter MacCallum Cancer Centre, Melbourne, VIC, Australia; d Department of Surgical Sciences and Gynaecology, Città della Salute and S Anna Hospital, Turin, Italy; e Department of Gynaecologic Oncology, University of Sherbrooke, Sherbrooke, QC, Canada; f Department of Cancer Medicine and Gynaecological Tumours Translational Research Lab, Gustave Roussy Cancer Centre, INSERM U981, Université Paris Saclay, Villejuif, France; g Department of Medical Oncology, Radboudumc, Nijmegen, Netherlands; h Department of Clinical Oncology, University College Hospital London, London, UK; i Department of Radiation Oncology, Peter MacCallum Cancer Centre, Melbourne, VIC, Australia; j Department of Radiation Oncology, Azienda Socio Sanitaria Territoriale, Lecco, Italy; k Department of Radiation Oncology, Princess Margaret Cancer Centre, Toronto, ON, Canada; l Department of Radiation Oncology, Gustave Roussy Cancer Campus, Paris, France; m Institute of Cancer Sciences, University of Manchester, Manchester, UK; n Liverpool Cancer Therapy Centre, Sydney, NSW, Australia; o Department of Obstetrics and Gynaecology, IRCCS San Gerardo dei Tintori, Monza, Italy; p Department of Gynaecologic Oncology, Centre hospitalier de l'Université de Montréal, Montréal, QC, Canada; q Department of Pathology, Gustave Roussy Cancer Centre, Villejuif, France; r Department of Gynaecologic Oncology, University Medical Centre Groningen, Groningen, Netherlands; s Department of Radiation Oncology, The Clatterbridge Cancer Centre NHS Trust, Liverpool, UK; t Department of Radiation Oncology, University Medical Centre Utrecht, Utrecht, Netherlands; u Cancer Research UK and UCL Cancer Trials Centre, UCL Cancer Institute, London, UK; v MAASTRO Clinic Radiation Oncology, Maastricht, Netherlands; w Central Data Management and Trial Coordination, Comprehensive Cancer Centre Netherlands, Rotterdam, Netherlands; x Department of Pathology, Leiden University Medical Centre, Leiden, Netherlands; y Department of Medical Statistics, Leiden University Medical Centre, Leiden, Netherlands

## Abstract

**Background:**

The PORTEC-3 trial investigated the benefit of chemoradiotherapy versus pelvic radiotherapy alone for women with high-risk endometrial cancer. We present the preplanned long-term analysis of the randomised PORTEC-3 trial with a post-hoc analysis including molecular classification of the tumours.

**Methods:**

PORTEC-3 was an open-label, multicentre, randomised, international phase 3 trial. Women were eligible if they had high-risk endometrial cancer (either International Federation of Gynecology and Obstetrics 2009 stage I, grade 3, with deep myometrial invasion and/or lymphovascular space invasion; stage II–III; or stage I–III with serous or clear-cell histology), were aged 18 years or older, and had a WHO performance score of 0–2. Participants were randomly assigned (1:1) to receive pelvic radiotherapy (48·6 Gy in 1·8 Gy fractions) or chemoradiotherapy (radiotherapy combined with two cycles of cisplatin 50 mg/m^2^ intravenously in weeks one and four, followed by four cycles of carboplatin area-under-the-curve 5 and paclitaxel 175 mg/m^2^ intravenously at 3-week intervals). Randomisation was done by use of biased-coin minimisation with stratification for participating centre, lymphadenectomy, stage, and histological type. We report the primary outcomes of overall survival and recurrence-free survival at 10 years. We also report primary outcomes by molecular subgroup in a post-hoc analysis. Survival was analysed in the intention-to-treat population. The study is registered with ClinicalTrials.gov (NCT00411138) and is now complete.

**Findings:**

Between Nov 23, 2006, and Dec 20, 2013, 660 eligible and evaluable patients recruited at 103 centres in six clinical trial groups across seven countries were randomly assigned to chemoradiotherapy (n=330) or radiotherapy alone (n=330). Median follow-up was 10·1 years (IQR 9·8–11·0). Estimated 10-year overall survival was 74·4% (95% CI 69·8–79·4) in the chemoradiotherapy group and 67·3% (62·3–72·7) in the radiotherapy group (adjusted hazard ratio [HR] 0·73 [95% CI 0·54–0·97], p=0·032), and 10-year recurrence-free survival was 72·8% (67·2–77·6) versus 67·4% (61·7–72·4; adjusted HR 0·74 [95% CI 0·56–0·98], p=0·034). Molecular analysis was available for 411 (62%) patients (210 [64%] of 330 patients in the chemoradiotherapy group and 201 [61%] of 330 patients in the radiotherapy group), whose characteristics were similar to the overall trial population. Post-hoc analysis by molecular class showed that, for women with p53 abnormal tumours, 10-year overall survival was 52·7% (95% CI 40·8–68·1) with chemoradiotherapy versus 36·6% (25·0 to 53·7) with radiotherapy alone (adjusted HR 0·52 [95% CI 0·30–0·91], p=0·021); 10-year recurrence-free survival was 52·6% (95% CI 38·3 to 65·0) versus 37·0% (95% CI 23·7 to 50·2; HR 0·42 [95% CI 0·24 to 0·74], p=0·0027). MMRd and POLEmut cancers did not seem to benefit from chemoradiotherapy over radiotherapy alone, whereas the effects for NSMP cancers were modulated by oestrogen-receptor status.

**Interpretation:**

10-year overall survival and recurrence-free survival were improved for patients with high-risk endometrial cancer treated with adjuvant chemoradiotherapy versus radiotherapy alone, with most clinically relevant benefit suggested for p53 abnormal cancers.

**Funding:**

Dutch Cancer Society, Cancer Research UK, National Health and Medical Research Council Australia, Cancer Australia, Italian Medicines Agency, and the Canadian Cancer Society Research Institute.

## Introduction

About 15–20% of patients with endometrial cancer present with high-risk disease and have an increased risk of recurrence and death. High-risk characteristics include early-stage disease with substantial lymphovascular space invasion, high-grade and/or non-endometrioid histology, and more advanced disease. To maximise locoregional control and recurrence-free interval, pelvic radiation therapy has been the standard adjuvant treatment for women with high-risk endometrial cancer. The randomised PORTEC-3 trial^1^, ^2^ compared adjuvant pelvic radiotherapy with chemotherapy during and after completion of radiotherapy versus pelvic radiotherapy alone for women with high-risk endometrial cancer, showing better overall survival and recurrence-free survival with chemoradiotherapy than with radiotherapy alone.

**Research in context**

**Evidence before this study**

In 2013, The Cancer Genome Atlas identified four distinct molecular subtypes of endometrial cancer based on genomic architecture. Subsequently, the TransPORTEC and ProMisE research groups showed that molecular classification could be assessed in clinical pathology with surrogate markers, yielding robust prognostic differences: tumours with a pathogenic *POLE* mutation (*POLE*mut) have an excellent prognosis, mismatch repair-deficient (MMRd) tumours have an intermediate prognosis, tumours with no specific molecular profile (NSMP) have a grade-dependent and stage-dependent intermediate prognosis, and p53-abnormal (p53abn) tumours have a poor prognosis. This molecular classification was incorporated into the 2020 WHO classification of endometrial cancer, and molecular subtype was integrated into risk-group assignment and treatment recommendations in the endometrial cancer guideline from the European Society for Medical Oncology and the joint guideline from the European Society of Gynecological Oncology, European Society for Radiotherapy and Oncology, and European Society of Pathology. We searched PubMed without language restrictions for studies published from database inception to Aug 28, 2024, with terms related to endometrial cancer, radiotherapy, chemotherapy, and survival. We aimed to identify studies with long-term results from randomised trials that included molecular classification of endometrial tumours and we found no randomised trials with follow-up of more than 5 years with integrated molecular classification.

**Added value of this study**

This 10-year analysis of the PORTEC-3 trial, with complete follow-up in 89% of the participants, supports previous evidence of significant improvements in both overall survival and recurrence-free survival with chemoradiotherapy versus radiotherapy alone for high-risk endometrial cancer. The greatest absolute benefit with chemoradiotherapy was seen in patients with p53abn cancers. Most recurrences occurred at distant sites, with patients in both treatment groups having excellent local and regional nodal control with pelvic radiotherapy. The prognostic and predictive value of the molecular classification and differences in added value of chemotherapy between molecular subgroups are clearly shown and have therapeutic implications.

**Implications of all the available evidence**

Adjuvant chemoradiotherapy provides improved recurrence-free and overall survival, with clinically relevant benefits occurring especially in p53abn endometrial cancers. For NSMP oestrogen receptor-negative tumours, chemoradiotherapy should be considered instead of either chemotherapy or radiotherapy alone. For high-risk NSMP ER-positive tumours, the benefit of chemotherapy might be smaller than previously hypothesised, and hormonal therapy could be a relevant alternative treatment option; further investigation is needed. Use of chemoradiotherapy does not seem to be of added value over radiotherapy alone in MMRd or *POLE*mut endometrial cancers. For high-risk MMRd endometrial cancer, adjuvant treatment with incorporation of immune checkpoint inhibition is being investigated.

Research has shown that the four molecular subgroups of endometrial cancer described by The Cancer Genome Atlas provide strong prognostic and predictive value.^3^, ^4^, ^5^, ^6^, ^7^ Accordingly, the WHO Classification of Tumours^8^ has integrated molecular parameters into its classification, and current endometrial cancer guidelines from the European Society of Gynecological Oncology, European Society for Radiotherapy and Oncology, European Society of Pathology,^9^ and European Society for Medical Oncology^10^ have incorporated molecular subtypes into risk assignment and treatment recommendations. Nonetheless, access to molecular testing is not uniform, and clinical uptake has been variable. Molecular classification is of particular importance in high-risk endometrial cancer, guiding treatment intensification or de-escalation. Within this seemingly unfavourable high-risk group, tumours with a pathogenic variant in the exonuclease domain of *POLE* (*POLE*mut) have an excellent prognosis, whereas tumours that are mismatch repair deficient (MMRd) or have no specific molecular profile (NSMP) have an intermediate prognosis, and tumours with abnormal (mutant-type) immunohistochemical expression of p53 (p53abn) have a poor prognosis.^7^

Molecular analysis in PORTEC-3^7^ and a large retrospective series from Canada^11^ showed improved outcomes in patients with p53abn endometrial cancer treated with platinum-based chemotherapy with radiotherapy versus radiotherapy alone, whereas no benefit of chemotherapy was observed for patients with high-risk MMRd endometrial cancer. Published international reports on patients with *POLE*mut endometrial cancers confirm the excellent prognosis of this subgroup, even when the tumours have unfavourable clinicopathological features.^7^, ^12^ The NSMP subgroup is heterogeneous, and multiple studies have shown that oestrogen receptor (ER) expression has a considerable prognostic impact in these patients, with most disease-specific deaths occurring in the small subset of patients with ER-negative cancers.^13^, ^14^, ^15^

The aims of this pre-planned long-term analysis of the PORTEC-3 trial were to evaluate the primary endpoints (overall and by histology and stage) and conduct a post-hoc analysis by molecular subgroup to inform treatment decisions and further research. In addition, we analysed patterns of recurrence and survival after recurrence.

## Methods

### Study design and participants

PORTEC-3^1^ was an open-label, multicentre, randomised, international phase 3 trial led by the Dutch Gynaecological Oncology Group. The trial was done at 103 centres by six clinical trial groups collaborating in the Gynaecological Cancer Intergroup. Participating groups were the UK National Cancer Research Institute, the Australia and New Zealand Gynaecologic Oncology Group, the Mario Negri Gynaecologic Oncology Group (Italy), the Canadian Cancer Trials Group, and Fedegyn (France). Participating groups, centres, and investigators are listed in the appendix (pp 1–2).

Details about patient selection and treatment have been published previously.^1^, ^2^, ^16^ Eligible patients had high-risk endometrial cancer (either endometrioid-type endometrial cancer, International Federation of Gynecology and Obstetrics [FIGO] 2009 stage I, grade 3, with deep myometrial invasion and/or any lymphovascular space invasion; stage II, IIIA, IIIC, or IIIB [parametrial invasion] endometrioid cancer; or stage I, II, or III with serous or clear-cell histology). Eligibility criteria included WHO performance score 0–2; adequate bone marrow, kidney, and liver function; and age 18 years or older. Exclusion criteria were uterine sarcoma or carcinosarcoma; previous malignancy (except non-melanoma skin cancer) within the past 10 years; previous pelvic radiotherapy, hormonal therapy, or chemotherapy; bulky cervical involvement; inflammatory bowel disease; residual macroscopic tumour; impaired renal or cardiac function; neuropathy grade 2 or higher; hearing impairment grade 3 or higher; or a congenital hearing disorder. Upfront central pathology review was done by reference gynaecopathologists for each group to confirm eligibility. Written informed consent was obtained from all patients. The protocol was approved by the Dutch Cancer Society and by the ethics committees of Leiden University Medical Centre, Leiden, Netherlands (P06.031) and all participating groups and is available in the appendix.

### Randomisation and masking

Patients were randomly allocated (1:1) to adjuvant chemoradiotherapy or radiotherapy alone by use of a web-based biased-coin minimisation procedure, ensuring balance overall and within each stratum of stratification factors:^17^, ^18^ participating group, type of surgery (lymphadenectomy [yes or no], and laparoscopic *vs* abdominal surgery), FIGO 2009 stage of cancer (IA *vs* IB *vs* II *vs* III) and histological type (endometrioid *vs* serous or clear-cell carcinoma). The assigned treatment was generated immediately by the randomisation system and confirmed by automated email. Participants, physicians, and investigators were not masked to treatment assignment.

### Procedures

All patients underwent abdominal or laparoscopic hysterectomy with bilateral salpingo-oophorectomy with or without lymphadenectomy. Clinically suspicious pelvic or periaortic lymph nodes were removed, but full lymphadenectomy was not mandatory, depending on the centre or group standard protocol. Patients in the radiotherapy alone group received pelvic radiotherapy to a dose of 48·6 Gy in 1·8 Gy fractions, five times per week, with a brachytherapy boost in case of cervical stromal involvement. Patients in the chemoradiotherapy group received the same radiotherapy combined with two concurrent cycles of cisplatin 50 mg/m^2^ intravenously in the first and fourth weeks of radiotherapy, followed by four adjuvant cycles of carboplatin area-under-the-curve 5 and paclitaxel 175 mg/m^2^ intravenously at 3-week intervals.

Follow-up was at regular intervals until 5 years. Long-term follow-up information was collected at 7 and 10 years from random assignment. After diagnosis of any relapse, treatment information was required, and follow-up was continued according to protocol guidelines.

Following consent from the participants, 423 formalin-fixed paraffin-embedded tissue samples were collected, of which 411 could be successfully molecularly classified. Molecular classification was done according to the algorithm provided by Vermij and colleagues^19^ and WHO^8^ and also described in other studies.^7^, ^9^ Briefly, endometrial cancers with a pathogenic *POLE* exonuclease domain mutation were classified as *POLE*mut (even if they were also p53abn and/or MMRd); endometrial cancers with loss of any mismatch repair protein or microsatellite instability (with or without a p53abn staining pattern) were classified as MMRd; cancers with only a p53abn staining pattern were classified as p53abn; and other endometrial cancers were classified as NSMP. ER staining was done on whole slides, and a 10% cutoff for positivity was used.

### Outcomes

The original coprimary endpoints were 5-year overall survival and failure-free survival. Here, the primary outcomes were 10-year overall survival and recurrence-free survival. Overall survival was defined as the time from random assignment to death from any cause. Recurrence-free survival was defined as time from random assignment to any relapse or as death related to endometrial cancer, whichever occurred first. Recurrence-free survival is presented in this analysis rather than failure-free survival as it is a more common outcome measure in adjuvant therapy trials in oncology.

The original secondary endpoints were vaginal, pelvic, or distant recurrence; treatment-related toxicity; and health-related quality of life. In this preplanned 10-year analysis, we evaluated long-term patterns of recurrences (vaginal, pelvic, or distant). Adverse events of grade 2 or worse severity were recorded at every follow-up visit, including those at 7 and 10 years, which could take place by telephone or via information from the general practitioner.

### Statistical analysis

The PORTEC-3 trial was powered (80%) to detect a 10% difference in 5-year overall survival between the treatment groups (increase from 65% to 75%, hazard ratio [HR] 0·67), with a two-sided test at an α level of 0·05. 198 overall survival events were required, with a minimum of 655 patients. These calculations were for the primary analysis with 5 years' follow-up.^1^, ^2^ In this preplanned 10-year analysis, all analyses were done in the intention-to-treat population. Median follow-up was estimated with the reverse Kaplan–Meier method. Women who were alive were followed until 10 years after random assignment. Although the final follow-up was planned at 10 years after the baseline, as outlined in the protocol, some patients continued to be followed up beyond this period, and in cases of death, information was provided. Thus, additional events were reported beyond the 10-year follow-up, but there was no elongation of follow-up without events. To maintain consistency in the analysis, all events occurring after 11 years of follow-up were censored in the statistical analyses, excluding nine death events (one in the radiotherapy group and eight in the chemoradiotherapy group, of which one was due to endometrial cancer, with follow-up time ranging from 11·0 years to 15·5 years).

Differences in relapse and survival between groups were assessed with the log-rank test and adjusted Cox regression analysis. Analysis of the primary endpoints was adjusted for the stratification factors. In the multivariable analysis, the following covariates were included together with treatment: stage, histological type and grade, type of surgery, participating group, lymphovascular space invasion, age, and molecular subgroup. We also conducted an updated post-hoc exploratory analysis of the primary outcomes by molecular subgroup, for the whole population. For the adjusted analysis in these subgroups, a simplified multivariable Cox model was used, with dichotomisation of the stratification factors (lymphadenectomy, stage, and histological type) to compensate for the low numbers of events in these subgroups. The proportional hazards assumption for treatment was checked by use of Schoenfeld residuals.

Competing risk methods were used for analyses of recurrence-free survival and vaginal, pelvic, and distant recurrence-free survival by calculating cumulative incidences and Fine-Gray regression. For the first recurrence analysis, all other recurrences and death were used as competing risks. For the total number of recurrences, intercurrent death alone was used as a competing risk. Recurrences were analysed according to first site of recurrence as well as by total number of recurrences for each type (vaginal, pelvic, or distant). Simultaneous vaginal and pelvic recurrence was considered pelvic recurrence, and simultaneous vaginal, pelvic, and distant recurrence was considered distant recurrence. Abdominal recurrences outside the pelvic area (peritoneal carcinomatosis, liver, and para-aortic lymph nodal metastases) were considered distant metastases, with specification of site. In addition, we conducted a post-hoc analysis of survival after recurrence, which was compared with a log-rank test, using the first site of recurrence.

Reported p values were based on two-sided tests, with p<0·05 considered statistically significant. Statistical analyses were done with R, version 4.1.0. This study is registered with ISRCTN (ISRCTN14387080) and ClinicalTrials.gov (NCT00411138).

### Role of the funding source

The funders of the study had no role in study design, data collection, data analysis, data interpretation, or writing of the report.

## Results

Between Nov 23, 2006, and Dec 20, 2013, 686 women were enrolled and randomly assigned to chemoradiotherapy (n=343) or radiotherapy alone (n=343); 26 patients were excluded after random assignment (13 due to immediate withdrawal of consent and 13 ineligible), resulting in 660 eligible and evaluable patients in the intention-to-treat analysis (330 in each group; appendix p 3).^1^, ^2^ Molecular classification of tumours was available for 411 (62%) of the 660 patients.^7^ The characteristics of these patients were similar to those of the rest of the trial population.^7^ Patient, treatment, and tumour characteristics are presented in table 1 and have been described in more detail previously.^1^, ^2^ No data on race or ethnicity were collected.

**Table 1:** Baseline characteristics by treatment group

		**Chemoradiotherapy (n=330)**	**Radiotherapy (n=330)**
**Age**			
Median (IQR), years		62·4 (56·5–67·9)	62·0 (55·8–68·2)
<60 years		128 (39%)	140 (42%)
60–69		144 (44%)	128 (39%)
≥70 years		58 (18%)	62 (19%)
**Stage**			
IA		39 (12%)	39 (12%)
IB		59 (18%)	58 (18%)
II		80 (24%)	90 (27%)
III		152 (46%)	143 (43%)
**Histology and grade**			
Endometroid grade 1–2		127 (39%)	131 (40%)
Endometroid grade 3		107 (32%)	106 (32%)
Serous		53 (16%)	52 (16%)
Clear cell		29 (9%)	33 (10%)
Other		14 (4%)	8 (2%)
**Lymphovascular space invasion (any)**			
Present		133 (40%)	192 (58%)
Absent		197 (60%)	138 (42%)
**Type of surgery**			
TAH and BSO		97 (29%)	97 (29%)
TAH and BSO plus LND or full staging		141 (43%)	132 (40%)
TLH and BSO		45 (14%)	42 (13%)
TLH and BSO plus LND or full staging		47 (14%)	59 (18%)
Molecular class*			
MMRd		67/210 (32%)	72/201 (36%)
*POLE*mut		23/210 (11%)	28/201 (14%)
P53abn		53/210 (25%)	46/201 (23%)
NSMP†		67/210 (32%)	55/201 (27%)
	NSMP ER-negative	6/210 (3%)	7/201 (4%)
	NSMP ER-positive	58/210 (28%)	46/201 (23%)

Data are n (%) or n/N (%) unless otherwise specified. Percentages might sum to more than 100% because of rounding. TAH=total abdominal hysterectomy. BSO=bilateral salpingo-oophorectomy. ER=oestrogen receptor. LND=lymph node dissection. MMRd=mismatch repair deficient. NSMP=no specific molecular profile. p53abn=p53 abnormal. *POLE*mut=*POLE* mutation. TLH=total laparoscopic hysterectomy.

* Molecular analysis was performed for 411 patients.

† Data on ER staining were missing or ambiguous for three patients in the chemoradiotherapy group and two patients in the radiotherapy group.

**Figure 1:**
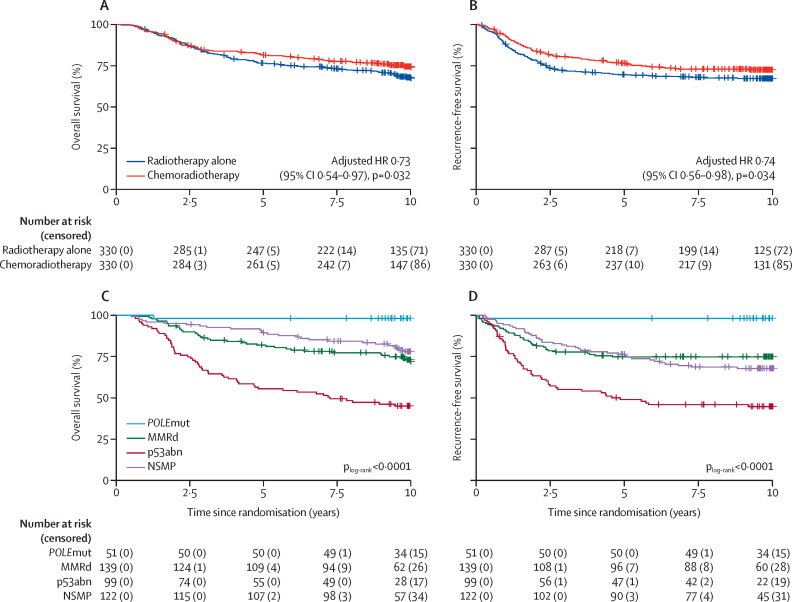
Kaplan–Meier survival curves for overall survival (A) and recurrence-free survival (B) in all patients by treatment group and for overall survival (C) and recurrence-free survival (D) by molecular group Analyses by molecular group (both treatment groups combined) were added post-hoc. HR=hazard ratio. MMRd=mismatch repair deficient. NSMP=no specific molecular profile. p53abn=p53 abnormal. *POLE*mut=*POLE* mutation.

At the final database lock (Dec 16, 2024), at least 9 years of follow-up information had been obtained for 89% of patients, and at least 7 years of follow-up were completed for 95% of patients. Median follow-up was 10·1 years (IQR 9·8–11·0). In total, 189 patients had died: 84 in the chemoradiotherapy group and 105 in the radiotherapy alone group. Most patient deaths were related to endometrial cancer: 65 (77%) of 84 deaths in the chemoradiotherapy group versus 85 (81%) of 105 in the radiotherapy alone group. The second most common cause of death was a secondary malignancy, which caused eight (10%) patient deaths in the chemoradiotherapy group versus 11 (10%) in the radiotherapy alone group. Estimated 10-year overall survival was 74·4% (95% CI 69·8–79·4) in the chemoradiotherapy group versus 67·3% (62·3–72·7) in the radiotherapy alone group (adjusted HR 0·73 [95% CI 0·54–0·97], p=0·032; figure 1). 199 patients developed disease recurrence (91 in the chemoradiotherapy group and 107 in the radiotherapy alone group). Estimated 10-year recurrence-free survival was 72·8% (67·2–77·6) following chemoradiotherapy versus 67·4% (61·7–72·4) with radiotherapy alone (adjusted HR 0·74 [95% CI 0·56–0·98], p=0·034; figure 1). Multivariable analyses for overall and recurrence-free survival are presented in table 2 and the appendix (p 4).

**Table 2:** Multivariable analysis of prognostic factors for overall survival and recurrence-free survival

	**Patients (n)**	**Overall survival**		**Recurrence-free survival**	
		HR (95% CI)*	p value	HR (95% CI)*	p value
**Treatment group**					
Radiotherapy	201	1 (ref)	..	..	..
Chemoradiotherapy	210	0·72 (0·50–1·04)	0·079	0·64 (0·45–0·91)	0·013
**Age**					
<60 years	178	1 (ref)	..	..	..
60–69 years	169	2·31 (1·43–3·74)	<0·0001	1·54 (1·01–2·35)	0·045
≥70 years	64	2·78 (1·54–5·00)	<0·0001	1·69 (0·99–2·90)	0·055
**Stage**					
I–II	233	1 (ref)	..	..	..
III	178	2·45 (1·64–3·65)	<0·0001	2·52 (1·71–3·72)	<0·0001
**Histology and grade**					
Endometroid grade 1–2	161	1 (ref)	..	..	..
Endometroid grade 3	132	1·44 (0·84–2·46)	0·19	1·05 (0·63–1·76)	0·84
Serous or clear cell	118	1·10 (0·60–2·02)	0·76	0·95 (0·53–1·71)	0·87
**Lymphovascular space invasion (any)**					
Absent	155	1 (ref)	..	..	..
Present	256	1·47 (1·01–2·36)	0·071	1·55 (1·05–2·30)	0·029
**Molecular class**					
MMRd	139	1 (ref)			
*POLE*mut	51	0·070 (0·010–0·54)	0·0090	0·072 (0·01–0·53)	0·0090
P53abn	99	2·29 (1·46–3·99)	<0·0001	2·83 (1·71–4·68)	<0·0001
NSMP	122	0·69 (0·39–1·16)	0·16	1·12 (0·70–1·80)	0·63

Analyses by molecular group were added post-hoc. HR=hazard ratio. MMRd=mismatch repair-deficient. NSMP=no specific molecular profile. p53abn=p53 abnormal. *POLE*mut=*POLE* mutation.

* Adjusted for the following stratification factors: participating group and lymphadenectomy.

Table 3 shows 5-year and 10-year probabilities of recurrence by treatment group. Patterns of first recurrence at 5 and 10 years did not differ significantly between the treatment groups, although there were more distant recurrences in the radiotherapy alone group than in the chemoradiotherapy group. In both groups, most recurrences occurred in the first 2·5 years after treatment (61 [67%] of 91 after chemoradiotherapy and 87 [81%] of 107 after radiotherapy alone). In the chemoradiotherapy group, 14 (15%) of 91 recurrences 0occurred after 5 years of follow-up (one vaginal and 13 distant). In the radiotherapy group, eight (7%) of 107 recurrences occurred after 5 years of follow-up (two pelvic and six distant). Patients who had a late recurrence (ie, after 5 years of follow-up) had low-grade rather than high-grade endometrial cancer more often than did patients who had a recurrence in the first 5 years of follow-up (15 [68%] of 22 *vs* 53 [30%] of 176; p=0·0010) and more tumours of the NSMP molecular subgroup (11 [64·7%] of 17 *vs* 29 [25·4%] of 114, p=0·011; all tumours were ER-positive); there were no significant differences between treatment groups (data not shown).

**Table 3:** Recurrence outcomes by treatment group for all study patients

	**Number of events**	**5-year probability (95% CI)**	**10-year probability (95% CI)**	**Hazard ratio (95% CI)**	Log-rank p value*
**Vaginal (first)**					
Chemoradiotherapy	2	0·3% (0·04–2·1)	0·6% (0·2–2·5)	2·00 (0·18–22·02)	0·57
Radiotherapy	1	0·3% (0·04–2·1)	0·3% (0·04–2·1)	1 (ref)	..
**Pelvic (first)**					
Chemoradiotherapy	3	0·9% (0·3–2·8)	0·9% (0·3–2·8)	0·60 (0·14–2·50)	0·48
Radiotherapy	5	0·9% (0·3–2·8)	1·6% (0·7–3·7)	1 (ref)	..
**Distant (first)**					
Chemoradiotherapy	86	22·2% (18·1–27·1)	25·7% (21·3–30·8)	0·80 (0·60–1·07)	0·14
Radiotherapy	101	28·9% (24·3–34·1)	30·5% (25·8–35·8)	1 (ref)	..
**Vaginal (total)**					
Chemoradiotherapy	9	2·1% (1·0–4·4)	2·8% (1·5–5·2)	1·12 (0·43–2·90)	0·81
Radiotherapy	8	2·1% (1·0–4·4)	2·1% (1·02–4·41)	1 (ref)	..
**Pelvic (total)**					
Chemoradiotherapy	23	6·4% (4·2–9·6)	7·0% (4·7–10·4)	0·61 (0·36–1·02)	0·060
Radiotherapy	37	10·0% (7·2–13·8)	11·3% (8·3–15·3)	1 (ref)	..
**Distant (total)**					
Chemoradiotherapy	86	22·8% (18·6–27·7)	26·3% (21·9–31·4)	0·81% (0·61–1·08)	0·16
Radiotherapy	101	29·2% (24·6–34·4)	30·8% (26·1–36·1)	1 (ref)	..

* Hazard ratio using Fine-Gray regression was unadjusted for stratification factors.

**Figure 2:**
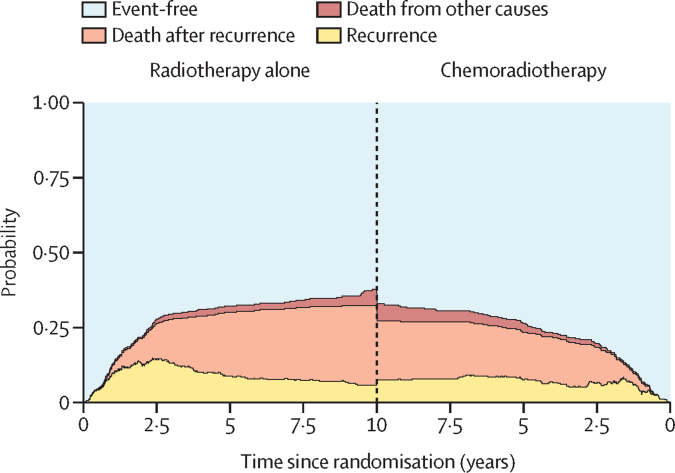
Events and survival by treatment group for all patients Seven patients died due to other causes after recurrence (four after radiotherapy alone and three after chemoradiotherapy); these patients are included in the death after recurrence group.

Median overall survival after recurrence was 1·4 years (IQR 0·4–4·3): 1·2 years (0·4–8·8) after chemoradiotherapy and 1·4 years (0·7–3·9) after radiotherapy alone (p=0·90). 5-year overall survival after recurrence was 27·7% (95% CI 19·6–39·1) after chemoradiotherapy versus 21·5% (14·0–30·0) after radiotherapy alone. Four patients died within 5 years following recurrence due to intercurrent disease (figure 2). Factors associated with better prognosis after recurrence were younger age, low-grade endometrioid histology, ER-positive NSMP tumours, and para-aortic lymph node or single-organ lung metastases (data not shown). p53abn tumours and multiple distant sites of recurrence were poor prognostic factors (data not shown). Proportions of therapy types received for a first recurrence by treatment group and data on 5-year adverse events as reported previously^2^ are provided in the appendix (pp 5–6).

Further preplanned analyses showed that for women with stage III disease (n=295), 10-year overall survival was 69·5% (95% CI 62·4–77·4) with chemoradiotherapy versus 56·1% (48·3–65·3) with radiotherapy alone (adjusted HR 0·66 [95% CI 0·45–0·97], p=0·033), and 10-year recurrence-free survival was 67·0% (58·3–74·2) versus 55·5% (46·5–63·5; adjusted HR 0·65 [95% CI 0·45–0·93], p=0·020; appendix p 7). For women with serous cancers of stages I–III (n=105), 10-year overall survival was 57·1% (45·0–72·5) with chemoradiotherapy versus 41·8% (30·3–57·8) with radiotherapy alone (adjusted HR 0·55 [95% CI 0·31–0·98], p=0·044), and 10-year recurrence-free survival was 56·4% (41·9–68·5) versus 46·0% (32·5–58·6; adjusted HR 0·47 [95% CI 0·26–0·86], p=0·013; appendix pp 7–8). Overall survival and recurrence-free survival in patients with combinations of p53abn versus p53-wildtype and serous versus non-serous endometrial cancers are shown in the appendix (p 8). 48 (74%) of 65 serous cancers were p53abn, whereas 48 (48%) of 99 p53abn endometrial cancers had serous histology.

In the post-hoc analyses by molecular class (n=411 patients in the total population; n=210 in the chemoradiotherapy group and n=201 in the radiotherapy group), 10-year overall survival was 45·1% (95% CI 36·2–56·1) for patients in the p53abn subgroup, 98·0% (94·3–100·0) for those in the *POLE*mut subgroup, 71·7% (64·2–80·0) for those in the MMRd subgroup, and 77·9% (70·7–85·8) for those in the NSMP subgroup (p_log-rank_<0·0001; figure 1). 10-year recurrence-free survival was 45·3% (95% CI 35·5–54·7) for patients in the p53abn subgroup, 98·0% (86·9–99·7) for those in the *POLE*mut subgroup, 74·7% (67·7–81·7) for those in the MMRd subgroup, and 67·8% (58·1–75·8) for those in the NSMP subgroup (p_log-rank_<0·0001; figure 1).

**Figure 3:**
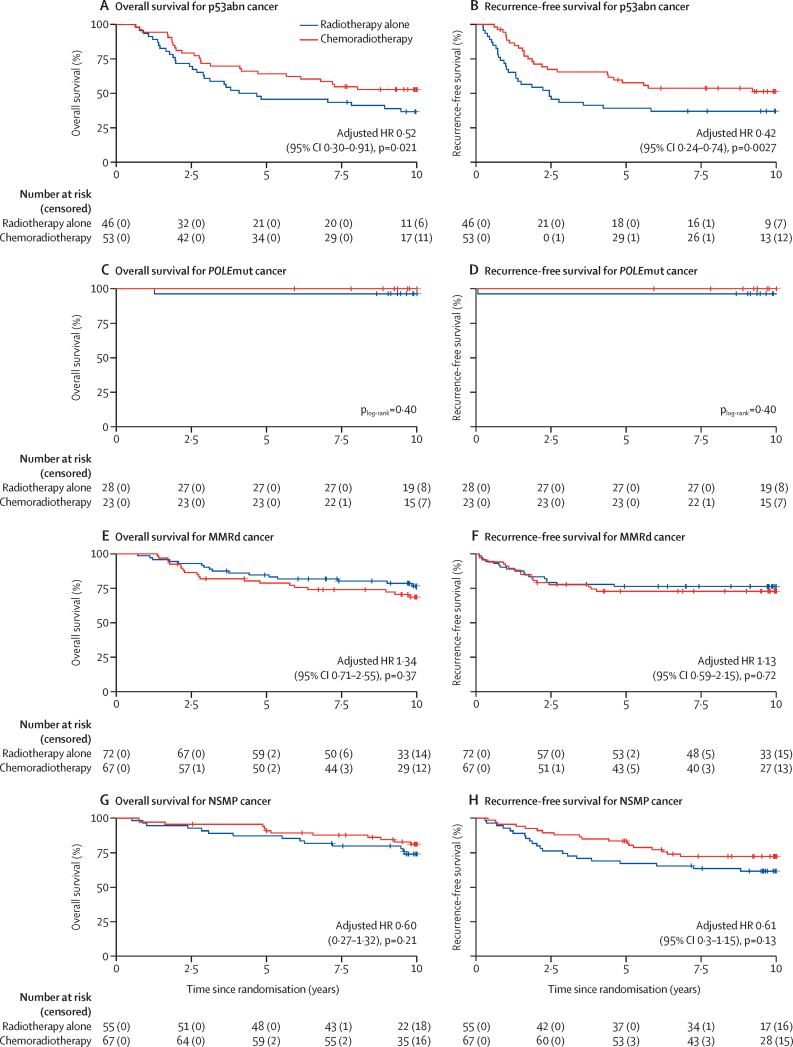
Kaplan–Meier survival curves for overall survival and failure-free survival among patients with p53abn (A–B), *POLE*mut (C–D), MMRd (E–F), and NSMP (G–H) tumours by treatment group MMRd=mismatch repair deficient. NSMP=no specific molecular profile. p53abn=p53 abnormal. *POLE*mut=*POLE* mutation.

10-year overall survival with chemoradiotherapy versus radiotherapy alone per molecular subgroup was 52·7% (95% CI 40·8–68·1) versus 36·6% (25·0–53·7) for p53abn cancers (adjusted HR 0·52 [95% CI 0·30–0·91], p=0·021); 100·0% (100·0–100·0) versus 96·4% (89·8–100·0) for *POLE*mut cancers (p_log-rank_=0·40); 68·7% (58·1–81·2) versus 74·4% (64·4–86·0) for MMRd cancers (adjusted HR 1·34 [95% CI 0·71–2·55], p=0·37); and 81·2% (72·1–91·4) versus 74·1% (63·2–86·8) for NSMP cancers (adjusted HR 0·60 [0·27–1·32], p=0·21; figure 3). 10-year recurrence-free survival with chemoradiotherapy versus radiotherapy was 52·6% (95% CI 38·3–65·0) versus 37·0% (23·7–50·2) for p53abn cancers (adjusted HR 0·42 [95% CI 0·24–0·74], p=0·0027); 100·0% (100·0–100·0) versus 96·4% (77·2–99·5) for *POLE*mut cancers (p_log-rank_=0·40); 72·9% (59·1–82·7) versus 76·4% (63·4–85·3) for MMRd cancers (adjusted HR 1·13 [95% CI 0·59–2·15], p=0·72); and 72·8% (59·0–82·6) versus 61·7% (47·0–73·4) for NSMP cancers (adjusted HR 0·61 [95% CI 0·33–1·15], p=0·13; figure 3).

There was no evidence of a difference in treatment effect size across disease stages among patients with p53abn cancers (appendix pp 9–10). In our exploratory analysis of survival for patients with NSMP endometrial cancer by ER status, 10-year overall survival for patients with ER-positive NSMP tumours of all stages was 81·8% (95% CI 72·2–92·7%) for patients receiving chemoradiotherapy versus 82·3% (71·8 to 94·2) for patients receiving radiotherapy alone (p_log-rank_=1·00). For patients with stage 3 ER-positive NSMP tumours, 10-year overall survival was 78·9% (66·2–94·1) for patients receiving chemoradiotherapy versus 83·8% (70·5–99·7) for patients receiving radiotherapy (p_log-rank_=0·70; appendix pp 11–12). Estimated 10-year recurrence-free survival for all patients with ER-positive NSMP tumours was 75·6% (95% CI 60·4–85·6) for patients receiving chemoradiotherapy versus 67·3% (51·1–9·2) for patients receiving radiotherapy alone (p_log-rank_=0·40), and, for patients with stage III ER-positive NSMP disease, it was 74·3% (53·7–86·7) for patients receiving chemoradiotherapy versus 60·0% (38·0–76·4) for patients receiving radiotherapy alone (p_log-rank_=0·30; appendix p 11). For patients with ER-negative NSMP tumours, all recurrence events (two of six patients treated with chemoradiotherapy and five of seven patients treated with radiotherapy alone) occurred within 2·5 years after treatment (appendix p 11). One of these patients, who was treated with chemoradiotherapy and had a solitary para-oesophageal lymph node metastasis after 2·2 years had a successful salvage resection; all other patients with recurrent disease died due to endometrial cancer. Analysis of grade 3 NSMP cancers showed higher 10-year recurrence-free survival with chemoradiotherapy than with radiotherapy alone, but these findings were not significant (appendix pp 11–12). Outcomes per treatment group, separated by stage, for patients with MMRd endometrial cancer are in the appendix (p 12), showing no benefit of chemoradiotherapy over radiotherapy alone for MMRd cancers.

## Discussion

This long-term analysis of the PORTEC-3 trial with 10-year outcomes supports previous findings^1^, ^2^, ^20^ of a significant improvement in both overall and recurrence-free survival with chemoradiotherapy compared with radiotherapy alone for patients with high-risk endometrial cancer. Most recurrences occurred at distant sites, with excellent local and regional nodal control in both treatment groups. In this 10-year analysis, the prognostic and predictive value of the molecular classification and the differences between the four molecular groups in terms of the added value of chemotherapy are highlighted as they have important therapeutic implications.

To our knowledge, this is the first study with a 10-year follow-up of a randomised trial for high-risk endometrial cancer. The 10-year overall and recurrence-free survival deviate minimally from the previous published 5-year results.^2^, ^7^ The differences between overall survival and recurrence-free survival benefits at 10 years and those at 5 years might be explained by the higher frequency of late recurrences in the chemoradiotherapy group, with more delayed recurrences than in the radiotherapy group. These late recurrences generally had more favourable characteristics, with longer survival after treatment for recurrence compared with recurrences within 5 years of follow-up. Therefore, it was plausible that the 10-year overall survival benefit would not decrease, due to these late recurrences, but an increase in overall survival benefit was not anticipated. Nonetheless, such an increase in overall survival benefit appeared between 8 years and 10 years of follow-up due to more deaths occurring in the radiotherapy group than in the chemoradiotherapy group during this period. However, as most of the events were unrelated to endometrial cancer in both treatment groups and the number of events was low, this observation is likely attributable to random effects.

The excellent vaginal and pelvic nodal control achieved with external beam radiotherapy was still evident in both groups in this long-term follow-up. This finding is similar to results obtained in both the GOG-258^21^ and GOG-249^22^ trials, which showed significantly improved pelvic and para-aortic nodal control in the treatment groups that received pelvic radiotherapy rather than chemotherapy alone (GOG-258) or chemotherapy with vaginal brachytherapy (GOG-249).

In line with the previous PORTEC-3 analyses,^7^, ^13^ patients with stage III disease and those with stage I–III p53abn (and/or serous) endometrial cancers had the greatest benefit from chemoradiotherapy, as shown by the HRs for both overall survival and recurrence-free survival. Among patients with stage III disease, the treatment effect was primarily driven by p53abn cancers and a subset of NSMP cancers. There were few late recurrences after 5 years of follow-up, and overall survival benefit in the chemoradiotherapy group seemed to increase slightly over time, probably due to more endometrial cancer-related deaths with longer follow-up.

In our post-hoc analysis by molecular group, p53abn cancers had a poor prognosis overall and a benefit from chemoradiotherapy. Subgroup sample sizes were too small to test for treatment effect modification by subgroup. Serous histology and p53abn molecular class were correlated, but they were not interchangeable: 74% of the serous cancers were p53abn and 48% of the p53abn cancers had serous histology. These percentages are similar to those found in a previous study on *TP53*-mutated endometrial cancers.^23^ Treatment recommendations based solely on histology could lead to undertreatment of a substantial proportion of patients with p53abn cancers and, potentially, to overtreatment of serous carcinomas that are not p53abn and have a relatively favourable prognosis—highlighting that molecular classification is essential for treatment recommendations. Moreover, it can be difficult to distinguish serous cancer from other histological types. In this trial, central expert gynaecopathology review was done, resulting in more precise histological classification than in clinical practice. For p53abn endometrial cancers, the overall survival and recurrence-free survival benefit with chemoradiotherapy remained stable after 5 years, suggesting cure for some patients and delay of recurrence and death in others.

In the previous molecular analysis of PORTEC-3,^7^ a trend for improved survival with chemoradiotherapy was reported for NSMP tumours. In this long-term analysis, the differences in recurrence-free survival decreased over time, implying that, for some patients with NSMP tumours, recurrences are delayed. The NSMP subgroup is heterogenous, with multiple studies showing that ER status has a strong prognostic value.^13^, ^14^, ^15^ The exploratory analysis stratified for ER status supported the relatively favourable prognosis of ER-positive NSMP tumours and showed postponement of recurrence but no overall survival benefit. In contrast, a small proportion of high-risk NSMP tumours (∼10%) are ER-negative and have a poor prognosis. Although the numbers in this analysis were very small, meaning that data should be interpreted with caution, patients with ER-negative NSMP tumours seemed to benefit from chemotherapy, with substantial effect sizes for recurrence-free survival and overall survival.

Most recurrences at distant sites occurred within the first 3 years.^2^ A proportion of patients with distant metastases, especially younger patients with low-grade endometrioid and ER-positive NSMP cancers, had successful salvage therapy. Late recurrences were rare, with only 11% occurring after 5 years of follow-up, and were more frequent in the chemoradiotherapy group. These were most often ER-positive, low-grade, NSMP cancers, supporting long survival after recurrence, as reported previously.^2^ As late recurrences are uncommon and most are symptomatic, with patients presenting between follow-up visits, extended follow-up is not recommended. This approach is supported by the TOTEM study, which showed no improvement of overall survival with an intensified follow-up regimen, even in patients with high-risk endometrial cancer.^24^

A limitation of our study was that, even with long-term follow-up, the predefined threshold of 198 overall survival events was not fully reached, with 189 deaths in the analysis. Longer follow-up resulted in more intercurrent deaths. Although this analysis was a predefined translational research analysis, power calculations were not done for the specific molecular subgroup analyses, and the sample sizes were too small to enable interaction testing. However, the characteristics of the 62% of cases with molecular characterisation were similar to those of the overall trial population.^7^ In the subset analysis of treatment effects by molecular group, the treatment effect did not reach statistical significance, likely due to the smaller sample size. However, when molecular classification was incorporated into the multivariable model, the treatment effect became more pronounced, indicating a stronger association, despite the low statistical power. Another limitation might be that the definition of high-risk endometrial cancer has changed over time with extended knowledge.^9^ However, the subgroups with the largest chemotherapy benefit (stage I–III p53abn and serous cancers and stage III cancers) are still classified as high risk. Our results, therefore, support current European Society of Gynaecological Oncology, European Society for Radiotherapy and Oncology, and European Society of Pathology guidelines^9^ recommending adjuvant chemoradiotherapy for high-risk endometrial cancer.

Molecular classification has a crucial role in current diagnosis, prognostication, and decision making regarding adjuvant therapy, particularly for patients with high-risk endometrial cancer, for whom intensive treatments are involved. Treatment effect should be balanced against toxicities because acute severe adverse events and impaired health-related quality of life occur more frequently during treatment with chemotherapy compared with radiotherapy alone (45% *vs* 12%),^16^ as do late grade 2 adverse events (29% *vs* 19%), including persistent sensory neuropathy (6% *vs* 0%).^20^ Given the clinically relevant therapeutic benefit with chemotherapy for stage I–III p53abn cancers, adjuvant chemoradiotherapy is recommended. For other molecular subgroups, treatment effect is less pronounced and might not outweigh the added toxicity.

Our results emphasise the value of identifying the p53abn status of tumours compared with evaluating serous histology alone. Patients with p53abn cancers had the poorest outcomes, highlighting the need for new treatments. Treatment intensification with PARP inhibition is being investigated in the RAINBO-P53-Red trial.^25^ Previous analysis of PORTEC-3 showed that about 25% of p53abn cases were HER2-positive and 96% of HER2-positive cases were p53abn. Although HER2 status did not show an independent prognostic value for survival,^26^ for these HER2-positive cancers, as well as those with low HER2 expression, antibody–drug conjugates targeting HER2 are a promising therapeutic strategy, and trials are ongoing.^27^

All stage III NSMP and MMRd endometrial cancers are classified as high-risk disease, and chemoradiotherapy is recommended.^9^ However, patients with stage III MMRd tumours are less likely to benefit from the addition of chemotherapy to radiotherapy than are patients with stage III NSMP, as supported by the overlapping survival curves and shown in the previous molecular analysis of PORTEC-3 and a large retrospective series from Canada.^7^, ^11^ For these patients, immunotherapy is potentially a better option than chemotherapy, offering greater expected benefits and fewer side-effects. Multiple studies in the metastatic setting have shown promising results for immunotherapy in MMRd endometrial cancer.^28^, ^29^ A beneficial effect of immunotherapy was also found in the adjuvant setting in the subset of patients with stage III MMRd endometrial cancer in the recent Keynote-B21 trial.^30^, ^31^ However, in this trial, immunotherapy was combined with chemotherapy, and more than 50% of participants with stage III MMRd and more than 80% of those with stage I–II MMRd with non-endometrioid histology also had radiotherapy. The additive value of chemotherapy when immune checkpoint inhibition is used needs further study. Ongoing trials that are investigating adjuvant immunotherapy alone versus with radiotherapy or chemotherapy are the RAINBO-MMRd-Green (NCT05255653), DOMENICA (NCT05201547), and NRG-GY020 (NCT04214067) trials.

Patients with high-risk NSMP ER-negative tumours might benefit from the addition of chemotherapy to adjuvant radiotherapy. However, the proportion of ER-negative cases among NSMP cancers is small. Patients with stage III NSMP ER-positive tumours might benefit less from chemotherapy than previously anticipated—chemotherapy likely postpones or reduces recurrence events, but no overall survival benefit was observed. Additional adjuvant hormonal therapy could represent a more promising treatment approach for these patients, an option currently being investigated in the RAINBO-NSMP-Orange trial (NCT05255653), which is underway in the UK. Finally, irrespective of stage and based on the excellent recurrence-free survival, de-escalation should be recommended for *POLE*mut endometrial cancers, preferably in a study setting (such as the recently completed PORTEC-4a trial [ISRCTN11659025, NCT03469674] and the EN10.A/RAINBO-BLUE trial [NCT05640999]).

In conclusion, PORTEC-3 was a multicentre trial conducted through strong international collaboration, making it highly representative of current practice. This long-term analysis shows improved 10-year overall survival and recurrence-free survival for patients with high-risk endometrial cancer treated with chemoradiotherapy versus radiotherapy alone, with most clinically relevant benefit from chemoradiotherapy suggested for p53abn cancers.

### Contributors

### Data sharing

De-identified participant data will be made available on request to the corresponding author, senior author, or one of the international members of the TransPORTEC-consortium when analyses of all primary and secondary endpoints, including the translational research studies related to the trial, have been published, following the evaluation of a specific research proposal and completion of a data transfer agreement.

## Declaration of interests

SMdB reports grants from Varian and payment for lectures from the European School of Oncology, paid to their institution. CC declares payment or honoraria for educational events by MSD, GSK, Eisai, and Merck and institutional support for research from Roche and TherAguix. HWN declares grants from the Dutch Cancer Society. RAN declares research grants from Elekta, Varian, Accuray, Sensius, and Senewald and payment for lectures from Elekta, GSK, and MSD paid to their institution. DP reports consulting fees from AstraZeneca, GSK, Eisai, Merck, and AbbVie. ALC reports payments for lectures from Diaceutics and AstraZeneca. AL reports grants for research projects from AstraZeneca, Zentalis, and Owkin; consulting fees from Owkin, AstraZeneca, MSD, GSK, AbbVie, Immunogen, Pharm&, Genmab, Zentalis, and Daiichi Sankyo; and payment for educational events from GSK, AbbVie, AstraZeneca, and Ose Immunotherapeutics. CLC reports grants from the Dutch Cancer Society during the conduct of the study. All other authors declare no competing interests.
