# Supplementary material for: Adjuvant chemoradiotherapy versus radiotherapy alone in women with high-risk endometrial cancer (PORTEC-3): 10-year clinical outcomes and post-hoc analysis by molecular classification from a randomised phase 3 trial
Source: Lancet Oncol. 2025 Oct;26(10):1370–81. doi: 10.1016/S1470-2045(25)00379-1 (PMC12479390; doi:10.1016/S1470-2045(25)00379-1)
Supplement: Supplementary appendix [file mmc1.pdf]

THE LANCET
Oncology

Supplementary appendix

This appendix formed part of the original submission and has been peer reviewed. We post it as supplied by the authors.

Supplement to: Post CCB, de Boer SM, Powell ME, et al. Adjuvant chemoradiotherapy versus radiotherapy alone in women with high-risk endometrial cancer (PORTEC-3): 10-year clinical outcomes and post-hoc analysis by molecular classification from a randomised phase 3 trial. *Lancet Oncol* 2025; published online Sept 5. https://doi.org/10.1016/S1470-2045(25)00379-1.

Appendix

Supplementary data belonging to:
Adjuvant chemoradiotherapy versus radiotherapy alone in women with high-risk endometrial cancer (PORTEC-3): 10-year clinical outcomes and post-hoc analysis by molecular classification from a randomised phase 3 trial.

Contents

PORTEC 3 Study group and participating centres page 1

CONSORT diagram of the PORTEC-3 trial page 3

Table 1a. Multivariable analysis of prognostic factors for overall survival page 4

Table 1b. Multivariable analysis of prognostic factors for recurrence-free survival page 4

Table 2. Five-year adverse events reported in the PORTEC-3 trial page 5

Figure 1. Treatment for first recurrence by treatment arm page 6

Figure 2. Overall and recurrence-free survival in serous endometrial cancer by arm page 7

Figure 3. Overall and recurrence-free survival for p53abn vs p53wt; serous vs non-serous EC page 8

Figure 4. Overall and recurrence-free survival for p53-abnormal endometrial cancer page 9

Figure 5. Overall and recurrence-free survival in patients with NSMP tumours page 11

Figure 6. Overall and recurrence-free survival in patients with MMRd endometrial cancer page 13

PORTEC-3 clinical trial protocol (protocol pages 1-77) page 14

PORTEC 3 Study group and participating centres

PORTEC-3 Independent data monitoring committee: L.V.A.M. Beex, N. James, M.J.M. Olofsen-van Acht, W. Parulekar, W.L.J. van Putten, D. Rischin, J. Yarnold (chair)

PORTEC-3 trial statistician: H. Putter

List of participating countries and centres (listed in order of patients recruited):

United Kingdom: 184 patients (177 evaluable)
Study Coordinators: M. Powell (P.I.), London; H. Kitchener, Manchester; J. Ledermann, London
Group coordinating trial centre: Cancer Research UK and UCL Cancer Trials Centre, London
Trial pathologists: N. Singh, London; G. Wilson, Manchester
Participating centres and principal investigator(s) (number of patients): London-Barts Health NHS Trust (M. Powell, 23); London-University College London Hospitals NHS Foundation Trust (M. McCormack, 15); Bebington, Wirral-The Clatterbridge Cancer Centre NHS Foundation Trust (K. Whitmarsh, K. Hyatt, 12); Wolverhampton-The Royal Wolverhampton NHS Trust (R. Allerton, 11); Cambridge-Cambridge University Hospitals NHS Foundation Trust (L.T. Tan, M. Iddawela, D. Gregory, S. Ayres, 11); Leicester-Leicester Royal Infirmary (P. Symonds, 10); Northwood-Mount Vernon Cancer Centre (P. Hoskin, 10); Middlesborough-The James Cook University Hospital (A. Rathmell, M. Adusumalli, 9); Nottingham-Nottingham City Hospital (S. Chan, A Anand, 9); Norwich-Norfolk and Norwich University Hospitals NHS Foundation Trust (R. Wade, 8); Guildford-Royal Surrey County Hospital (A. Stewart, 6); Newcastle Upon Tyne-Freeman Hospital (W. Taylor, 6); Brighton-Brighton & Sussex University Hospitals NHS Trust (K. Lankester, 5); Coventry-University Hospital Coventry (C. Irwin, M. Hocking, 5); Taunton Musgrove Park Hospital (P. Jankowska, D. Milliken, C. Barlow, 5); Cheltenham-Cheltenham General Hospital (A. Cook, R. Counsell, 4); Exeter-Royal Devon & Exeter Hospital (P. Bliss, A. Hong, 4); Lincoln-Lincoln County Hospital (M. Panades, 4); Romford-Queens Hospital (M. Quigley, 4); Manchester-The Christie NHS Foundtion Trust (S. Davidson, 3); Northampton-Northampton General Hospital NHS Trust (C. Mak, 3); Preston-Royal Preston Hospital (A. Hindley, 3); Truro-Royal Cornwall Hospitals NHS Trust (A. Thomson, 3); Sheffield-Weston Park Hospital (S. Pledge, J. Martin, 2); Shrewsbury-Royal Shrewsbury Hospital (S. Awwad, A. Zachariah, 2); Carlisle-North Cumbria University Hospitals NHS Trust (S. Singhal, 1); Colchester-Essex County Hospital (A. Lamont, 1); London-Guy's and St. Thomas' NHS Foundation Trust (A. Winship, A. Montes, V. Mullassery, 1); London-Hammersmith Hospital - Imperial College Healthcare NHS Trust (A. Taylor, 1); Poole-Poole Hospital NHS Foundation Trust (V. Laurence, M. Flubacher, 1); Reading-Royal Berkshire Hospital (H. O'Donnell, 1); Stoke-on-Trent-Royal Stoke University Hospital (R. Bhana, S. Lupton, 1)

The Netherlands: 145 patients (138 evaluable)
Study Coordinators: C.L. Creutzberg (C.I.), Leiden; R. Kruitwagen, Maastricht; H. Nijman, Groningen; N. Ottevanger, Nijmegen
Group coordinating trial centre: Netherlands Comprehensive Cancer Organisation (IKNL), Leiden
Trial pathologists: H. Hollema, Groningen; V.T. Smit, Leiden
Participating centres and principal investigator(s) (number of patients): University Medical Center Utrecht (I.M. Jurgenliemk-Schulz, 20); Maastro Clinic, Maastricht (L.C.H.W. Lutgens, 17); University Medical Center Groningen (E. Pras, 15); Leiden University Medical Center (C.L. Creutzberg, R. Nout, 15); University Medical Center Radboud, Nijmegen (J.W.H. Leer, A. Snyers, 11); Academic Medical Center, Amsterdam (A.L.J. Uitterhoeve, G.H. Westerveld, 9); Medical Spectre Twente, Enschede (J.J. Jobsen, 9); Radiotherapy institute Friesland, Leeuwarden (A. Slot, 9); Erasmus Medical Center Rotterdam (J.W.M. Mens, 8); Medical Center Haaglanden/ Radiotherapy Centre West (T.C. Stam, P.C.M. Koper, 7); Netherlands Cancer Institute, Amsterdam (B. van Triest, 6); Radiotherapy Group, Arnhem (E.M. van der Steen-Banasik, 6); Radiotherapy Institute Verbeeten, Tilburg (K.A.J. de Winter, 6); Radiotherapy Group, Deventer (S. van de Pol, 3); Catharina Hospital, Eindhoven (H.A. van den Berg, 3); VU Medical Centre, Amsterdam (O.W.M. Meijer, 1)

Australia & New Zealand: 122 patients (118 evaluable)
ANZGOG Study Coordinators: L. Mileshkin (P.I.) Melbourne; M. Quinn, Melbourne; P. Khaw Melbourne; I.Kolodziej, Sydney
Group coordinating trial centre: NHMRC Clinical Trials Centre, Sydney
Trial pathologist: J.Pyman, Melbourne
Participating centres and principal investigator(s) (number of patients): Peter MacCallum Cancer Centre, Melbourne, Victoria, Australia (L. Mileshkin, P.Khaw; 31); Monash Cancer Centre (Monash Medical Centre), East Bentleigh,

Victoria, Australia (P. Khaw/G. Goss, 20); Westmead Hospital, Wentworthville, NSW, Australia (G. Wain, 15); Auckland City Hospital , Auckland, New Zealand (S. Brooks, 13); Wellington Blood & Cancer Centre, Wellington, New Zealand (C. Johnson, 11); Calvary Mater Newcastle, Newcastle, Australia (A. Capp, 8); Christchurch Hospital, Canterbury, New Zealand (M. Vaughan, 4); Royal Hobart Hospital, Hobart, Tasmania, Australia (P. Blomfield, 3); Palmerston North Hospital, Palmerston North, New Zealand (C. Hardie, 3); Royal North Shore Hospital, St Leonards, NSW, Australia (M. Stevens, 3); Waikato Hospital, Hamilton, New Zealand (M. Kuper, 2); Royal Brisbane &Women's Hospital, Brisbane, QLD, Australia(R. Cheuk, 2); Liverpool Hospital, Liverpool, NSW, Australia (S. Vinod, 2); Mater Hospital Brisbane , South Brisbane, QLD, Australia(C. Shannon/J. Ramsay, 2); Wollongong Hospital, Wollongong, NSW, Australia (A. Glasgow, 2); Townsville Hospital, Townsville, QLD, Australia (S. Hewitt, 1)

Italy: 103 patients (98 evaluable)
MaNGO Study Coordinators: R. Fossati (P.I.) Milano; D. Katsaros, Torino; A. Colombo, Lecco Group coordinating trial centre: Istituto di Ricerche Farmacologiche Mario Negri, Milano
Trial pathologists: S. Carinelli, Milano; C. Di Tonno, Milano
Participating centres and principal investigator(s) (number of patients): Torino - S. Anna Hospital (S. Gribaudo, M. Mitidieri, 33); Lecco - Ospedale A. Manzoni (R. D'Amico, 24); Monza - S. Gerardo Hospital (S. Meregalli, A. A. Lissoni, 16); Torino - Ospedale Umberto I (A. Ferrero, 7); Padova – Istituto Oncologico Veneto / Mirano, Venezia – Azienda ULSS 13 (L. Corti, G. Artioli, 6); Varese - H. Del Ponte University of Insubria (C. Apolloni, 4); Ravenna - Ospedale S. Maria delle Croci (D. Turci, 4); Brescia – Spedali Civili (G. Tognon, 2); Como – ASST Lariana Ospedale S. Anna (E. Bianchi, 2); Meldola - Istituto Scientifico Romagnolo per lo Studio e la Cura dei Tumori (E. Bianchi, 2); Genova – IRCCS San Martino IST (M. Bruzzone, 1); Milano - ASST Grande Ospedale Metropolitano Niguarda (S. Siena, 1); Palermo – AOR Villa Sofia-Cervello (N. Varsellona, 1)

Canada: 65 patients (65 evaluable)
CCTG Study Coordinators: A. Fyles Toronto, Ontario; P. Bessette, Sherbrooke, Quebec
Group coordinating trial centre: Canadian Cancer Trials Group, Kingston, Ontario
Trial pathologist: M. McLachlin, London, Ontario
Participating centres and principal investigator(s) (number of patients): Sherbrooke-Centre Hosp. Universitaire de Sherbrooke (P. Bessette, 18); Montreal-Hopital Notre-Dame de Montreal (D. Provencher, 11); Calgary-Tom Baker Cancer Centre (P. Ghatage, 9); Halifax-Queen Elizabeth II Health Sciences Centr (P. Rittenberg, 8); Montreal-McGill Oncology Montreal (L. Souhami, 7); Toronto Sunnybrook Health Sciences Centre (G. Thomas, 7); Quebec-Hotel-Dieu de Quebec (M. Plante, 2); London London Health Sciences Centre (A. Hammond, 1); St John's-Dr. H. Bliss Murphy Cancer Centre (P. Power, 1); Toronto-Princess Margaret Hospital (A. Fyles, 1)

France: 67 patients (64 evaluable) FEDEGYN
Study Coordinator: Chr. Haie-Meder (P.I.) Paris
Group coordinating trial centre: UNICANCER, Paris
Trial pathologist: P. Duvillard, Paris
Participating centres and principal investigator(s) (number of patients): Besancon-Hopital Jean Minjoz (M-H Baron, 10); Rouen-Centre Henri Becquerel (Hanzen, 9); Saint Herblain Centre Rene Gauducheau (D. Berton-Rigaud, 8); Limoges-CHU Limoges (Pr. N. Tubiana-Mathieu, 6); Bordeaux-Institut Bergonie (L. Thomas, 5); Reims-Institut Jean Godinot (A. Savoye, S. Maillard, 5); Dijon Centre Georges Francois Leclerc (K. Peignaux, 4); Paris/ Villejuif-Institut Gustave Roussy (C. Haie Meder, 4); Clermont-Ferrand-Centre Jean Perrin (C. Benoit, 3); Montpellier-Centre Val d'Aurelle (C. Kerr, 3); Toulouse Cedex-Institut Claudius Regaud (L. Gladieff, 3); Caen-Centre Francois Baclesse (D. Lerouge, 2); Nice Cedex Centre Antoine Lacassagne (P. Follana, 2); Marseille-Institut Paoli Calmettes (M. Cappiello, 1); Strasbourg Centre Paul Strauss (T. Petit, 1); Tours Cedex-CHU de Tours - Hopital Bretonneau (I. Barillot, 1)

CONSORT diagram of the PORTEC-3 trial

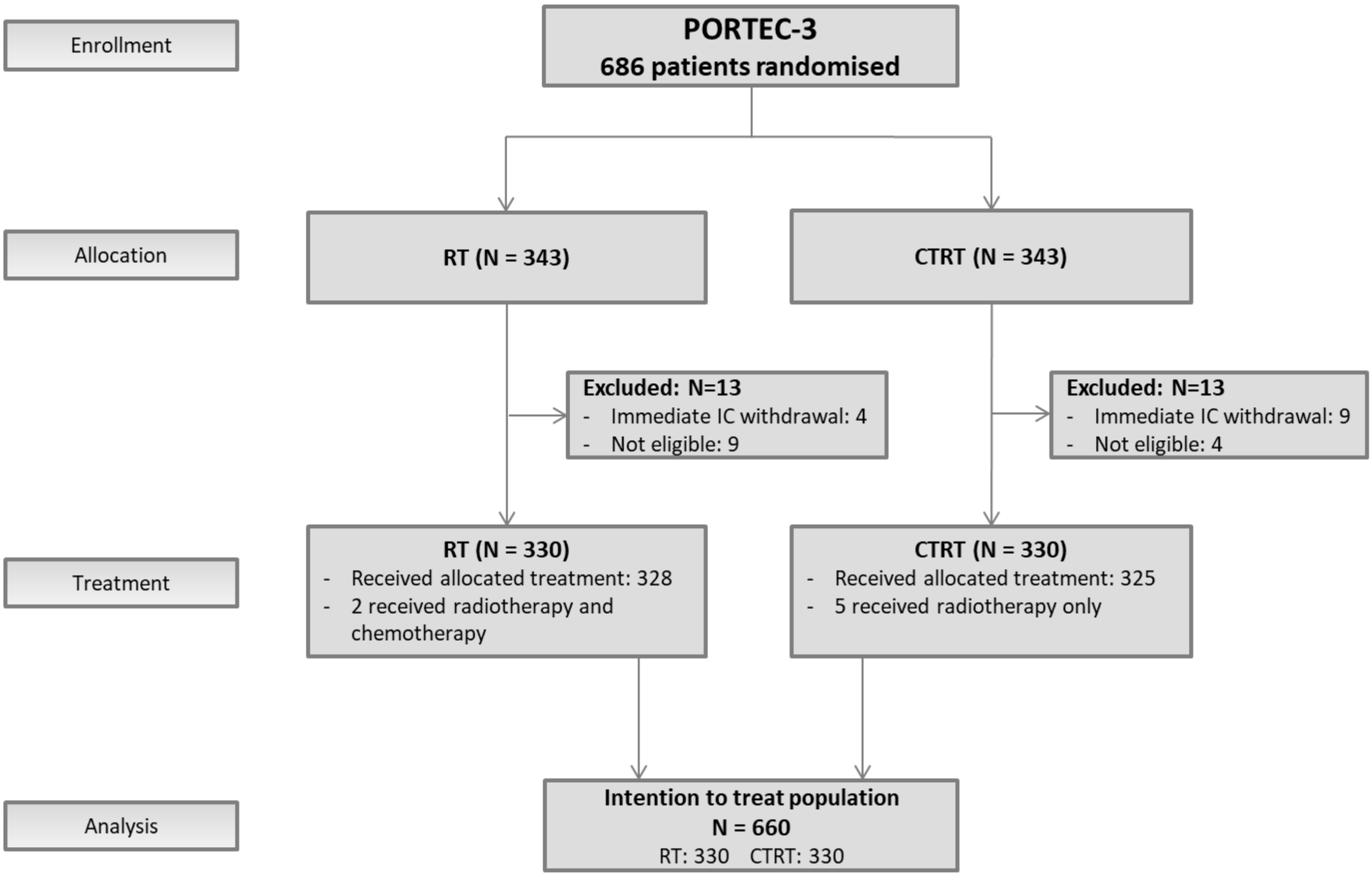

Table 1a. Multivariable analysis of prognostic factors for overall survival.

	N	Events	5y OS (95% CI)	10y OS (95% CI)	HR all patients (95% CI)	p-value	N	HR with molecular class (95% CI)	p-value
Total	660								
Arm									
Radiotherapy	330	104	76.5% (72.1 – 81.3)	67.3% (62.3 – 72.7)			201		
Chemoradiotherapy	330	82	81.4% (77.3 – 85.7)	74.4% (69.8 – 79.4)	0.69 (0.52-0.92)	0.013	210	0.72 (0.50-1.04)	0.079
Age									
<60 years	268	41	89.2% (8505 – 93.0)	84.1% (79.8 – 88.7)			178		
60-69	272	94	75.0% (70.0 – 80.4)	64.1% (58.5 – 70.2)	2.44 (1.69-3.56)	<0.001	169	2.31 (1.43-3.74)	<0.001
≥70 years	120	51	65.0% (57.0 – 74.1)	56.7% (48.3 – 66.5)	3.24 (2.19-5.16)	<0.001	64	2.78 (1.54-5.00)	<0.001
Stage									
I-II	365	81	82.9% (0.792 - 0.869)	77.2% (73.0 – 81.7)			233		
III	295	105	74.0% (0.691 - 0.792)	63.0% (5.76 - 68.9)	2.47 (1.80-3.38)	<0.001	178	2.45 (1.64-3.65)	<0.001
Histology and grade									
Endometroid grade 1-2	258	55	86.7% (82.6 – 91.0)	77.5% (72.4 – 83.0)			161		
Endometroid grade 3	213	57	78.8% (73.4 – 84.5)	72.4% (66.5 – 78.8)	1.75 (1.18-2.59)	0.0056	132	1.44 (0.84-2.46)	0.19
Serous / clear cell	189	74	68.6% (62.3 – 75.6)	60.0% (53.3 – 67.5)	2.45 (1.67-3.58)	<0.001	118	1.10 (0.60-2.02)	0.76
LVSI (any)									
Absent	271	65	83.6% (793 – 88.2)	75.0% (69.9 – 80.1)			155		
Present	389	121	75.7% (716 – 8.12)	68.0% (63.4 – 72.9)	1.28 (0.93-1.74)	0.13	256	1.47 (1.01-2.36)	0.071
Molecular class	411								
MMRd	139	37	75.5% (68.6 – 83.2)	71.5% (64.0- 79.8)			139		
POLEmut	51	1	98.0% (94.3 – 100)	98.0% (94.3 – 100)			51	0.070 (0.010-0.54)	0.009
P53abn	99	55	48.9% (39.9 – 59.9)	43.2% (34.3 – 54.4)			99	2.29 (1.46-3.99)	<0.001
NSMP	122	26	85.6% (79.4 – 92.2)	75.6% (67.7 – 84.4)			122	0.69 (0.39-1.16)	0.16

Overall survival rates are displayed for all study patients at 5- and 10-years follow-up. Hazard ratios of the multivariable analysis using cox proportional hazard model adjusted for participating group and lymphadenectomy are displayed for all patients and the subset with known molecular class.

Table 1b. Multivariable analysis of prognostic factors for recurrence-free survival.

	N	Events	5y RFS (95% CI)	10y RFS (95% CI)	HR all patients (95% CI)	p-value*	N	HR with molecular class (95% CI)	p-value*
Total	660								
Arm									
Radiotherapy	330	107	69.4%(64.6 – 74.6)	67.1%(62.2 – 72.4)			201		
Chemoradiotherapy	330	89	76.1%(71.9 – 81.2)	72.4%(67.7 – 77.5)	0.71 (0.53-0.94)	0.016	210	0.64 (0.45-0.91)	0.013
Age									
<60 years	268	58	81.3%(76.8 – 86.1)	78.2%(73.4 – 83.4)			178		
60-69	272	90	68.4%(63.0 – 74.2)	65.9%(60.4 – 71.9)	1.71 (1.23-2.40)	<0.001	169	1.54 (1.01-2.35)	0.045
≥70 years	120	48	64.0%(55.9 – 73.2)	59.4%(51.1 – 69.0)	2.16 (1.45-3.23)	<0.001	64	1.69 (0.99-2.90)	0.055
Stage									
I-II	365	83	79.3%(75.2 – 83.6)	76.9%(72.7 – 81.4)			233		
III	295	113	64.9%(59.7 – 70.7)	60.9%(55.5 – 66.8)	2.59 (1.90-3.54)	<0.001	178	2.52 (1.71-3.72)	<0.001
Histology and grade									
Endometroid grade 1-2	258	66	79.3% (74.5 – 84.4)	73.8% (68.6 – 79.5)			161		
Endometroid grade 3	213	61	73.0% (67.3 – 79.3)	71.0% (65.1 – 77.4)	1.44 (1.00-2.09)	0.053	132	1.05 (0.63-1.76)	0.84
Serous / clear cell	189	69	64.0% (57.4 – 71.3)	62.8% (56.2 – 70.2)	1.98 (1.37-2.86)	<0.001	118	0.95 (0.53-1.71)	0.87
LVSI (any)									
Absent	271	65	78.3%(73.5 – 83.4)	75.4%(70.4 – 80.8)			155		
Present	389	131	69.2%(64.7 – 74.0)	65.9%(61.3 – 70.8)	1.42 (1.05-1.93)	0.023	256	1.55 (1.05-2.30)	0.029
Molecular class									
MMRd	139	35	74.7%(67.7 – 82.3)	74.7%(67.7 – 82.3)			139		
POLEmut	51	1	98.0%(94.3 - 100)	98.0%(94.3 - 100)			51	0.072 (0.01-0.53)	0.009
P53abn	99	54	49.0%(40.1 – 60.0)	44.7%(35.8 – 55.8)			99	2.83 (1.71-4.68)	<0.001
NSMP	122	39	76.2%(69.0 – 84.2)	67.6%(59.7 – 76.5)			122	1.12 (0.70-1.80)	0.63

Recurrence-free survival rates are displayed for all study patients at 5- and 10-years follow-up. Hazard ratios of the multivariable analysis using cox proportional hazard model adjusted for participating group and lymphadenectomy are displayed for all patients and the subset with known molecular class.

Table 2. Five-year adverse events reported in the PORTEC-3 trial

Table reprinted from Table 3 in: De Boer SM, Powell ME, Mileshkin L, et al. Adjuvant chemoradiotherapy versus radiotherapy alone in women with high-risk endometrial cancer (PORTEC-3): patterns of recurrence and post-hoc survival analysis of a randomised phase 3 trial. Lancet Oncol. 2019 Sep;20(9):1273-1285.

Adverse events reported at 60 months after randomisation

	**CTRT n=201; RT n=187**					
	**Grade 2**			**Grade 3-4**		
	**CTRT** n (%)	**RT** n (%)	**p***	**CTRT** n (%)	**RT** n (%)	**p#**
**Any**	59 (29)	33 (18)	0·002	17 (8)	10 (5)	0·24
**Any grade 3**	na	na		16 (8)	10 (5)	
**Any grade 4**	na	na		1 (0)	0 (0)	
Allergic reaction/ hypersensitivity	0 (0)	0 (0)	0·48	0 (0)	1 (1)	0·48
Auditory/hearing	4 (2)	1 (1)	0·29	2 (1)	1 (1)	1·00
Fatigue	0 (0)	3 (2)	0·11	0 (0)	0 (0)	1·00
Hypertension	16 (8)	16 (9)	0·87	4 (2)	4 (2)	1·00
Lymphatics (edema)	5 (2)	0 (0)	0·06	0 (0)	0 (0)	1·00
**GI -** any	16 (8)	9 (5)	0·19	2 (1)	1 (1)	1·00
Diarrhea	7 (3)	7 (4)	1·00	0 (0)	0 (0)	1·00
Ileus/obstruction	2 (1)	1 (1)	0·37	2 (1)	0 (0)	0·50
**Hematological** - any	5 (2)	5 (3)	1·00	0 (0)	0 (0)	1·00
Lymphocytes	3 (1)	4 (2)	0·74	0 (0)	0 (0)	1·00
**Infection** (without neutropenia)	2 (1)	0 (0)	0·12	2 (1)	0 (0)	0·50
**Neuropathy -** any	13 (6)	0 (0)	<0·001	1 (0)	0 (0)	1·01
Neuropathy - motor	1 (0)	0 (0)	0·50	1 (0)	0 (0)	1·02
Neuropathy - sensory	12 (6)	0 (0)	<0·001	1 (0)	0 (0)	1·03
**Neurology**						
other	1 (0)	0 (0)	0·25	2 (1)	0 (0)	0·50
**Pain** - any	13 (6)	5 (3)	0·15	3 (1)	3 (2)	1·00
Joint pain	7 (3)	2 (1)	0·14	2 (1)	1 (1)	1·00
Muscle pain	1 (0)	1 (1)	0·61	0 (0)	1 (1)	0·48
Back/pelvic/limbs	0 (0)	2 (1)	0·11	0 (0)	1 (1)	0·48
abdomen/cramps	2 (1)	0 (0)	0·12	2 (1)	0 (0)	0·50
**Musculoskeletal** - other	0 (0)	1 (0)	1·00	1 (0)	0 (0)	1·00
**Pulmonary** - dyspnea	2 (1)	0 (0)	0·25	0 (0)	0 (0)	1·00
**GU**						
Incontinence	8 (4)	9 (5)	1·00	0 (0)	0 (0)	1·00
Urinary frequency	8 (4)	2 (1)	0·14	0 (0)	1 (1)	0·48
**Constitutional symptoms**						
Fatigue	0 (0)	3 (2)	0·11	0 (0)	0 (0)	1·00
Other	0 (0)	0 (0)	1·00	1 (0)	0 (0)	1·00
**Other toxicity**	4 (2)	4 (2)	1·00	3 (1)	2 (1)	1·00

Abbreviations: CTRT, combined chemotherapy and radiotherapy; RT, radiotherapy; GI, gastro-intestinal; GU, genito-urinary; CTCAE v3.0, Common Terminology Criteria for Adverse Events version 3.0
AE were calculated at each time point. Per AE, the maximum grade per patient was calculated (worst ever by patient).

p* = significant level < 0.01 for grade ≥2, 3 and 4; p# = significant level <0.01 for grade 3 and 4.

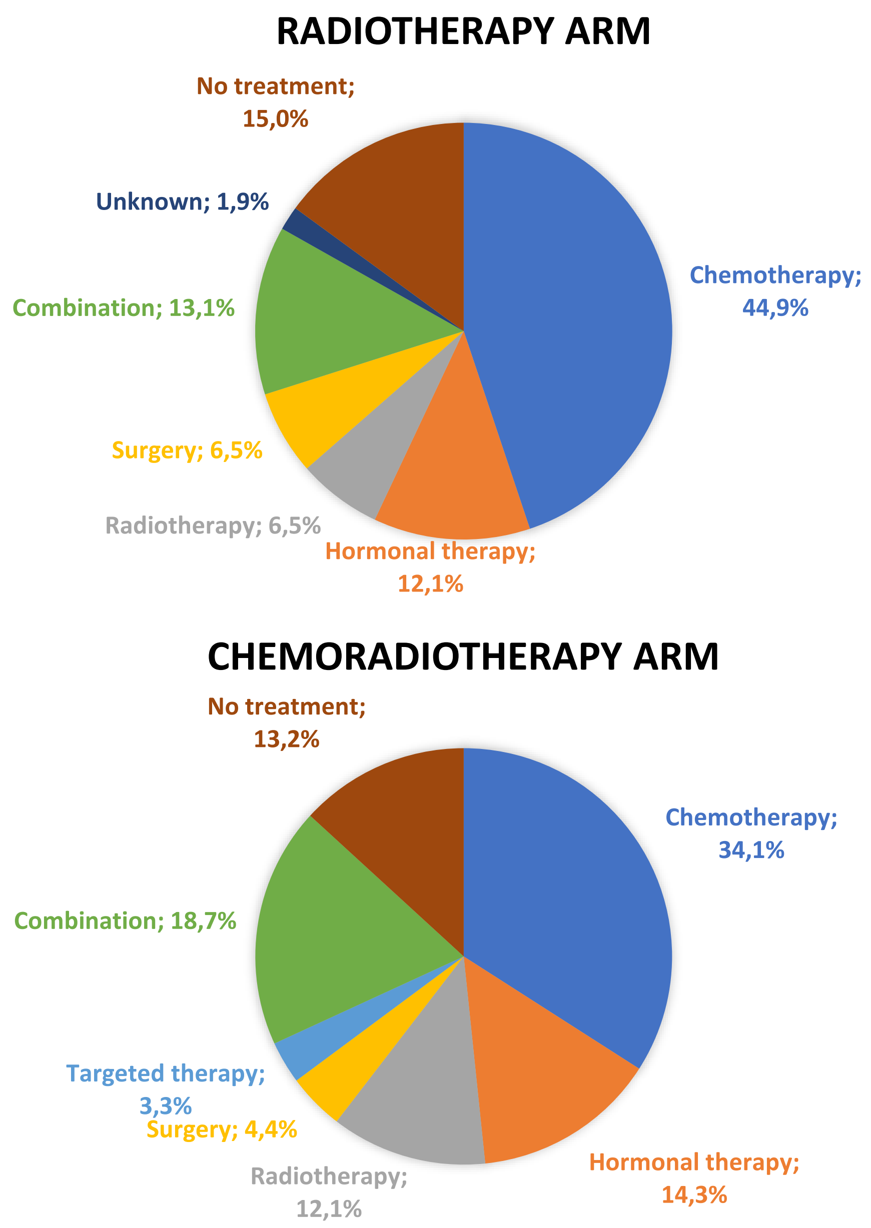

Figure 1. Treatment for first recurrence by treatment arm.

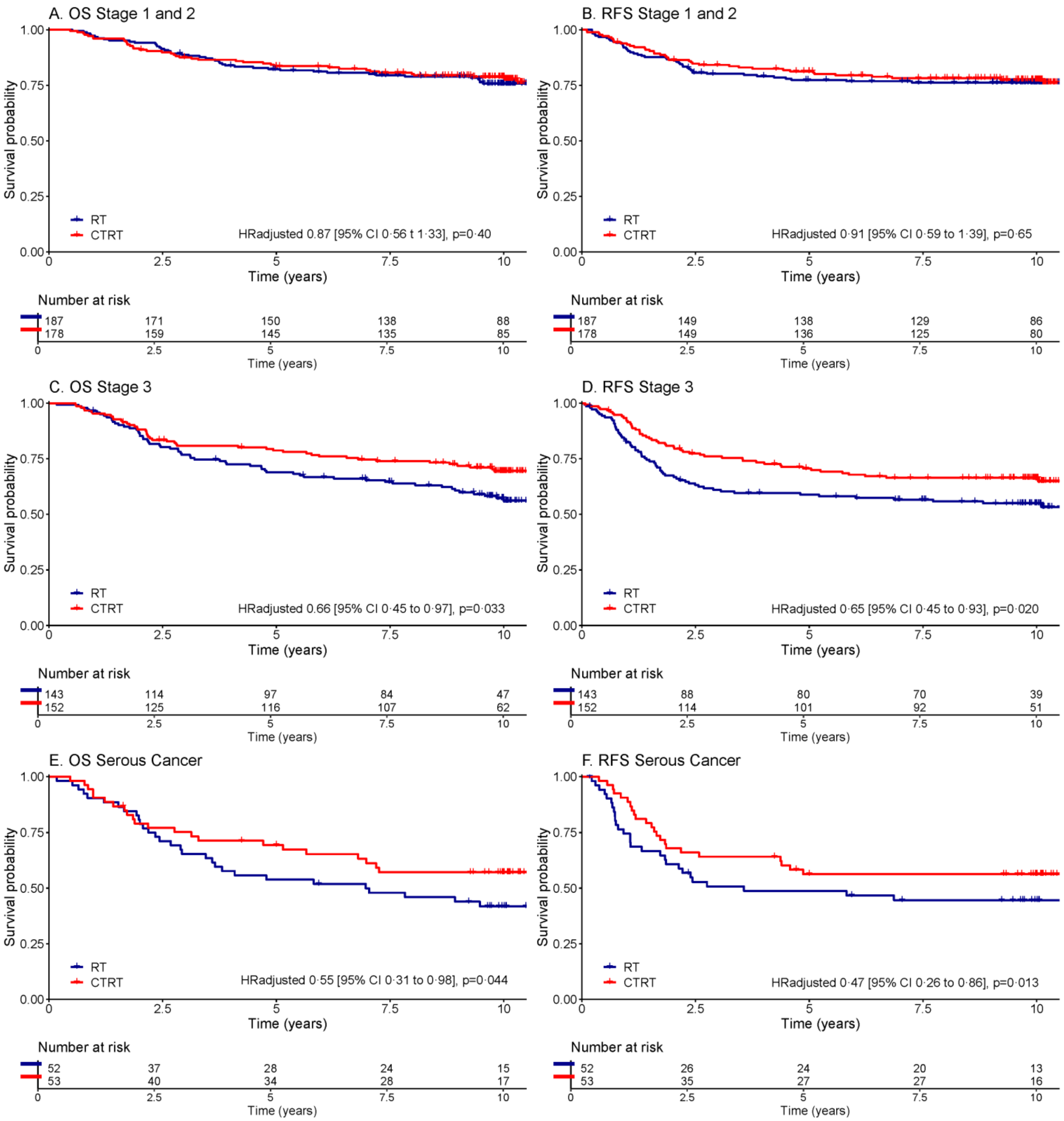

Figure 2. Overall survival and recurrence-free survival in patients with (A-B) stage 1 and 2, (C-D) stage 3 or (E-F) serous endometrial cancer by treatment arm.

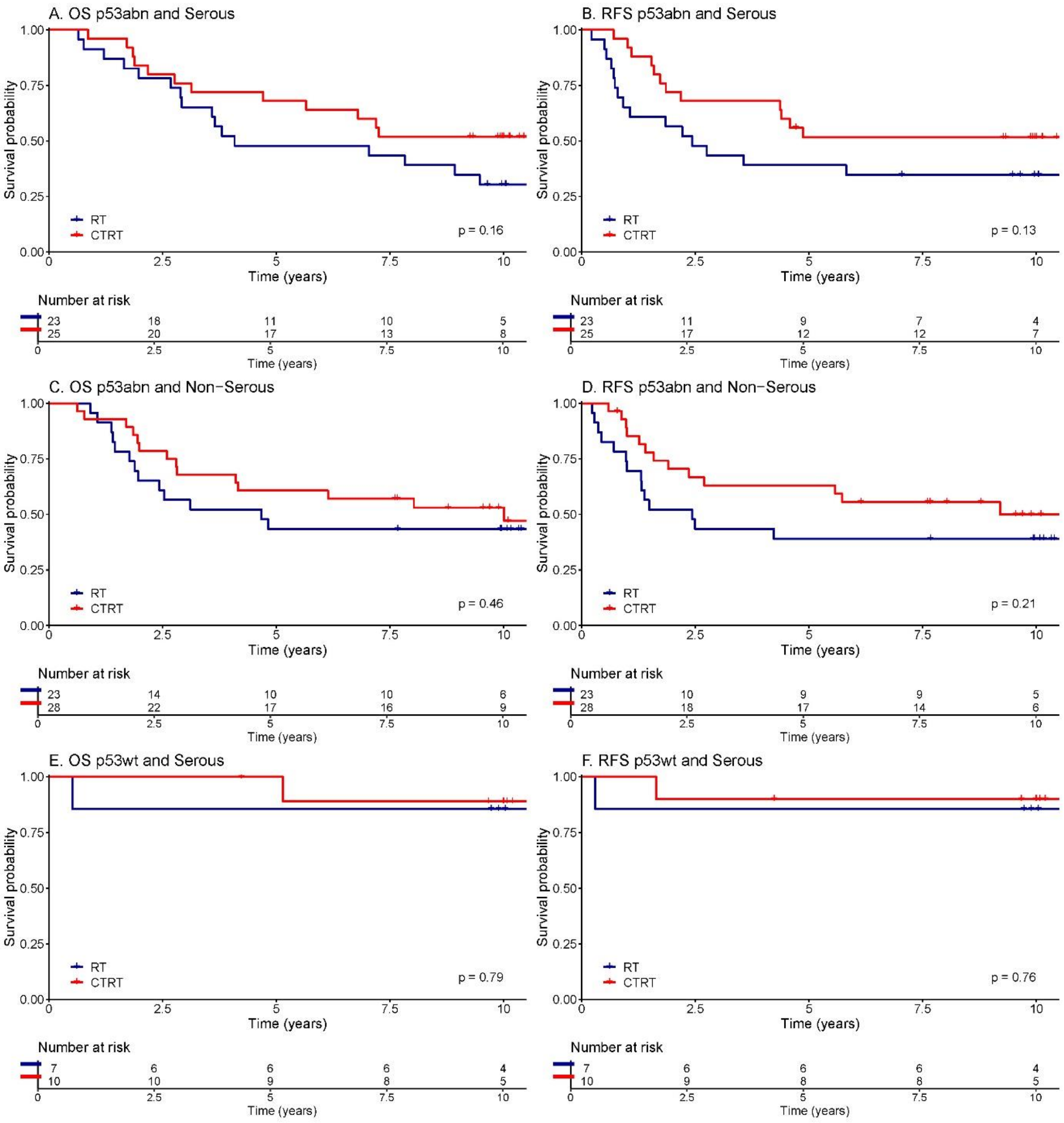

Figure 3. Overall survival and recurrence-free survival in patients with combinations of p53abn versus p53wt and serous versus non-serous endometrial cancer: (A-B) p53abn and serous, (C-D) p53abn and non-serous, and (E-F) p53wt and serous. The *p*-value of the log-rank test is displayed.

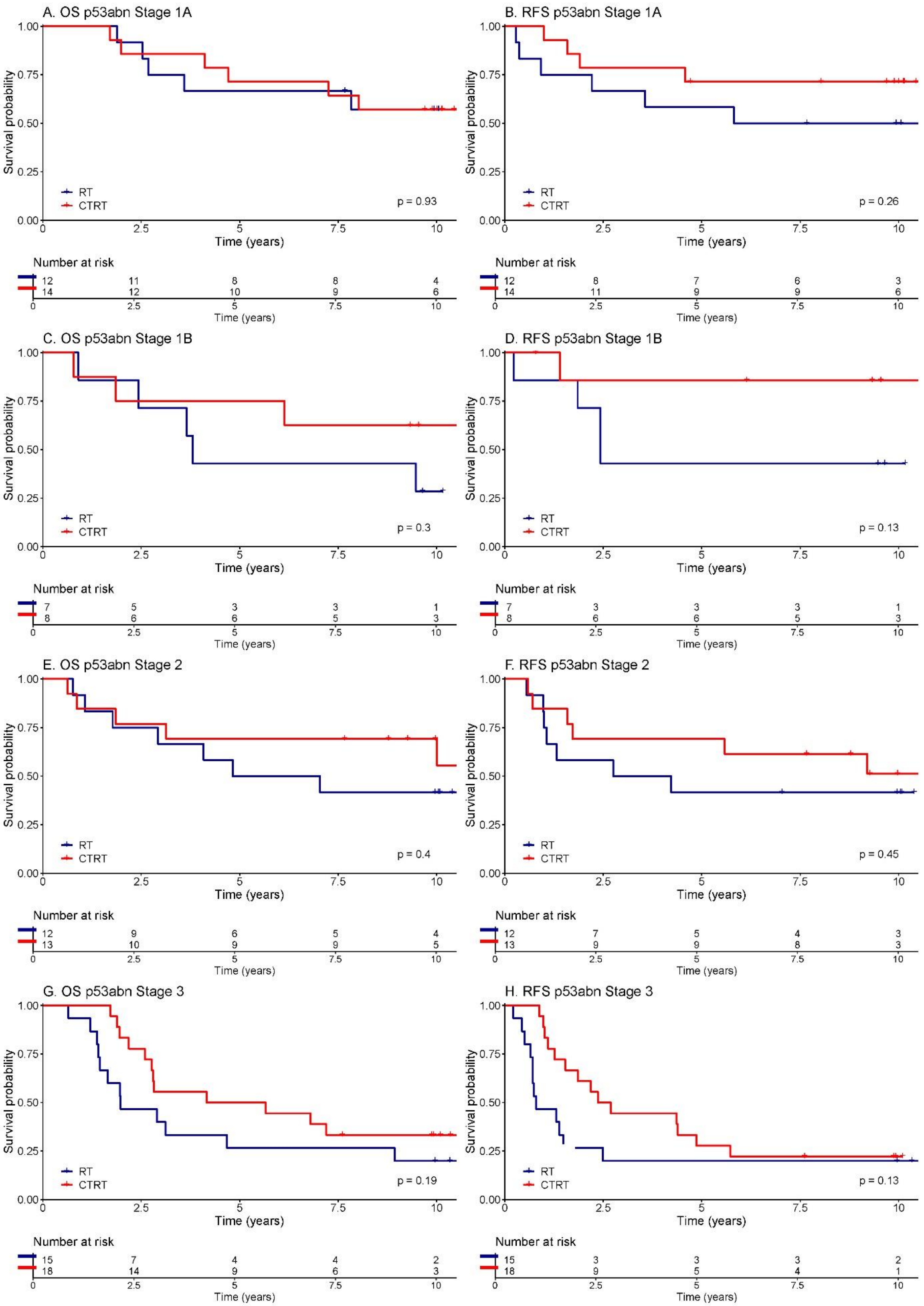
A. OS p53abn Stage 1A
B. RFS p53abn Stage 1A
C. OS p53abn Stage 1B
D. RFS p53abn Stage 1B
E. OS p53abn Stage 2
F. RFS p53abn Stage 2
G. OS p53abn Stage 3
H. RFS p53abn Stage 3
Survival probability
Time (years)
RT
CTRT
p = 0.93
p = 0.26
p = 0.3
p = 0.13
p = 0.4
p = 0.45
p = 0.19
p = 0.13
Number at risk
12 11 8 8 4
14 12 10 9 6
12 8 7 6 3
14 11 9 9 6
7 5 3 3 1
8 6 6 5 3
7 3 3 3 1
8 6 6 5 3
12 9 6 5 4
13 10 9 9 5
12 7 5 4 3
13 9 9 8 3
15 7 4 4 2
18 14 9 6 3
15 3 3 3 2
18 9 5 4 1

Figure 4. Overall survival and recurrence-free survival in patients with p53-abnormal endometrial cancer: (A-B) Stage 1A, (C-D) Stage 1B (E-F) Stage 2 and (G-H) Stage 3. The *p*-value of the log-rank test is displayed.

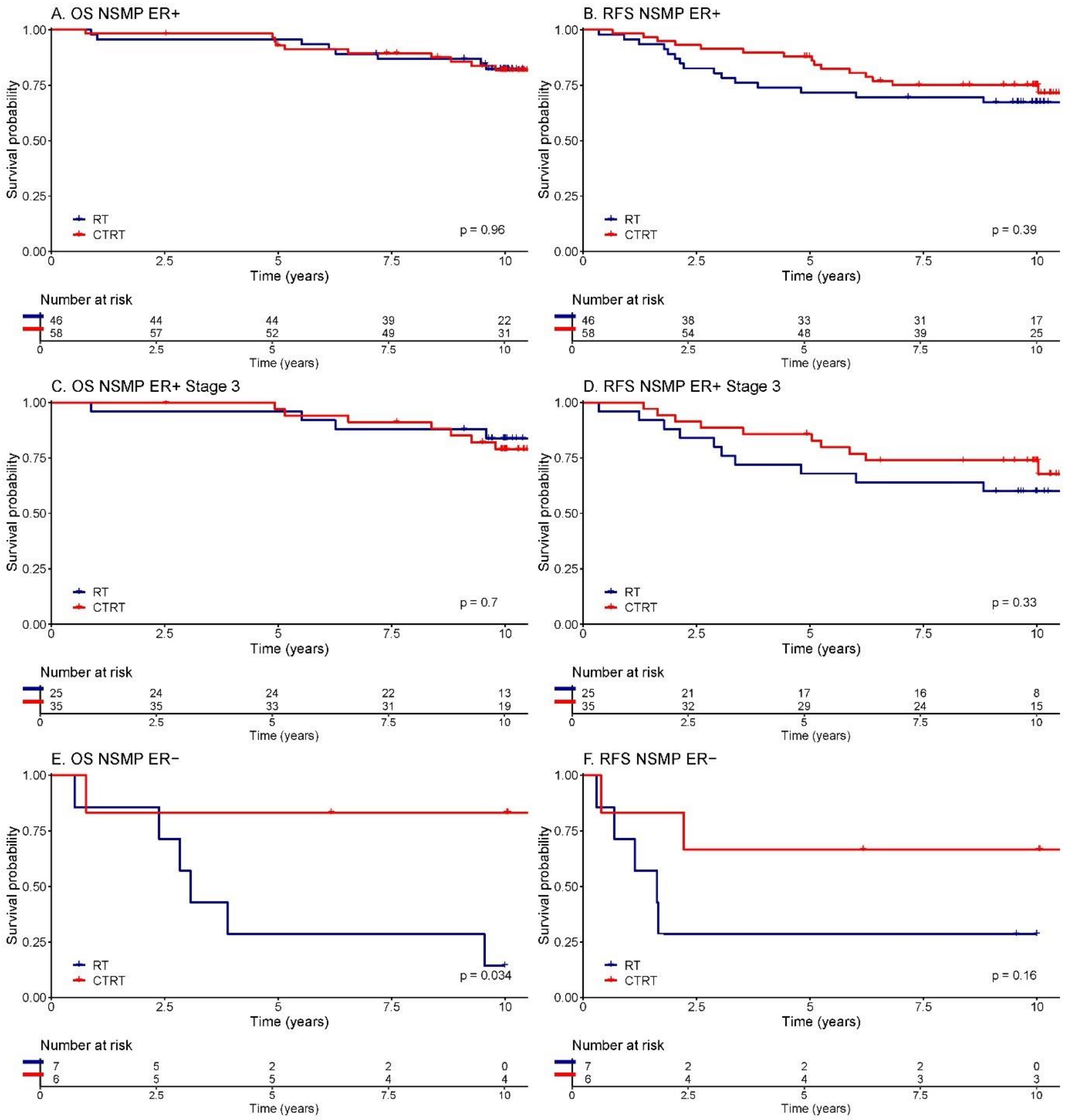

Figure 5. Overall survival and recurrence-free survival in patients with NSMP tumours: (A, B) ER-positive, (C-D) Stage 3 ER-positive, (E-F) ER-negative, (G-H) Grade 3, and (I-J) ER-positive Grade 3. The *p*-value of the log-rank test is displayed. *(continued on next page)*

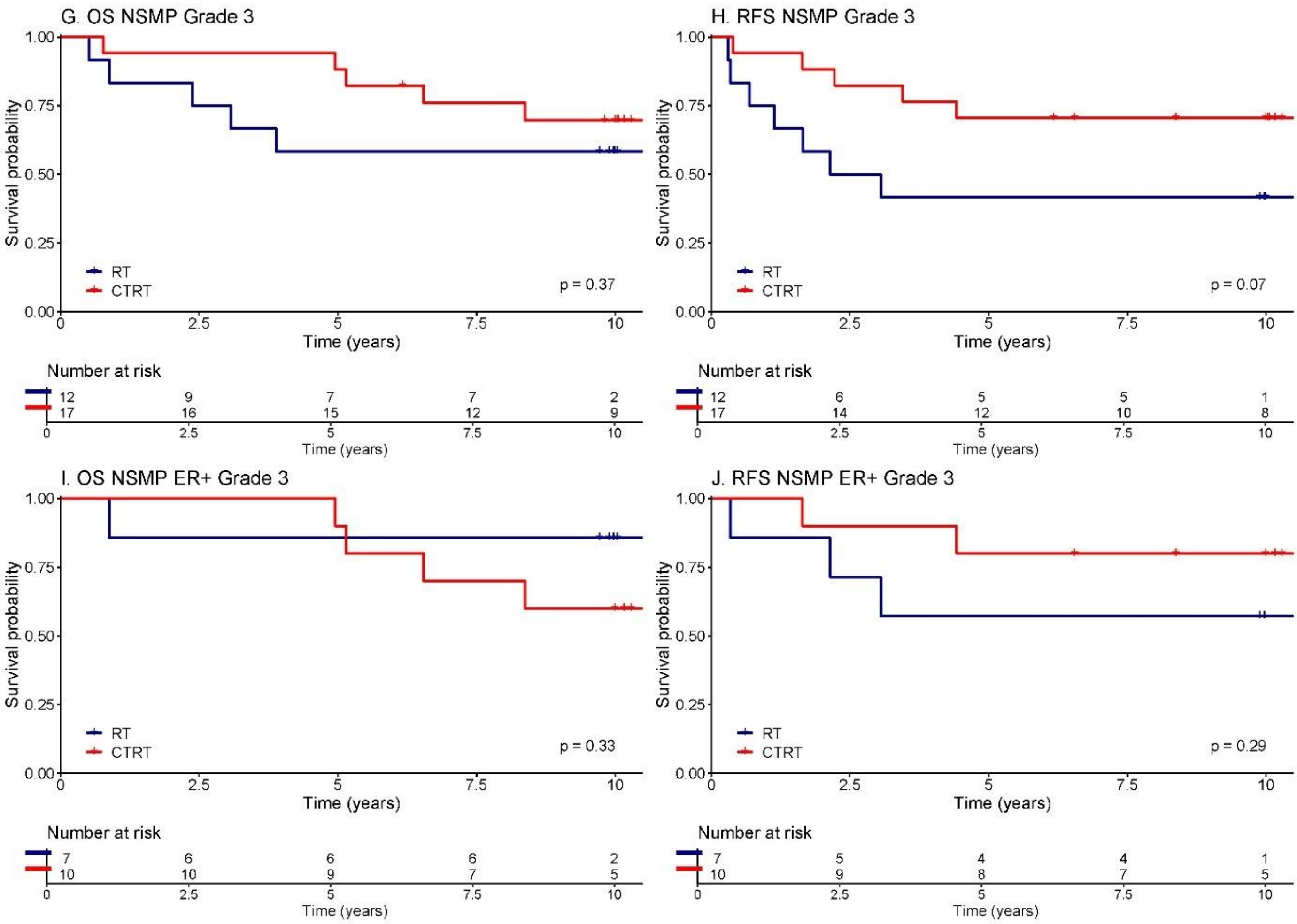

Figure 5 (continued from previous page). Overall survival and recurrence-free survival in patients with NSMP tumours: (G-H) Grade 3, and (I-J) ER-positive Grade 3. The p-value of the log-rank test is displayed.

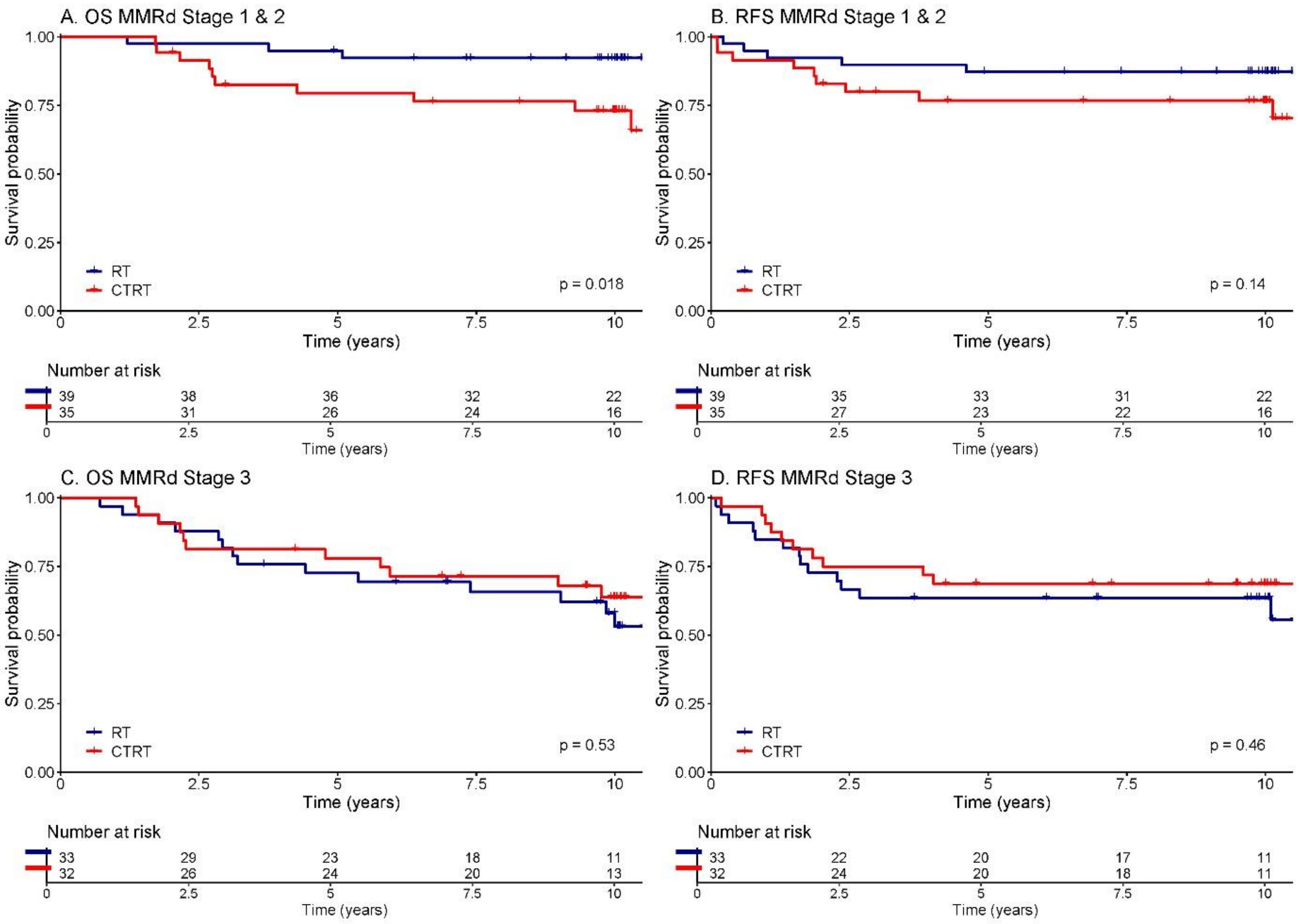

Figure 6. Overall survival and recurrence-free survival in patients with MMRd endometrial cancer: (A-B) Stage 1 and 2, (C-D) Stage 3. The *p*-value of the log-rank test is displayed.

Randomized Phase III Trial Comparing Concurrent Chemoradiation and Adjuvant Chemotherapy with Pelvic Radiation Alone in High Risk and Advanced Stage Endometrial Carcinoma: PORTEC-3

An international Intergroup trial

Trial Management Group:

NL Study Coordinators:	C.L. Creutzberg (C.I.)	Leiden
	R. Kruitwagen	Maastricht
	H. Nijman	Groningen
	N. Ottevanger	Nijmegen
UK Study Coordinators:	M. Powell (P.I.)	London
	P. Blake	London
	H. Kitchener	Manchester
	J. Ledermann	London
Trial Statistician and QoL	H. Putter	Leiden
Pathology NL:	H. Hollema	Groningen
	V.T.H.B.M. Smit	Leiden
Pathology UK:	G. Wilson	Manchester
	N. Singh	London

Data Centre NL: K.W. Verhoeven-Adema, Central Data Manager & Trial Coordinator
IKNL Leiden Trial Office, C7-143, LUMC,
P.O. Box 9600, 2300 RC Leiden
Tel: +31 71 526 3052
Fax: +31 71 526 6712
email: k.verhoeven@iknl.nl

Data Centre UK: Cancer Research UK and UCL Cancer Trials Centre
90 Tottenham Court Road, London W1T 4TJ
Tel: +44 20 7679 9890
Fax: +44 20 7679 9871
email: portec-3@ctc.ucl.ac.uk

Approval status: KWF-CKTO, approved (CKTO 2006-04)
CME LUMC, approved (P06.031)
CTAAC, UK, approved (C7925/A8659)
NCIC CTG, approved (NCIC CTG EN 7)
UNICANCER, approved (FEDEGYN 01/0904)

Trials Register: ISRCTN14387080 www.controlled-trials.com/ISRCTN14387080
EudraCT number: 2007-004917-33

Version: 07 November 2016

Participating groups

National Cancer Institute of Canada Clinical Trials Group

NCIC CTG Study Coordinators:	R. Meyer	Kingston, Ontario
	A. Fyles	Toronto, Ontario
	P. Bessette	Sherbrooke, Quebec
Pathology NCIC CTG:	M. McLachlin	London, Ontario

Data Centre NCIC CTG:
NCIC Clinical Trials Group, Cathy Davidson
Queens University, 10 Stuart Street,
Kingston, Ontario, K7L 3N6, Canada
Tel: +613-533-6430
Fax: +613-533-2941
Email: cdavidson@ctg.queensu.ca

ANZGOG, Australia and New-Zealand

ANZGOG Study Coordinators:	L. Mileshkin (P.I.)	Melbourne
	M. Quinn	Melbourne
	P. Khaw	Melbourne

Data Centre ANZGOG:
ANZGOG Coordinating Centre
NHMRC Clinical Trial Centre, Locked Bag 77
Camperdown NSW 1450, Australia
Tel: +61 2 9562 5000
Fax: +61 2 9562 5094
Email: PORTEC-3@ctc.usyd.edu.au

MaNGO group, Italy

MaNGO Study Chair: R. Fossati (P.I.) Milano

Data Centre MaNGO:
Emanuele Negri & Simona Stupia, Dipartimento di Oncologia
Istituto di Ricerche Farmacologiche "Mario Negri"
Via Giuseppe La Masa, 19
20156 Milano, Italy
Tel. +39 02 39014519
Fax +39 02 33200231
Email: stupia@marionegri.it

UNICANCER (FEDEGYN Group), France

UNICANCER Study Coordinator:	Chr. Haie-Meder (P.I.)	Paris
Pathology Unicancer:	P. Duvillard	Paris

Data Centre UNICANCER:
Beata JUZYNA, Project Manager
R&D UNICANCER
101 rue de Tolbiac – 75654 PARIS cedex 13
TEL : +33 (0)1 44 23 55 72
FAX : +33 (0)1 44 23 55 69
Email: b-juzyna@unicancer.fr

Independent Data and Safety Monitoring Board:
Prof. J. Yarnold (Clinical Oncology, NCRI UK), chair
W.L.J. van Putten (Biostatistics)
Dr L.V.A.M. Beex (Medical Oncology)
Prof. N. James (Clinical Oncology, NCRI UK)
Dr M.J.M. Olofsen-van Acht (Radiation Oncology)
Prof. D. Rischin (Medical Oncology, ANZGOG)
Dr. W. Parulekar (Medical Oncology, NCIC CTG)

Trial website: **www.clinicalresearch.nl/portec3**

Table of Contents

Page

1. Study synopsis …… 6
2. Introduction …… 7
   - 2.1. Radiotherapy for intermediate risk endometrial carcinoma …… 7
   - 2.2. High-risk endometrial carcinoma …… 7
   - 2.3. Unfavorable histologic types …… 8
   - 2.4. Surgery …… 8
   - 2.5. Adjuvant chemotherapy …… 10
   - 2.6. Chemoradiation …… 11
3. Trial objectives …… 12
4. Trial design …… 12
5. Patient selection …… 12
   - 5.1. Inclusion criteria …… 13
   - 5.2. Exclusion criteria …… 13
6. Summary of Treatment Schedule …… 14
7. Staging and Treatment …… 14
   - 7.1. Staging …… 14
   - 7.2. Surgery …… 14
   - 7.3. Radiotherapy …… 15
     - 7.3.1. External beam pelvic radiation …… 15
     - 7.3.2. Vaginal brachytherapy …… 16
     - 7.3.3. Radiotherapy quality assurance …… 16
   - 7.4 Chemotherapy …… 17
     - 7.4.1. Concurrent phase …… 17
     - 7.4.2. Adjuvant phase …… 18
     - 7.4.3. Supportive care …… 19
8. Pathology …… 19
   - 8.1. Histopathologic evaluation …… 19
   - 8.2. Central pathology review …… 21
   - 8.3. Translational research …… 21
9. Follow-up, toxicity evaluation and adverse events …… 22
   - 9.1. Follow-up …… 22
   - 9.2. Reasons for going off protocol treatment …… 22
   - 9.3. Adverse events and reporting of adverse events …… 23
     - 9.3.1. Definitions …… 23
     - 9.3.2. Adverse Events (AE) …… 23
     - 9.3.3. Serious Adverse Events (SAE) and SUSAR …… 23
     - 9.3.4. Summary of procedures …… 24
10. Registration and randomisation …… 25
   - 10.1. Registration …… 25
   - 10.2. Randomisation …… 25
11. Quality of life assessment …… 25
12. Statistical considerations …… 26
   - 12.1. Number of patients and power calculation …… 26
   - 12.2. Stopping rule, safety reviews and interim analyses …… 27
   - 12.3. Statistical analysis …… 28
   - 12.4. Statistical analysis of the quality of life assessment …… 28
13. Ethics …… 29

Table of Contents (continued)

Page

14. Trial Insurance 29

15. Publication policy 29

16. List of participating centers 31
    - 16.1. The Netherlands - DGOG.. 31
    - 16.2. UK - NCRI.. 31
    - 16.3. Canada – NCIC CTG 32
    - 16.4. Australia and New Zealand – ANZGOG 33
    - 16.5. Italy – MaNGO group 33
    - 16.6. Austria – Vienna 34
    - 16.7. France – UNICANCER 34

17. References 35

Appendix A. Summary PORTEC-3 37

Appendix B. FIGO staging 38

Appendix C. Performance status (WHO).. 39

Appendix D. Histologic classification and grading system 40

Appendix E. Radiotherapy specifications and quality assurance 42

Appendix F. Common Terminology Criteria for Adverse Events 44

Appendix G. Quality of Life Questionnaires 48

Appendix H. Patient information (Netherlands).. 52

Appendix I. Forms and procedures for collecting data 57

Appendix J. Checklists for investigations before, during and after treatment 58

Appendix K. Group Specific Appendices for the Participating Groups 60
- UK GSA.. 60
- ANZGOG GSA 69
- UNICANCER GSA 75
- NCIC CTG GSA: see separate document

1. STUDY SYNOPSIS

**Title**	Randomised Phase III Trial Comparing Concurrent Chemoradiation and Adjuvant Chemotherapy with Pelvic Radiation Alone in High Risk and Advanced Stage Endometrial Carcinoma: **PORTEC-3**
**Study Design**	Prospective, multicenter, randomized Phase III Intergroup trial led by the Dutch Cooperative Gynecologic Oncology Group, in collaboration with the UK National Cancer Research Institute, NCIC-CTG, ANZGOG, MaNGO Group, and UNICANCER (FEDEGYN Group).
**Primary Study Objectives:**	Establish overall survival and failure-free survival of patients with high-risk and advanced stage endometrial carcinoma, treated after surgery with concurrent radiotherapy and chemotherapy, followed by adjuvant chemotherapy, in comparison with patients treated with pelvic radiation alone.
**Secondary Study Objectives:**	Establish and compare the rates of treatment-related toxicity, quality of life, and pelvic and distant recurrence.
**Inclusion Criteria:**	Histologically confirmed endometrial carcinoma, with one of the following postoperative FIGO 2009 stages and grade:<br>1. stage IA with myometrial invasion, grade 3 with documented LVSI<br>2. stage IB grade 3<br>3. stage II<br>4. stage IIIA or IIIC; or IIIB if parametrial invasion only<br>5. stage IA (with myometrial invasion), IB, II, or III with serous or clear cell histology<br>WHO-performance status 0-2<br>WBC ≥ 3.0 x 10^9/L.<br>Platelets ≥ 100 x 10^9/L.<br>Bilirubin ≤ 1.5 x UNL<br>ASAT/ALAT ≤ 2.5 x UNL<br>Written informed consent
**FIGO 1988:**	During the intial years of the trial, FIGO staging 1988 was used. Inclusion criteria based on FIGO 1988 were:<br>1. stage IB grade 3 with documented LVSI<br>2. stage IC or IIA grade 3<br>3. Stage IIB<br>4. stage IIIA or IIIC (*IIIA based on cytology alone only eligible if grade 3)*<br>5. stage IB or IC, stage II or stage III with serous or clear cell histology
**Exclusion Criteria:**	Uterine sarcoma (including carcinosarcoma)<br>Previous malignancy (except for non-melanomatous skin cancer) < 10 yrs<br>Previous pelvic radiotherapy<br>Hormonal therapy or chemotherapy for this tumor<br>Macroscopic stage II for which Wertheim type hysterectomy (eligible if stage II grade 3 or stage III at pathology)<br>Prior diagnosis of Crohn's disease or ulcerative colitis<br>Residual macroscopic tumor after surgery<br>Creatinine clearance ≤ 60 ml/min (Cockroft) or ≤ 50 ml/min (EDTA clearance, or measured creatinine clearance)<br>Impaired cardiac function, prohibiting the infusion of large amounts of fluid during cisplatin therapy<br>Peripheral Neuropathy ≥ grade 2<br>Hearing impairment ≥ grade 3, or born deaf
**Number of centres:**	Unlimited; centres can join the ongoing study after authorisation
**Number of patients:**	670
**Planned duration**	Recruitment periods ends Dec 31, 2013

2. INTRODUCTION

2.1 Radiotherapy for intermediate risk endometrial carcinoma

Four randomized trials have established the role of radiotherapy in intermediate risk endometrial carcinoma.[1-4] Conclusions that can be drawn from these data are that pelvic radiotherapy (RT) provides a highly significant improvement of local control, but without a survival advantage. The use of pelvic RT should be limited to those patients at sufficiently high risk of locoregional recurrence (15% or over) to warrant the risk of treatment associated morbidity in order to maximize initial local control and relapse-free survival. For low risk disease (FIGO 1988[1] stages IA and IB grade 1-2), relapse-free survival without further treatment is 95%, and lymphadenectomy and adjuvant RT are not indicated. These patients should be followed after TAH-BSO, and RT can be used as a very effective salvage treatment for the occasional patient with vaginal relapse. For intermediate risk disease (FIGO 1988 stage IB grade 3, IC grade 1 and 2, stage II occult grade 1-2, age 60 or over), omitting RT would leave the patients at a significant risk of vaginal and pelvic relapse. The 10-year locoregional relapse rates in this "high-intermediate" risk group of the PORTEC trial were 4.6% in the RT group and 23.1% in the control group. In the GOG-99 trial, RT resulted in a reduction of 4-year isolated local relapse in the 'high-intermediate risk' group from 13% to 5%. Recent data suggested that vaginal brachytherapy might be used to reduce the risk of vaginal relapse, with less morbidity and better quality of life. The randomized PORTEC-2 trial compared pelvic RT and vaginal brachytherapy for high-intermediate risk disease; first results confirm the hypothesis that vaginal brachytherapy is effective, with less side effects and better quality of life.[5] For high risk disease however, pelvic RT continues to be the most effective adjuvant treatment to ensure pelvic control.

2.2 High-risk endometrial carcinoma

Increasing evidence has accumulated that among stage I endometrial carcinoma patients, the IC grade 3 category should be regarded separately, as this subgroup is at increased risk of pelvic and distant metastases and has lower survival rates[6,7]. During the inclusion period of the PORTEC trial, 99 evaluable patients who had stage 1 endometrial carcinoma, grade 3 with deep myometrial invasion were registered in a separate database and received postoperative radiotherapy. The 5-year actuarial vaginal and pelvic relapse rate of the IC grade 3 patients was 13%, clearly higher than the other stage I patients, who had excellent pelvic control rates after pelvic RT (97-99%)[8]. The 5-year rates of distant metastases were increased in both subgroups with grade 3 tumors: 20% for grade 3 with superficial invasion and 31% for grade 3 with deep myometrial invasion, compared to 3-8% for grade 1 and 2 disease. Overall survival at 5 years was 58% for the IC grade 3 patients, compared to 74% for those with IB grade 3, and 83-86% for IB grade 2 and IC grade 1 and 2 disease ($p<0.001$). In multivariate analyses grade 3 was the most important adverse prognostic factor with hazard ratios for any relapse and for endometrial carcinoma related death of 5.4 ($p=0.0001$) and 5.5 ($p=0.0004$), respectively.

Whether or not surgical staging has been performed, pelvic RT is generally recommended for grade 3 tumors with deep myometrial invasion.[7;9-12] In view of the increased risk of distant relapse and cancer related death, adjuvant chemotherapy is currently being investigated.

[1] For FIGO staging and an overview of FIGO 1988 and 2008 staging criteria, see Appendix B.

Lymph-vascular space invasion has been found to be a major prognostic factor which significantly and independently increases the risk of relapse, especially distant relapse.[3,8,13] In an analysis of 609 stage I-III endometrial cancer patients, those with lymph-vascular space invasion (LVSI) were found to have a 5-year relapse rate of 39%, in contrast to 19% in patients without LVSI ($p<0.0001$). Even in otherwise low-risk stage I disease, the presence of LVSI significantly increased the risk of relapse (28%, versus 14% without LVSI). In stage I patients with high-risk features, those with LVSI had a 43% relapse rate.[13]
Elderly endometrial cancer patients more often have high-risk features and unfavorable histologic types. However, patients and physicians often fear impaired toleration of adjuvant therapy in elderly patients. Elderly patients have been shown to significantly benefit from adjuvant RT and to tolerate RT quite well.[14] Trials should include quality of life analyses to establish both the benefit of more intensive adjuvant treatment, and the costs in terms of added morbidity and influence on quality of life.

2.3 Unfavorable histologic types

Serous and clear cell cancers, up to 10% and 5% of endometrial carcinomas, respectively, have been identified as histologic types with an inferior prognosis due to aggressive growth and spread patterns with frequent diffuse intra-abdominal dissemination. These histologic types often present with advanced disease (46% stage II-IV as compared to 21% for all endometrial cancers). Different treatment approaches (extended surgery, surgery with whole-abdominal radiation, surgery with adjuvant chemotherapy) have been suggested. Recently, several studies have shown that serous and clear cell carcinomas have similar recurrence and survival rates as compared with grade 3 endometrioid carcinomas.[15,16] In a recent analysis of 5694 surgically staged endometrial cancer patients from the 25th annual report of FIGO, 3996 were stage I. Serous and clear cell cancers represented 5.2% of stage I cancers, and grade 3 carcinomas 8.1%. There were more stage I cancers among serous and clear cell cancers than among grade 3 carcinomas (54% and 49% vs. 42%). Five-year survival rates were 72% and 81% for serous and clear cell cancers, compared to 76% for grade 3 disease. Postoperative RT improved 5-year survival with 8% for these histologic types (76% vs. 68% for grade 3; 74% vs. 66% for serous cancers; and 83% vs. 77% for clear cell carcinoma), however these differences were not significant.[16] Preliminary results from an analysis of 68 stage I and II serous cancers suggested that adjuvant treatment with chemotherapy and/or radiotherapy may improve survival.[17] However, recent results from both GOG#122 and the NSGO/EORTC randomized adjuvant chemotherapy trial (see section 2.5) did not show evidence of improved relapse-free or overall survival in the serous histology subgroup. Thus evidence is unclear regarding the benefit of adjuvant chemotherapy in serous or clear cell cancers.

2.4 Surgery

Surgery is the mainstay of treatment of endometrial carcinoma. Surgical evaluation should start with exploration through a midline incision and collection of ascites or peritoneal lavage fluid for cytologic evaluation. Thorough examination and palpation of the pelvic and abdominal organs and lymph node regions should be carried out, and any suspicious sites should be biopsied. Following initial assessment, the standard surgical procedure is a total abdominal hysterectomy with bilateral salpingo-oophorectomy (TAH-BSO).

The role of pelvic and para-aortic lymphadenectomy or lymph node sampling has been widely debated. Determination of nodal involvement has prognostic implications (and is included in FIGO staging), and in patients diagnosed to have node involvement directs further therapy. The potential therapeutic implications of lymphadenectomy are directly related to the risk of nodal disease in the population studied. Prospective and retrospective studies of lymphadenectomy in patients with clinical stage 1-2 endometrial carcinoma without extrauterine spread identified at surgery have shown the rates of pelvic and aortic nodal involvement to be 7-9% and 2-3%, respectively.[18-20] The risk of lymph node involvement varies with the major risk factors, as was demonstrated in the landmark GOG surgical pathologic staging study.[18,22] Patients at high (>15%) risk of microscopic nodal metastases are those with grade 3 and deep invasion and those with advanced disease. Some of these features can be identified at the time of hysterectomy and used to evaluate the indication for lymph node dissection. The addition of lymphadenectomy, especially if both pelvic and aortic lymphadenectomy are performed, prolongs operation time and has side effects such as leg edema (5%), lymphocysts (symptomatic in 5-7%), increased rates of deep vein thrombosis (2%) and small bowel obstruction (up to 5%), increased blood loss and higher transfusion rates (5-10%).[21,23,24] Studies suggesting a survival advantage were small, single center retrospective analyses, flawed by patient selection and stage migration[23-25] Both the larger NCI and Duke University retrospective analyses reported a survival benefit with multiple site lymphadenectomy for grade 3 cancers, while no benefit was found for grade 1-2 disease.[20,21] Lymphadenectomy might therefore be considered for patients with grade 3 disease, cervical involvement, and high-risk histologies.[19-21] It has been shown that if pelvic lymphadenectomy is performed, a minimum of 11 nodes should be removed from multiple sites.[20,21,25]

The results of the MRC-ASTEC trial, the first randomized trial investigating the role of lymphadenectomy in clinical stage I endometrial cancer, have recently been published.[26] 1408 patients were randomized, 704 to TAH-BSO with lymphadenectomy (LN), 704 to TAH-BSO alone. The baseline characteristics were well balanced between the groups. 9% had any nodal involvement. The results showed no benefit of lymphadenectomy: 3-year overall survival rates were 89% (TAH-BSO alone) and 88% (TAH-BSO plus LN), and 3-year recurrence-free survival was even better in the TAH-BSO alone arm (HR 1.35, p=0.017; HR 1.25 after adjustment, p=0.14). Subgroup analysis did not reveal any subgroup benefiting from LN. Analysis of the impact of the number of nodes removed was done by comparing centres with median node counts greater than 10 vs. 10 or less, and greater than 15 vs. 15 or less. In both of these comparisons there was no difference between the arms, with a non-significant trend in recurrence free survival favouring the TAH-BSO alone arm. Lymphoedema was increased in the TAH-BSO plus LN arm, with 8% vs. 1%. A recent Italian randomized trial comparing TAH-BSO with lymphadenectomy (LA) with TAH-BSO alone for stage I endometrial carcinoma confirmed these results, with a median of 30 nodes removed in the LA arm. Although the rate of nodal involvement was 13% in the LA as compared to 3% in the standard arm, rates of disease-free survival, overall survival, relapses and patterns and sites of relapse were the same in both arms.[27]

These results from randomized trials do not support the routine use of lymphadenectomy for endometrial carcinoma patients. The risk of lymph node metastases is known to be increased in high-risk or advanced disease patients, but the risk of other extra-uterine disease is increased accordingly, and treatment schedules which treat microscopic distant disease are required to improve outcome.

2.5 Adjuvant chemotherapy

Most endometrial carcinoma patients treated with chemotherapy had advanced or metastatic disease. Phase II studies have identified doxorubicin, cisplatin, paclitaxel, cyclofosfamide, ifosfamide and carboplatin as active agents, with response rates of 30-35%. Multiagent chemotherapy has been shown to be more effective than single agent therapy. In randomized trials, platinum and/or paclitaxel containing multiagent therapy provided response rates of 34-57% with median remission duration of 9-15 months.[28-30] Doxorubicin and paclitaxel was not found to be superior to doxorubicin and cisplatin (AP) in terms of response (40 and 43%), survival or toxicity.[30] A randomized GOG trial comparing AP therapy with doxorubicin, cisplatin and paclitaxel with filgrastim support (TAP) showed an improved response rate (57% vs. 34%) and a significantly longer median survival of 15 vs. 12 months (58 vs. 50% 1-year survival) for TAP, however at the cost of increased neurotoxicity.[31] Carboplatin and paclitaxel therapy, being the standard for ovarian cancer, has less toxicity than AP or TAP and might be as effective.[32-34] A current GOG trial is comparing carboplatin and paclitaxel with TAP.

Four randomized trials have been conducted which evaluated the efficacy of chemotherapy in the adjuvant setting. The oldest trial, using single agent doxorubicin, did not show any benefit.[35] The results of GOG #122, a randomized trial comparing whole abdominal radiotherapy (WAI) with 8 cycles combination doxorubicin-cisplatin chemotherapy (AP) in advanced (stages III-IV, residual tumor up to 2 cm allowed) endometrial carcinoma, have shown combination chemotherapy to improve both progression-free and overall survival rates, with a difference in disease-free survival of 12% at 5 years (50% vs. 38%), and in overall 5-year survival of 11% (53% vs. 42%).[36] However, recurrences remained frequent with 13%, 16% and 22% recurrence, respectively, in the pelvis, abdomen and extra-abdominal sites or liver for WAI, and 18%, 14% and 18% for AP chemotherapy. The subgroup of serous cancers (20% in both groups, n=83) did not benefit from adjuvant chemotherapy. In subgroup analysis the hazard ratio for death after AP chemotherapy vs WAI was 1.02 for serous cancers, in contrast to 0.48 for endometroid cancers. Adverse effects were substantial, especially in the AP arm.

The Japanese multicenter randomized JGOG 2033 trial compared whole pelvic irradiation (WPI, at least 40 Gy) with 3 or more cycles of cyclophosphamide, doxorubicine and cisplatin (CAP) chemotherapy in 385 evaluable patients with stage IC-IIIC endometrioid adenocarcinoma ("intermediate risk"; 60% stage IC, median age 59; 15% grade 3). At a median follow up of 5 years, no differences in progression-free survival (WPI 83.5% vs. CAP 81.8%) and overall survival (85.3% vs. 86.7%) were seen. Relapse rates were similar: 15.5% vs. 17.2% of the patients had a relapse, with 6.7% and 7.3% pelvic and 13.5% and 16.1% extrapelvic relapse, respectively. In a subgroup of "high to intermediate risk" cases (stage IC >70 yrs, IC grade 3, stage II or stage IIIA(cytology), n=120) a survival benefit for CAP was suggested, while no PFS or OS difference was found among 75 "high risk" cases (stage IIIA-IIIC). Grade 3-4 toxicity rates were 1.6% (WPI) and 4.7% (CAP), p=0.08.[37]

An Italian randomized trial with a similar design as the JGOG trial, comparing whole pelvic irradiation (WPI 45-50 Gy) with 5 cycles of cyclophosphamide, doxorubicine and cisplatin (CAP) chemotherapy, included 345 evaluable patients with stage IC-IIIC endometrioid adenocarcinoma.[38] In this trial the majority of patients were stage III (64% stage III, 36% stage IC-II grade 3). After a median follow-up of 95.5 months there were no significant differences in progression-free and overall survival rates, with 5-year OS rates of 69 (RT) vs.

66% (CAP) and 5-year PFS rates of 63 vs. 63%. Radiotherapy delayed pelvic relapses and chemotherapy delayed metastases (both non-significant trends), but the trial did not show any survival difference.

Increased pelvic relapse rates have been reported when using adjuvant chemotherapy alone in patients with high-risk or advanced stage endometrial carcinoma.[39] Of the 67% who relapsed, 40% had pelvic recurrence and 56% distant relapse. The 3-year pelvic failure rate was 47%, and in 31% the pelvis was the first or only site of recurrence. As these data support the use of pelvic radiotherapy in high-risk patients undergoing adjuvant chemotherapy, future trials should explore the optimal sequencing of therapy and the use of combined radiotherapy and chemotherapy.

2.6 Combined radiotherapy and chemotherapy

First pilot studies have indicated concurrent radiotherapy and chemotherapy (chemoradiation) to be tolerable.[40,41] The RTOG have reported a phase II trial of concurrent pelvic radiotherapy (45 Gy in 1.8 Gy fractions and a brachytherapy boost) and cisplatin (2 courses of 50 mg/m^2 on days 1 and 28), followed by 4 courses of cisplatin and paclitaxel after RT for high risk or advanced stage endometrial carcinoma.[42] A total of 46 patients were included; 44 were evaluable, of whom 15 had stage I-II disease with high-risk features, and 29 (66%) had stage III disease. Surgery consisted of TAH-BSO with or without additional surgical staging. After completion of RT, patients received 4 additional courses of cisplatin (50 mg/m^2) and paclitaxel (175 mg/m^2 as a 24-h infusion) at 28-day intervals. The protocol completion rate was 98%. During RT and Cisplatin, 2 patients had their second cycle held for 1 week and 1 patient for 2 weeks because of low blood counts and diarrhea. During the adjuvant phase, 35 patients received all 4 cycles, 5 patients received 3 cycles, 2 patients received 2 cycles and 2 did not receive any chemotherapy. Acute toxicities during chemoradiation were grade 1 in 27%, grade 2 in 43%, grade 3 in 27% and grade 4 in 2% (grade 3-4 toxicity: 8 hematologic, 4 nausea/vomiting, 3 other GI). During adjuvant chemotherapy, 21% grade 3 and 62% grade 4 toxicity was observed, severe toxicity being primarily hematologic (25 of 26 grade 4). The high rates of grade 4 hematological toxicity were almost certainly related to the schedule of paclitaxel administration (24 hour infusion). Chronic toxicity was grade 1 in 16%, grade 2 in 41%, grade 3 in 16%, and grade 4 in 5%, which included 1 patient with a small bowel complication. Four-year disease-free and overall survival rates were 81 and 85% for the whole group, and 72 and 77% for stage III patients. The 4-year rates of pelvic, regional, and distant recurrence were 2%, 2%, and 19% respectively. There were no recurrences among stage IC and II patients, suggesting additive effects of chemotherapy and radiation. These are encouraging results for further trials in this field. The rate of treatment completion, the toxicity profile and efficacy of this treatment schedule enable its use in a multicenter trial.

At the American Society of Clinical Oncology 2007 Annual Meeting, Hogberg *et al* presented the results of the NSGO-EC-9501/EORTC55991 trial of radiation alone *versus* adjuvant chemotherapy before or after radiation in 382 patients with stage I, II, IIIA (positive peritoneal cytology only), or IIIC disease who had high risk factors for recurrence (one or more of: grade 3 tumor, deep myometrial invasion, non-diploid DNA or serous/clear cell/anaplastic histology).[43] Chemotherapy was not standardized and could be AP, TAP, paclitaxel + platinum (TP or TC) or paclitaxel, cisplatin, plus epirubicin (TEC). Results showed an improvement in progression free survival (PFS) in the chemotherapy arm (7% improvement at 5 years, 79 vs 72%, *p*=0.03), and a non-significant 8% increase in overall survival (82 vs 74% *p*=0.08). Interestingly, the

serous/clear cell subset, although relatively small numbers (n= 78 total) did not show evidence of benefit from adjuvant chemotherapy on PFS. Weaknesses with this trial were the fact that a variety of regimens were used, some with and without taxane or anthracycline, the timing of radiation in the experimental arm was variable and it was underpowered to the plausible survival benefit of adjuvant chemotherapy. Taken together, these data reinforce the need for an adequately powered trial with a single adjuvant chemotherapy treatment in endometrial cancer.

A phase III randomized trial, comparing chemoradiation and adjuvant chemotherapy with pelvic radiation in patients with high-risk endometrial cancer will establish the role of concurrent chemoradiation and adjuvant chemotherapy and thus be an important contribution to patient tailored treatment.

3. Trial objectives

The primary objective of this study is to establish overall survival and failure-free survival of patients with high-risk and advanced stage endometrial carcinoma treated after surgery with concurrent radiotherapy and chemotherapy, followed by adjuvant chemotherapy, in comparison with patients treated with pelvic radiation alone. Failure is defined as relapse or death due to endometrial carcinoma or due to treatment complications. Primary endpoint is overall survival; second primary endpoint is failure-free survival.

Secondary objectives are to establish and compare the rates of pelvic and distant recurrence, severe (grades 3 and 4) treatment-related toxicity, and quality of life.

4. Trial design

In this international multicenter trial, 500 (to 670) patients with either FIGO stage I or 2 endometrial adenocarcinoma with high-risk features or stage 3 endometrial carcinoma who meet the inclusion criteria will be randomised (1:1) to one of the following arms:

1. external beam pelvic radiotherapy (control arm)
2. concurrent radiotherapy and chemotherapy followed by chemotherapy (experimental arm)

Primary study endpoints will be 5-year actuarial overall survival, and 5-year actuarial failure-free survival (FFS). Failure is defined as relapse, or death due to endometrial carcinoma or due to treatment complications.

Secondary endpoints will be quality of life, severe treatment related morbidity, rates of vaginal/pelvic relapse and distant metastases.

Statistical considerations are found in Section 12.

Stratification will be done for:

1. participating group (DutchGOG vs. UK NCRI vs. NCIC CTG vs. ANZGOG vs. Italian ManGO group vs UNICANCER)
2. mode of surgery (TAH-BSO vs. TAH-BSO plus lymphadenectomy vs. laparoscopic procedure (LAVH or TLH-BSO) vs. laparoscopic procedure plus lymphadenectomy)
3. stage (FIGO 2009 IA vs. IB vs. II vs III; FIGO 1988 IB vs. IC vs. II vs. III)
4. histological type (endometrioid carcinoma vs. serous or clear cell carcinoma)

5. Patient selection

5.1 Inclusion criteria

To be eligible for this trial, patients will need to meet all of the following inclusion criteria:

1. Histologically confirmed endometrial carcinoma, grade of differentiation determined according to the FIGO/AFIP criteria, with one of the following postoperative FIGO 2009 stages[45] (Appendix B); confirmed at pathology review (see section 8.1):
   a. Stage IA with myometrial invasion, grade 3 with documented lymph-vascular space invasion (LVSI)
   b. Stage IB grade 3
   c. Stage II
   d. Stage IIIA or IIIC; or IIIB if parametrial invasion
   e. Stage IA with myometrial invasion, IB, II or IIIA/C with serous or clear cell histology

   During the initial years of the trial, FIGO 1988 staging was used (see Appendix B). Criteria using FIGO 1988 staging:
   a. Stage IB grade 3 with documented lymph-vascular space invasion (LVSI)
   b. Stage IC or IIA grade 3
   c. Stage IIB
   d. Stage IIIA* or IIIC **IIIA based on peritoneal cytology alone is only eligible if grade 3*
   e. Stage IB, IC, II or III with serous or clear cell histology
2. Recommended surgery is TAH-BSO (total abdominal hysterectomy and bilateral salpingo-oophorectomy). However, for a patient who has had laparoscopic surgery, and/or lymphadenectomy and/or full surgical staging, and was found after pathology diagnosis to meet the eligibility criteria, inclusion in the trial is permitted.
3. WHO-performance status 0-2 (Appendix C)
4. WBC ≥ 3.0 x 10^9/L.
5. Platelets ≥ 100 x 10^9/L.
6. Bilirubin ≤ 1.5 x UNL
7. ASAT/ALAT ≤ 2.5 x UNL
8. Written informed consent

5.2 Exclusion criteria

The following criteria exclude the patient from enrolment in this trial:

1. Uterine sarcoma (including carcinosarcoma)
2. History of any previous malignancy, except for non-melanomatous skin cancer, within the last 10 years (and if previous malignancy > 10 years ago there should be no evidence of recurrence)
3. Previous pelvic radiotherapy
4. Hormonal therapy or chemotherapy for this tumor
5. Macroscopic gross cervical involvement for which radical (Wertheim type) hysterectomy has been performed (however, if at pathology diagnosis stage II grade 3 or stage III, the patient is eligible)
6. Prior diagnosis of Crohn’s disease or ulcerative colitis
7. Residual macroscopic tumor after surgery

8. Impaired renal function: creatinine clearance ≤ 60 ml/min (calculated according to Cockroft) or ≤ 50 ml/min (EDTA clearance, or measured creatinine clearance)
9. Impaired cardiac function, which in the assessment of the investigator would prohibit the infusion of large amounts of fluid during cisplatin therapy
10. Peripheral Neuropathy ≥ grade 2
11. Hearing impairment ≥ grade 3, or born deaf

6. Summary of treatment schedule (see also Appendix A)

Patients in the standard arm will be treated with pelvic RT (48.6 Gy in 1.8 Gy daily fractions, 5x per week), and a brachytherapy boost in case of cervical involvement.

Patients in the experimental arm will be treated with the same RT schedule (including brachytherapy in case of cervical involvement), and 2 cycles of cisplatin 50 mg/m^2 given concurrently with RT at a 21-day interval. The 1st concurrent cycle is to be given within days 1-3 of RT, and the 2nd cycle 3 weeks after the 1st cycle (i.e. within days 22-24, in the 4th week of RT, 16th-18th RT fraction days). After completion of RT, patients will receive 4 additional cycles of adjuvant chemotherapy: carboplatin AUC 5 and paclitaxel 175 mg/m^2 (over 3 hours) at 21-day intervals.

Toxicity will be evaluated before treatment (baseline), at completion of radiotherapy, at each chemotherapy cycle and at each follow-up. Quality of life will be evaluated before treatment (baseline), at completion of radiotherapy, and at 6, 12, 18, 24, 36 and 60 months from the date of randomisation.

7. Staging and Treatment

7.1 Staging

Pre-operative and/or postoperative staging procedures (see also the checklist in Appendix J):

1. Medical history, physical and complete pelvic examination
2. Endometrial biopsy and/or endometrial curettage
3. Blood count and chemistry tests
4. Creatinine clearance (Cockroft, or measured creatinine clearance or EDTA clearance)
5. Ca-125 (preferably pre-and postoperatively)
6. Chest radiography or chest CT scan
7. Abdominal and pelvic CT-scan or MRI-scan: to exclude extra-uterine disease and especially para-aortic lymphadenopathy (preferably pre-operatively, if not done, at least postoperatively). Suspicious nodes or other localisations should be biopsied. Non-specific post-surgical changes can be accepted.

After surgery and pathology, the FIGO stage should be assigned on the basis of the surgical and histological findings (Appendix B).

7.2 Surgery

The surgical procedure should start with obtaining peritoneal fluid samples for cytology taken from the pelvis and abdomen. Immediately upon entry in the peritoneal cavity, fluid samples are to be obtained, preferably by aspiration of free fluid, or alternatively by instilling and aspirating 100 cc of normal saline solution. This is followed by a thorough exploration of the intra-abdominal contents. The omentum, liver, peritoneal cul-de-sac

and adnexal surfaces should be examined and palpated for any possible metastases, followed by careful palpation for suspicious or enlarged nodes in the aortic and pelvic nodal areas. The uterus should be thoroughly evaluated for any breach in the serosa, and the distal ends of the fallopian tubes should be closed (eg. by placing clamps across the tube and utero-ovarian ligament).
At laparotomy the abdomen should be opened with a vertical midline abdominal incision. The standard surgical procedure is extrafascial total hysterectomy with bilateral salpingo-oöphorectomy (TAH-BSO) and histological verification of any suspected metastases. Laparoscopic procedures (laparoscopically assisted vaginal hysterectomy or total laparoscopic hysterectomy) are permitted if the centre uses these as routine procedure after substantial experience, completion of learning curve and clinical evaluation). Thorough laparoscopic inspection of the entire abdominal should be done, and a laparoscopic procedure should be converted to an open procedure if extra-uterine spread or metastases are identified.
Lymph node debulking and para-aortic lymph node sampling are recommended in case of macroscopic positive pelvic nodes and/or para-aortic nodes. Other extra-uterine tumor deposits should be completely removed. For serous or clear cell carcinoma, full staging as in ovarian cancer (with omentectomy, peritoneal biopsies and lymph node sampling) is strongly recommended.
At the completion of the operation there should be no remaining macroscopic tumor.

7.3 Radiotherapy

7.3.1 External beam pelvic radiation

Target Volume:
The clinical target volume (CTV) consists of the proximal 1/2 of the vagina, the parametrial tissues, and the internal, external and distal common iliac lymph node regions up to the upper S1 level.
In case of external or internal iliac lymph node involvement the common iliac lymph node regions are to be included up to the aortic bifurcation. In case of common iliac node involvement the aortic bifurcation should be included up to the lower peri-aortic region (margin of at least 2 cm above the highest lymph node region involved). In case of peri-aortic involvement, the peri-aortic lymph node region should be included up to the higher para-aortic region (margin of at least 2 cm above the highest lymph node region involved).
If a complete bilateral lymphadenectomy has been performed with at least 12 lymph nodes (from all sites: left and right external, internal and common iliac regions) and all lymphnodes are free of tumor at histopathologic evaluation, the upper border of the CTV is at the iliac bifurcation.
The Planning Target Volume (PTV) consists of the CTV with a 7-10 mm margin. A detailed description and guidelines for delineation of the CTV are provided in Appendix E.

Positioning and verification:
The choice of the supine or prone position is left to the treating physician. In the case of prone positioning, the use of a belly-board is recommended. Treatment with a full bladder is advisable. A CT scan for simulation and planning is mandatory. The positioning of the patient during simulation and treatment should be reproduced with the aid of orthogonal laser beams. The positioning should be verified by electronic portal images or megavolt films at least twice, at the beginning of treatment and after one week. Use of a verification and correction procedure is recommended.

Dose and fractionation:
48.6 Gy, at 1.8 Gy per fraction, specified at the isocenter, 5 fractions a week. This dose is equivalent to 46.6 Gy in 2 Gy fractions for an α/β ratio of 3 (late effects), and to 47.8 Gy in 2 Gy fractions for α/β 10 (acute effects). For UK centers, a dose range of 45 to 50.4 Gy at 1.8 Gy fractions is allowed, with a recommended dose of 48.6 Gy.
Treatment should preferably be started within 4-6 weeks after surgery, but no later than 8 weeks after surgery. Treatment breaks should be avoided, and overall treatment time should be kept within 6 weeks. Treatment breaks due to public holidays and machine maintenance should not exceed 2 days.

Technique:
A planned volume (four-field 'box', 3-field or multiple field techniques with or without supplementary fields or segments) will be employed, with individual shielding in all fields. The use of CT-scan based three-dimensional treatment planning is mandatory. The dose is be specified at the ICRU reference point. Homogeneity requirements should be according to the ICRU-50 recommendations. IMRT is allowed if a center has completed clinical introduction of standard IMRT for pelvic fields and adequate QA procedures are employed. Permission for use of IMRT will be granted on an individual basis after consultation with the national group study coordinator.
Patients should be treated with megavolt photons from a linear accelerator, using at least 6 MV photons, but treatment with higher energies (10 MV or higher) is recommended.
A dummy run or quality control survey of treated patients will be done during the course of the trial.

7.3.2 Vaginal brachytherapy

A brachytherapy boost is to be delivered to patients with documented cervical involvement. Brachytherapy should be either incorporated within the last 1-2 weeks of EBRT (not giving both on the same day), or be given in the first week after completion of EBRT (HDR sessions should be given at least 3 days apart). Overall treatment time for radiotherapy (EBRT and brachytherapy) should not exceed 50 days.
Brachytherapy is either given with ovoids or with a vaginal cylinder, active length 2-3 cm, with the reference isodose covering at least the proximal 3 cm of the vagina. High-dose-rate (HDR), low-dose-rate (LDR) and pulse-dose-rate (PDR) schedules are permitted, which deliver an equivalent dose of 14 Gy at 5 mm from the vaginal mucosa (to obtain a cumulative EDQ2 of 60 Gy at 5 mm). Examples of such schedules: HDR 10 Gy in 2 fractions, at least 3 days apart; 8.5 Gy in one fraction, or LDR 14 Gy in one session.
The dose should for ovoids be specified at 5 mm cranial from the center of the ovoid surfaces, and for a vaginal cylinder at 5 mm from the surface of the cylinder, in the central plane (i.e. the plane perpendicular to the cylinder axis), and at 5 mm from the vaginal top on the axis of the cylinder.
Simulation radiographs are taken in the anterior and lateral directions, or alternatively CT- or MRI- planning may be used. Dose distributions should be obtained, and the dose in the bladder and rectum reference points should be computed (according to ICRU-38). For CT- or MRI-based planning the cumulative (external beam and brachytherapy) EDQ2 dose in a 2cc volume of the rectum and bladder should not exceed 75 Gy and 90 Gy, respectively.

7.3.3. Radiotherapy quality assurance

Radiotherapy quality assurance will be conducted by independent review of radiotherapy plans by the Trans Tasman Radiation Oncology Group QA office (TROG protocol), see Appendix E. Radiotherapy treatment planning (RTP) electronic data files will for this purpose be exported – without any patient identifiers, only using the PORTEC-3 trial code - in DICOM RT or RTOG format for review. All Radiation Oncology centers participating in this study will be required to submit at least one recent case for retrospective review, and further prospective audits will be undertaken of 1 in every 5 cases or at least once a year for each centre from 2012 onwards.

7.4 Chemotherapy

Cisplatin, Paclitaxel and Carboplatin should be given in the doses and schedule detailed below, according to standard practice in the participating country. Suggested pre- and post chemotherapy medication and hydration guidelines are detailed below. Please refer to the Group Specific Appendix (or Chemotherapy Regimen Appendix) for country-specific guidelines for chemotherapy administration and hypersensitivity management. For detailed information on each drug, please refer to the relevant Summary of Product Characteristics.

7.4.1. Concurrent phase: Cisplatin

Agent	Dose/day	Route	Days
Cisplatin	50 mg/m^2 in 1000 ml NaCl 0.9% or 2.5-3%*	I.V., in 2 hours	1, 22

* according to local standard protocol

Suggested schedule for pre- and posthydration: Prehydration: 1000 ml NaCl 0.9% in 2 hours.

Posthydration: 2000 ml NaCl 0.9% in 4 hours, with adequate i.v. suppletion of K and Mg.

Other schedules (if used in standard protocols) may be chosen at the participating centre's discretion, however, a minimum total hydration volume of 1500 ml should be used.

The first concurrent cycle of cisplatin should be given within days 1-3 of RT, and the second cycle 3 weeks after the first cycle (4th week of RT, within days 22-24, 16th-18th RT fractions).

Dose modifications of cisplatin

Toxicity	Adjustment	Remarks
**Hematologic toxicity**		
ANC < 1.5 x 109/L	Postpone 1 week	If recovery requires > 1 week stop cisplatin
Platelets < 100 x 109/L	Postpone 1 week	If recovery requires > 1 week stop cisplatin
**Renal toxicity**		
GFR < 50 ml/min (Cockroft)<br>GFR < 40 ml/min (measured creatinine or EDTA clearance)	Postpone 1 week	If recovery requires > 1 week stop cisplatin<br>If GFR < 40 ml/min (Cockroft) or remains < 40 ml/min (measured): stop cisplatin
**Neurologic toxicity**		
Neuropathy $\geq$ grade 2	Stop cisplatin	
**Other toxicities**		
Other toxicity > grade 2	Postpone 1 week	If recovery requires > 1 week stop cisplatin

7.4.2. Adjuvant phase

Carboplatin and paclitaxel

Agent	Dose/day	Route	Days
Paclitaxel	175 mg/m^2	I.V., in 3 hours	1, 22, 43, 64
Carboplatin	AUC 5 (calculated AUC)	I.V., in 1 hour	1, 22, 43, 64

Four cycles of carboplatin and paclitaxel will be given, at 3 week intervals.

Adjuvant chemotherapy should be started within 3 weeks after termination of radiotherapy, and preferably 3-4 weeks after the last administration of cisplatin.

Before starting adjuvant chemotherapy, the toxicity of the concomitant chemo-radiotherapy should be resolved to less than grade 2.

AUC for carboplatin should in principle be recalculated at each cycle, but should at least be recalculated in case of increasing serum creatinin (increase of 10% and/or out of normal range) and/or weight changes.

Dose modifications of carboplatin and paclitaxel

**Toxicity**	**Adjustment**	**Remarks**
**Hematologic toxicity**		
ANC < 1.5 x 10^9/L	Postpone 1 week	If after 1 week still <1.5 but >1.0: resume chemotherapy and add G-CSF; if no G-CSF available: postpone second week. If after 2nd week ≥1.5 resume at dose level carboplatin AUC 4, with full dose paclitaxel. If after 2nd week still <1.5 postopone 3rd week.<br>If ANC does not resolve to ≥1.5 by 3rd week; or if second occurrence (with dose reduction) <1.5 not resolving after 2 weeks: discontinue carboplatin and continue with single agent paclitaxel.
Platelets < 100 x 10^9/L	Postpone 1 week	If no recovery to >100 postpone 2nd week<br>If platelets >100 after 2nd week resume at dose level carboplatin AUC 4, with full dose paclitaxel<br>If platelets do not resolve to >100 after 3 weeks, or at second occurrence of platelets <100 not resolving after 1 week discontinue carboplatin and continue with single agent paclitaxel.
**Neurologic toxicity**		
Neuropathy grade 2	Postpone 1 week<br>Postpone second week if still grade 2	If recovery to grade 1 or less: dose reduction for paclitaxel to 135 mg/m^2 for subsequent cycles<br>If no recovery to grade 1 after 2 weeks:<br>Stop paclitaxel and continue carboplatin AUC 6
Neuropathy > grade 2	Stop paclitaxel	Continue carboplatin with AUC 6<br>(higher dose for single agent carboplatin)
**Other toxicities**		
non-hematologic and non-neurologic toxicity > grade 2	Postpone 1 week	If recovery requires > 1 week postpone until recovery of toxicity to grade 1<br>If no recovery to grade 1 after 3 weeks discontinue carboplatin and paclitaxel

In case of severe hypersensitivity to paclitaxel, where rechallenge is not medically indicated **or** if repeated severe reaction at rechallenge, paclitaxel should be substituted by docetaxel 75 mg/m^2. In case of hypersensitivity to docetaxel as well, continue with carboplatin AUC 6
In case of severe hypersensitivity to carboplatin, where rechallenge is not medically indicated **or** if repeated severe reaction at rechallenge, carboplatin should be substituted by cisplatin 50 mg/m^2.
In case of fever with grade 3 or 4 neutropenia, consider use of G-CSF (see Supportive Care), or prophylactic antibiotics at subsequent cycles.
For a checklist of investigations before and during chemotherapy, see Appendix J.

7.4.3. Supportive care

Concomitant chemo-radiotherapy:
Anti-emetic therapy before start of cisplatin: aprepitant 125 mg, dexamethason 12 mg, ondansetron 8 mg; or use a standard combination of a corticosteroid and a 5HT-antagonist (the use of aprepitant is at discretion of the participating centre).
After administration of chemotherapy anti-emetic therapy is at discretion of the participating centre.

Adjuvant chemotherapy:
Premedication before start of paclitaxel: dexamethason 20 mg i.v., clemastine 2 mg i.v. and ranitidine 50 mg i.v. or a similar schedule at the discretion of the participating centre**.**
Anti-emetic therapy: 5HT-antagonist (the use of aprepitant is at discretion of the participating centre).
After administration of chemotherapy anti-emetic therapy is at discretion of the participating centre.
Use of G-CSF is permitted for secondary prophylaxis after a neutropenic complication according to the American Society of Clinical Oncology guideline (2006 Update of ASCO Recommendations for the Use of White Blood Cell Growth Factors: An Evidence-Based Clinical Practice Guideline). [44]
Hypersensitivity reaction to paclitaxel or carboplatin:
Stop infusion immediately and administer 10 mg dexamethason i.v., clemastine 2 mg i.v., epinephrine 1 mg.
After recovery of symptoms, infusion may be rechallenged:
0-15 min: paclitaxel or carboplatin flow 15 ml/hour.
15-30 min: NaCl 0,9% + 2 mg clemastine,
30-45 min: paclitaxel or carboplatin flow 84 ml/hour
45-90 min: If no reaction occurs, paclitaxel flow 170 ml/hour or carboplatin flow 500 ml/hour

8. Pathology

8.1. Histopathologic evaluation

The diagnosis of the regional pathologist will be first indication of eligibility for the trial. However, given the considerable number of discordances, with 8% discrepancies altering patient management[46], the specimens should be reviewed by one of the reference pathologists and their diagnosis will determine final eligibility and entry in the study. Immediately at the oncology board or consultation of the gynaecologist with the radiation oncologist at which eligibility is considered, the pathologist should be requested to send the histopathologic slides and a copy of the pathology report for review to one of the reference pathologists (section 8.2). The

reference pathologist will send final diagnosis within one week, after which eligibility can be determined and the patient can be informed about the study.

A standardized evaluation of the specimens according to international criteria is important to obtain information on the pathologic prognostic factors. It should be documented at which parts of the uterus the samples are obtained. The following samples should be obtained in all cases: a representative sample of the deepest myometrial invasion at a plane perpendicular to the serosal surface; a transversal section through the lower uterine segment just proximal to the endocervix; a longitudinal section through the lower uterine segment and endocervix, sections through both cornuae; and representative sections of the tumor.

Macroscopic evaluation should include:

- size and aspect of the uterus and adnexa, status of the serosal surface
- location of the tumor in the uterus
- size of the tumor (maximal diameter and thickness)
- invasion to < 50% or ≥ 50% of the myometrial width
- minimal distance (in mm) between the tumor and the serosa at the point of the deepest myometrial invasion
- width of the uninvolved myometrium
- involvement of the lower uterine segment and of the endocervix
- involvement of the cornuae and of the fallopian tubes
- involvement of the ovaries
- size and number of lymph nodes if removed at surgery
- involvement of the omentum and any other tissue or biopsy obtained at surgery

Microscopic evaluation should include:

- histologic classification according to the International Society of Gynecologic Pathologists (Appendix D). For mixed endometrioid and serous or clear cell cancers, the percentage of each component should be recorded. Histologic type is serous or clear cell, respectively, if the proportion of this component is at least 25% (see Appendix D)
- histologic grade according to the FIGO 1988 criteria (Appendix D)
- invasion to < 50% or ≥ 50% of the myometrial width
- minimal distance (in mm) between the tumor and the serosa at the point of the deepest myometrial invasion
- involvement of the mucosa in the cornuae
- involvement of the lower uterine segment
- involvement of the endocervical glands and/or the cervical stroma
- presence or absence of lymph-vascular invasion (LVSI)
- involvement of the ovaries
- number of involved and uninvolved lymph nodes (if present)
- involvement of the omentum and other tissues and biopsies (if present)
- presence or absence of malignant cells in the peritoneal fluid

Definition of LVSI: morphological vital tumor emboli in endothelial lined lumina containing erythrocytes and/or lymphocytes outside the tumor mass. Lumina following the outer contour of tumor fragments are to be considered shrinkage artefacts. LVSI is a microscopic diagnosis (no immunohistochemistry).

8.2. Central pathology review

Central pathology review will be conducted by the reference gynaecologic pathologists (as appointed by each participating group) to determine eligibility of a patient for the study. In addition to the review of the histopathologic diagnosis and grading, the review will focus on the clinical significance and reproducibility of tumor grading, depth and pattern of myometrial invasion, lymph-vascular space invasion, and the comparison of conventional and new prognostic factors.

For translational research, fresh frozen tumor samples are preferable. Participating centers with adequate in-house pathology facilities will be requested to participate in the collection of frozen tumor samples. Otherwise, paraffin-embedded blocks will be used.

After slide review, determination of eligibility and patient consent, the regional pathologist will be requested to send a separate sample of the tumor for the study purposes. These tumor blocks will be saved in a dedicated tissue bank for translational research (see 8.3).

Reference pathologists for The Netherlands:

Dr V.T. Smit (subsitute: Prof. Dr G.J.Fleuren)
Pathology Department,
Leiden University Medical Centre
Albinusdreef 2, Postbus 9600
2300 RC Leiden
Tel: +31 (0)71 526 6628 / 5269111 page 9964
Fax: +31 (0)71 524 8158
Email: V.T.H.B.M.Smit@lumc.nl

Dr H. Hollema (subsitute: Dr J. Bart)
Pathology Department
University Medical Centre Groningen
Hanzeplein 1, Postbus 30001
9700 RB Groningen
Tel: +31 (0)50 361 4684
Fax: +31 (0)50 361 2510
Email: h.hollema@path.umcg.nl

8.3 Translational research

Paraffin embedded tissue blocks (1 cm^2) will be collected from all consenting patients and archived for translational research. Details regarding submission of tissue/serum samples will be found in Group Specific appendices. In addition, a subset of centres with adequate in-house pathology facilities will cooperate in saving snap frozen tissue samples (1 cm^2) and serum samples from before and 3 months after treatment. All tissue samples from Dutch patients will be stored in the central pathology centres (LUMC, Leiden and UMC Groningen), coded by study number, until analysis.

A tissue micro array will be constructed from these samples to perform high throughput analysis, next to standard techniques like immunohistochemistry on whole paraffin slides and fresh frozen tissue. Proteomics will be performed on a selected set of serum samples.

The focus of research will be to determine the value of newly identified prognostic markers as compared to known prognostic markers in our study population. The aim of these studies will be to define prognostic markers which discriminate between patients who may benefit from systemic treatment, in other words to further individualise treatment schedules. The second goal of translational research will be to define new molecular targets for adjuvant treatment. The combination of a high number of patients with a less common

histologic subtype and the availability of modern techniques give us the unique opportunity to make true progress in the treatment of high-risk endometrial cancer patients.

9. Follow-up, toxicity evaluation and adverse events

9.1. Follow-up

At the completion of radiotherapy, an end-of-radiotherapy follow up visit after 2-4 weeks should be planned by the radiation oncologist to assess the acute toxicity. If toxicity CTC grade $\geq$ 2 has occurred during or after radiotherapy, the Toxicity Form (Form 6) should be sent in. A Quality of Life Questionnaire with a pre-stamped return envelope should be handed out to the patient. For the patients randomized to radiation alone, the subsequent Quality of life Questionnaires will be sent directly to their home address (if permission has been given).

Patients randomized to combined therapy will also be evaluated and assessed for toxicity by the medical oncologist during the period of concomitant chemo-radiotherapy and during each cycle of adjuvant chemotherapy, until 2-4 weeks after the last cycle. If toxicity CTC grade $\geq$ 2 has occurred during or after any cycle, a Toxicity Form (Form 6) should be sent in. Quality of life assessment will be done at 6 months after randomization in both arms, which is shortly after completion of chemotherapy in the combined arm.

After completion of adjuvant chemotherapy, the patients will be evaluated during alternating follow-up visits to their gynecologist and radiation oncologist. Patients in the chemotherapy arm will also be evaluated by their medical oncologist, at least during the first year. Patients will be assessed every 3 months for the first 2 years, and every 6 months up to 5 years. Long-term outcome evaluation at 7 and 10 years should be obtained, preferably by follow-up visits, or at least by General Practitioner enquiry. Follow-up CRF are required at 6-month intervals from the date of randomization during the first 5 years and at year 7 and 10 and at each trial event (see Appendix I).

At each follow-up visit, a history is to be obtained with special emphasis on treatment related morbidity, and a physical and pelvic examination will be done. The patient's performance status and body weight should be recorded. A chest radiograph and blood count and chemistry tests (including Ca-125) will be obtained once a year, up to the 5th year. Other procedures, such as vaginal smears or biopsies and CT- or MRI-scans are to be done only on indication. A checklist is provided in appendix H.

After the diagnosis of any relapse treatment information is required (Recurrence form) and follow-up continues according to protocol guidelines. In case of distant metastases or locoregional relapse not amenable to treatment with curative intent, QoL questionnaires can be discontinued.

9.2. Reasons for going off protocol treatment

If a patient is going off protocol treatment, the reason should be documented on the CRF according to the following listing:

- normal treatment completion
- progressive disease / death to disease progression
- adverse event / toxic death
- intercurrent disease / death
- refusal or other reasons

9.3. Adverse events and reporting of adverse events

9.3.1. Definitions

An **adverse event** (AE) is any symptom, sign, illness or experience, which develops or worsens in severity from informed consent to up to 30 days following the last administration of any of the study treatment. Intercurrent illnesses or injuries should be regarded as adverse events.

Adverse events are classified as either serious or non-serious.

A **serious adverse event** (SAE) is any adverse event that is:

- fatal
- life-threatening
- requires or prolongs hospitalisation
- results in persistent or significant disability or incapacity
- a congenital anomaly or birth defect
- an important medical event.

Important medical events are those which may not be immediately life threatening, but are clearly of major clinical significance. They may jeopardise the patient, and may require intervention to prevent one of the other serious outcomes.

9.3.2. Adverse events (AE)

Special attention should be paid to the occurrence of adverse events (AE) throughout the study period. During the concurrent phase, patients should be evaluated by both the radiation and the medical oncologist for radiation and chemotherapy toxicities. During the adjuvant phase, patients are seen by the medical oncologist. All observed toxicities should be graded according to the NCI-Common Terminology Criteria for Adverse Events (NCI-CTCAE version 3.0, Appendix F) and documented on the Toxicity Form (Form 6). The clinical course of each event should be followed until resolution, stabilisation or until it has been determined that study treatment or participation is not the cause.

9.3.3. Serious Adverse Events (SAE) and Suspected Unexpected Serious Adverse Reactions (SUSAR)

All SAE occurring during the treatment period and within 30 days thereafter, whether or not considered to be related to the study treatment, must be reported **within 24 hours** by fax to the central data management office (Netherlands: IKW Trial Office, Leiden, fax +31 71 526 6712; participating countries: to the Country Coordinating Centre and copy to the IKW Trial Office), using the completed SAE form, and thereafter documented in detail, as indicated on the SAE form. Information is required as to the date and time of onset, duration, AE-term and peak intensity (according to the NCI-CTCAE version 3.0), and outcome of the adverse event (recovered completely; residual effects; continuing). The investigator should classify the relationship of a SAE to the treatment (none; unlikely; possible; probable; definite). The investigator must respond to queries and requests for additional information **within 24 h**.

LUMC, the Sponsor (“verrichter” in the terminology of Dutch law) is responsible for SAE assessment and reporting to the authorities in accordance with all requirements of the Dutch law. LUMC has delegated these responsibilities, especially the evaluation of expectedness, to the principal investigator of this study.
All SAE reports will be handled and assessed according to these legal requirements. Upon receipt of a SAE report at the IKW Trial Office at LUMC Leiden, the legal procedures for SAE and SUSAR will be followed. The principal investigator of the study will be promptly notified and will assess the causality of the SAE as not, unlikely, possibly, probably or definitely related to the study medication; for this assessment the other Study Coordinators and the Data and Safety Monitoring Board may be consulted. If such relation exists, the event is considered a Serious Adverse Drug Reaction. If the nature and/or severity of the SADR are not consistent with the product information of these registered and commonly used drugs, as assessed by the principal investigator of the study, the event is a SUSAR and all legal requirements regarding reporting will be followed (see 9.3.4).
Any SAE occurring after the 30-day period, throughout follow-up, should be reported promptly if considered possibly, probably or definitely related to the protocol treatment.

9.3.4. Summary of procedures for reporting of SAE and SUSAR and delegated responsibilities to the Country Coordinating Centres in the participating countries

- **SAE**:
  - **Netherlands**: local investigator to send **< 24 h** to IKW Trial Office at LUMC (fax +31 71 526 6712)
  - **All other participating countries**: local investigator to send **< 24 h** to Country Coordinating Centre and copy to IKW Trial Office at LUMC (see country/group specific Appendices for directions)
  - Completed SAE information with comments to be sent by Country Coordinating Centre **< 24 h** to LUMC
  - evaluation of expectedness at LUMC; if SUSAR: see below
- **SUSAR**:
  - LUMC to report to Dutch competent authorities (CA), ethics committee (EC), Country Coordinating Centres, DSMB, Dutch local investigators, Country/Group Study Coordinators: **within 7 days** (if death or life threatening, with additional information within the 8 subsequent days); or (in other cases) **within 15 days –** NB: report to Country Coordinating Centres ***within 5 days*** (for fatal/life threatening events) and ***12 days*** (for all other events) to ensure CCC will be able to forward to Country CA and EC within legal timeframes
  - Country Coordinating Centres to send this report **within 24-48 h** on to Country CA and EC, to Country local investigators, and to other authorities if required by their law. Country Coordinating Centre are responsible for SUSAR reporting according to their country’s law, and for European Countries- to report their own SUSAR to Eudravigilance according to their country’s law.

- **Annual safety reports:**
  - Annual report of all SAE will be sent in October each year to the Country Coordinating Centres, DSMB, Central Ethics Committee, Dutch competent authorities, Country Study Coordinators, and Dutch local investigators

- Country Coordinating Centres to send this report on to Country competent authorities, Country ethics committees, Country local investigators, and to other authorities if required by their law

10. Registration and randomisation

10.1 Registration

Patients who are eligible for the study should be referred to the radiation oncologist immediately after the operation. Preferably, the gynecologist already mentions the trial and briefly explains its principles, however full patient information should be done after confirmation of eligibility by pathology review (see 8.1 and Appendix A). The radiation oncologist further explains the rationale and design of the trial and the respective treatment procedures, and arranges an appointment with the medical oncologist for evaluation of potential contra-indications for chemotherapy. The radiation oncologist hands out the patient information. If the medical oncologist consents to chemotherapy and if informed consent is obtained, the radiation oncologist contacts the Data Center for registration and randomisation. The patient should be registered at the Data Center by phone call.

The information which will be requested at registration is summarized on the randomization checklist, which should be filled in prior to registration. Each patient will be given a unique trial number. To ensure patient privacy, the patient will be registered by trial number and patient code (first initial and first two letters of maiden name or Participating Group-specific name code), and these will be used for the database, follow-up information and correspondence. Date of birth will only be noted as item on the registration form, as age is an important prognostic factor in endometrial carcinoma. The pathology number will be asked to ensure receipt of the correct slides and tissue sample, however, the storage of samples will be done using study number only.

10.2. Randomisation

Central randomisation will be done with stratification by FIGO stage, participating group, mode of surgery, and histological type. The trial number and result of randomisation will be given immediately by phone or via Internet and confirmed by email.

11. Quality of life assessment

For the evaluation of the general quality of life the EORTC (European Organisation for Research and Treatment of Cancer) Core questionnaire (QLQ-C30 version 3.0) will be used (Appendix G). The EORTC QLQ-C30 is a multidimensional, cancer-specific quality of life questionnaire developed by the EORTC Study Group on Quality of Life (QOL) for repeated assessments within clinical trials. It is developed in a cross-cultural setting and has been found valid and reliable for quality of life assessments in various cancer populations, irrespective of the specific diagnosis. Optional modules developed for specific diagnostic groups or specific treatment modalities can supplement it. The QLQ-C30 contains five functional scales (physical, cognitive, emotional, social and role functioning), a global health status/quality of life scale, three symptom scales (pain, fatigue and nausea/vomiting), and six single items assessing additional symptoms (dyspnoea, insomnia, loss of appetite, constipation, diarrhoea) and perceived financial impact. For the majority of the QLQ-C30 items a 4-point Likert-type response scale is used. Exceptions are the items for the global quality

of life scale (were a 7-point scale is used). All subscale and individual item responses are linearly converted to 0 to 100 scales. A higher score for a functional and global quality of life scales represents a better level of functioning. For the symptom scales and items, a higher score reflects a higher level of symptoms and decreased quality of life.
The EORTC Study Group on QOL has designed or is in the process of designing and validating various specific modules, such as the module for prostate cancer (PR25), head and neck cancer (H&N35), ovarian cancer (OV28), and cervix cancer (CX24). However, there is currently no EORTC- or other diagnosis-specific module for endometrial cancer. The EORTC Quality of Life Group has just started phase I of the module development process for an endometrial cancer module, based on the module for cervix cancer (CX24).
To evaluate specific symptoms occurring after radiation therapy and chemotherapy for endometrial cancer, we have decided in conjunction with the EORTC Quality of Life Group to use the CX-24 module, supplemented with the subscale for chemotherapy from the ovarian cancer module (OV28), see Appendix G.

During informed consent the patient will be asked by the radiation oncologist to participate in the quality of life (QoL) analysis. (Note: for non-Dutch institutions, see Group-Specific appendices for specific instructions on Quality of Life Questionnaire collection). If the patient consents, the radiation oncologist hands over the baseline QoL questionnaire and an address sheet with a pre-stamped return envelope. The patient is asked to fill out her name and address on the address sheet for the sole purpose of enabling the Data Center to send the subsequent QOL questionnaires to her home address. If the patient declines or if local regulations refuse collection of name and address details, the QoL questionnaire collection is left to the responsibility of the local investigator, however, in practice patients readily consent as they consider the quality of life assessments valuable.
After receiving the baseline questionnaire and address sheet at the Data Center, the patient's name and address information will be entered in a separate database, which will exclusively be used for sending out QoL questionnaires.
QoL questionnaires will be handed out by the radiation oncologist at baseline and at the completion of radiotherapy. From then on, the QOL questionnaires will be sent directly to the patient's home address at 6, 12, 18, 24, 36 and 60 months from the date of randomisation.
In case of distant metastases or locoregional relapse not amenable to treatment with curative intent, QoL questionnaires can be discontinued.

12. Statistical considerations

12.1 Number of patients and power calculation

Based on incidence and survival rates provided by the Comprehensive Cancer Centers West and South, the estimated number of patients who will be eligible for the trial per year in The Netherlands is 200, with a frequency distribution of 25% stage IC grade 3; 10% stage II grade 3; 50% stage III; and 15% serous/clear cell histology. Assuming a 50% accrual rate to the study, the calculations have been based on a yearly accrual rate of 100 patients per year.

The study is based on a recruitment period of 5 years, and a follow-up duration of 30 months after inclusion of the last patient before definite analysis. It is expected that the yearly accrual rate will be 100 patients. Thus a total of 500 patients may be accrued in a 5-year period.
The principal aim is to detect with sufficient power (80%) a difference in the 5-year overall survival (OS) rate of 11.5% (based on an expected 5-year OS in the RT arm of 65%; hazard ratio for CMT 0.62) with a two–sided test at significance level alpha=0.05. This requires 145 events to be observed, and a minimum target number of 487 patients for this trial. With this number of patients, the power to detect a difference in 5-year OS of 12.5% (HR for CMT 0.59) is 87.5%. The minimum target number for the trial will thus be 500 patients, to ensure a total of 487 eligible and evaluable patients.
Collaboration in the Intergroup setting will ensure sufficient and timely patient inclusion in this trial. If it turns out that in the first two years after other Groups are open, significantly more than 100 patients per year can be enrolled, the target number will be increased to 670 patients within the 5-year accrual period, in order to be able to detect a 10% difference in 5-year OS (HR for CMT 0.67) with a power of 80% (198 events required, minimum number of patients 655).
Evaluation of accrual and event rates at Annual Accrual and Safety Reporting and DSMB meeting in October 2011 has shown that extension of the recruitment target to 670 patients is feasible and desirable, as this will ensure that
- the study is powered (80%) to detect a difference of 10% (HR 0.67) in 5–year overall survival (65 to 75%), as summarized in the previous paragraph;
- in case of a lower than expected event rate, the statistical endpoints of the study will still be met and reliable results will be obtained. If 5-year OS in the control arm would be 75% (instead of 65%), the required minimum sample size would 600 patients.
After recommendation by TMG with approval and support of the Data and Safety Monitoring Board, the extension of recruitment target to 670 patients was requested and granted by the Dutch Cancer Society and approved by the Central Ethics Committee.

12.1.1 Analysis of the co-primary endpoint failure-free survival

The study was powered on the required number of overall survival (OS) events. As with high-risk endometrial cancer virtually all recurrences are expected to lead to death in about 1-4 years, the required number of events for the co-primary endpoint failure-free survival (FFS) was estimated to be the same as for overall survival.
Analysis of the co-primary endpoint FFS will be done at the same time as analysis of OS. It is essential to define the alpha partitioning for this analysis. The aim is to secure an overall alpha level of 0.05, for the analyses performed for FFS and OS, taking into account the interim analysis performed for OS (see 12.2).
If the two (log-rank) test statistics z_1 and z_2 are independent, each of the tests can be performed at a nominal level of 0.0253, to obtain an overall alpha level of 0.05. Since it is expected that the two test statistics will be positively correlated, a nominal alpha (α) level higher than 0.0253 is expected to still yield an overall α level of 0.0498 (see 12.2). In their paper on analysis of multiple endpoints in clinical trials, Pocock *et al** (1987) have included a table containing that nominal α which the smaller of the 2 *P*-values obtained from z_1 and z_2 will reach with probability α under the null hypothesis for a given correlation of the two test statistics. The

question is what that correlation will be. By a slight extension of the results of Tsiatis[#] (1981) it can be seen that in our situation (if analysis is performed after d_1 FFS and d_2 OS events have been observed) the correlation between z_1 and z_2 equals dcommon $/\sqrt{d_1 * d_2}$, where dcommon is the number of events that are common in both analyses, i.e. the number of cancer or treatment deaths. An estimate of this number was obtained from event counts on May 1st, 2016. Based on simulation studies, the correlation between FFS and OS events was estimated to be between 0.85-0.9 at the time of final analysis. With the actual correlation at the time of final analysis of 0.85, 0.875 or 0.9, respectively, a nominal alpha of 0.0328, 0.0337 or 0.0349, respectively, for each of the two analyses will result in an overall α level of 0.0498. Note that the correlation and nominal alpha mentioned above are projections based on current data. The actual correlation (and hence nominal alpha) to be used will be determined at statistical analysis as dcommon $/\sqrt{d_1 * d_2}$, with dcommon, d_1 and d_2 as observed at that time.

* Pocock SJ, Geller NL, Tsiatis AA (1987). The analysis of multiple endpoints in clinical trials. Biometrics, 43, 487-498.
[#] Tsiatis AA (1981). The asymptotic joint distribution of the efficient scores test for the proportional hazards model calculated over time. Biometrika, 68, 311-15.

12.2 Stopping rule, safety reviews and interim analyses

An Independent Data and Safety Monitoring Board (DSMB), consisting of at least two clinicians not entering patients into the trial, an independent statistician, and a representative from each participating group, will be appointed to monitor the study.
Safety reviews are planned primarily to guard against unfavorable results in the combined modality (CMT) arm. Death and failure rates and SAE reports for both treatment arms will be closely monitored in order to pick up any (unexpected) trends. Safety reviews will be presented confidentially to the DSMB every year, and/or at request of the DSMB. These annual reviews will include data on SAEs, number and causality of deaths, number of treatment failures and serious adverse events. Only if the DSMB recommends that the study should be stopped or modified, the results will be made public to the principal investigators.

Interim analyses are planned after reaching one-third and two-thirds of the required number of 145 OS events, thus after 48 and 96 OS events. In order to maintain an overall alpha of 0.05, the nominal alpha levels for the first and second interim analysis will be 0.0002 and 0.012, respectively. The final analysis will then be performed with a nominal alpha of 0.0463 (see paragraph 12.2.1). Under the alternative hypothesis (superiority of the CMT arm), the probabilities of stopping at first and second interim analysis are 0.019 and 0.399, respectively.
At each interim analysis a detailed report will be generated and presented to the DSMB. The report includes by treatment arm the number of entered and at that time evaluable patients; treatment given; the number of deaths and causes of death; number of failures and types of failure; incidence, types and grades of adverse events. The DSMB is free in its public recommendations to the Study Coordinators and confidential recommendations to the study statistician, but the following guidelines apply.

1. Proven superiority of the CMT arm at the first and second interim analyses with alpha levels of 0.0002 and 0.012, respectively, is considered a reason for early stopping of the trial

2. If at the first and second interim analyses futility is suggested, i.e. if the confidence interval for the hazard ratio of the CMT arm corresponding to the appropriate alpha level used in the interim analysis does not contain the alternative hypothesis (0.62 or 0.67, respectively), this is considered a reason for early stopping of the trial for futility.

12.2.1. Update and amendment to paragraph 12.2

The first interim analysis has taken place in 2013, and the trial was closed to recruitment on 20 December 2013. In 2016 it was decided - with permission of the DSMB - **not** to perform the second interim analysis, as this would have no consequences to the trial and would reduce alpha spending.
Thus, in order to maintain an overall alpha of 0.05, with a nominal alpha level for the first interim analysis of 0.0002, the final analysis will be performed with a nominal alpha of 0.0498.

12.3 Statistical analysis

All analyses concerning treatment effects will be done according to the intention-to-treat principle. The main endpoints for the comparison of the two treatment arms are overall survival and the rate of failure (failure defined as relapse, or death due to endometrial carcinoma or due to treatment complications) from registration. Secondary endpoints are relapse free survival, overall locoregional failure, and overall distant failure. Formal tests for the differences in relapse and survival rates between the two arms will be done with the Kaplan-Meier method, the log-rank test and Cox regression analysis. The analysis of treatment toxicity will be done by comparing the incidence of acute side effects with logistic regression. The incidence of late side effects will be analyzed actuarially with the Kaplan-Meier method, the log-rank test and Cox regression analysis. Multivariate analysis of prognostic factors, especially stage, histological grade, and mode of surgery will be done using logistic and Cox regression analyses.

12.4 Statistical analysis of the quality of life assessment

All patients with a valid baseline and at least one follow-up QOL questionnaire will be included in the analysis. The baseline questionnaire is considered valid if filled out and dated by the patient before the starting date of trial treatment. Reasons for missing baseline and follow-up questionnaires will be assessed. To evaluate the differences between the treatment groups with respect to the effect of treatment burden on life-quality during and up to 5 years after treatment, the repeated measures of the QLQ-C30 and CX24/OV28 functional and symptom scales and of the global health index will be analysed using mixed ANOVA models. The single items in the QLQ-C30 and CX24/OV28 will be analysed using (ordinal) logistic regression with random effects. Missing data of patients dropping out of the study will be handled as missing-at-random; the appropriateness of this assumption will be assessed by fitting a joint model to survival and QOL-data or by fitting pattern-mixture models. The items concerning the diagnosis-specific symptoms will be summarized using the unweighed sumscore. The reliability and validity of this sumscore will be established using baseline data, and -when sufficient- the effect of treatment on this sumscore will be evaluated using mixed ANOVA models.

13. Ethics

The study protocol and any amendment that is not solely of an administrative nature will be submitted for approval by the Institutional Ethics Committee (METC). In the law (Wet medisch-wetenschappelijk onderzoek met mensen, WMO) rules for the scientific and ethical review of trials involving human subjects have been formulated. The guidelines “richtlijn toetsingsprocedure multicenter-onderzoek” (active as of January 1, 2001) and “good clinical practice” will be applicable. The protocol will be submitted for review to the LUMC Medisch-Ethische Toetsings Commissie (METC), which will contact the Board of Directors of the participating centers for statements of local consent.
The study will be conducted in full conformance with the ethical principles of the Declaration of Helsinki and the WMO.
The rationale, design and aims of the study will be explained to each patient along with the specific information on the respective treatment arms. The principles of randomisation and registration and the follow-up procedure will be clarified. The patient will receive written patient information (see Appendix G) and will have ample opportunity to ask questions. The patient will have sufficient time to consider the study before deciding to participate. Written informed consent of the patient is required before randomisation. This consent will include registration in the trial, data processing and sending diagnostic material for pathology review.
An Independent Data and Safety Monitoring Board (DSMB) and (for UK) an independent Trial Steering Committee (TSC) will be appointed to supervise the trial, ensure its conduct is according to GCP, and to provide advice to the study coordinators on continuing or stopping the trial, or modifying the protocol (see section 12.2)

14. Trial insurance

According to the law (WMO), every participating institute should have an insurance against the legal liability resulting from medical procedures. In addition, specific trial insurance will be organized as requested by the Central Ethical Committee. Patients will receive written information on the trial insurance for this study, if applicable by country regulations.

15. Publication policy

The final publication of the trial results will be written by the study coordinators on the basis of the statistical analyses performed by the trial statistician(s). A draft manuscript will be submitted to all co-authors for review. After revision by the co-authors, the manuscript will be sent to a peer-reviewed scientific journal. Authors will include the study coordinators, the lead investigators of cooperating major groups, investigators from the participating centres who have included more than 10% of the evaluable patients in the trial (by order of inclusion), the statistician(s), the review pathologist(s), and others who have made significant scientific contributions. A listing of all participating investigators will be included in an appendix to the publications. Publications regarding specific sub-analyses or side studies (e.g. pathology) will be written by the respective lead investigators, in cooperation with the study coordinators.
Any publication, abstract or presentation involving patients included in this trial must be approved by the study coordinators. Such a publication cannot include any comparisons between randomised treatment

arms, nor an analysis of any of the study endpoints unless the final results of the trial have already been published. Interim publications or presentations of the study may include demographic data, overall results and prognostic factor analyses, but no comparisons between randomised treatment arms may be made public before the recruitment is discontinued.

16. List of participating centres and local investigators

16.1. The Netherlands - DGOG

1. Academic Medical Center Amsterdam (L. Uitterhoeve, J. vd Velden, A. Westermann)
2. Arnhem Radiotherapy Institute ARTI (E. van der Steen-Banasik)
3. Catharina Hospital Eindhoven (M. Lybeert)
4. Dr B. Verbeeten Institute, Tilburg (K. De Winter)
5. Erasmus Medical Center Rotterdam (JW Mens, H. van Doorn, R. de Wit)
6. Leiden University Medical Center (C. Creutzberg, J. Kroep, K. Gaarenstroom)
7. NKI/v. Leeuwenhoekhuis (B. van Triest, W.van Driel)
8. MAASTricht Radiation Oncology (L. Lutgens, R. Kruitwagen)
9. Radiotherapy Center West / MCH, Den Haag (T.Stam, M. Kagie)
10. Medical Spectre Twente, Enschede (J. Jobsen, E. Schutter)
11. Radiotherapy Institute Friesland (A. Slot)
12. Radiotherapy Institute Stedendriehoek (S. van de Pol)
13. Sophia Hospital Zwolle (P. Timmer)
14. University Medical Center Groningen (H. Nijman, B. Pras, A.Reijners)
15. University Medical Center Radboud (J. de Hullu, A.Snyers, N. Ottevanger)
16. University Medical Center Utrecht (I. Jürgenliemk-Schulz, E. Witteveen)
17. VU Medical Center (O. Meijer)
18. Zeeuws Radiotherapy Institute (V. Coen)

16.2. UK – NCRI

1. Barts and the London NHS Trust, London
2. Basildon and Thurrock University Hospitals, Basildon, Essex
3. Beatson West of Scotland Cancer Centre, Glasgow, Scotland
4. Belfast Health and Social Care Trust, Belfast
5. BHR Hospitals NHS Foundation Trust, Queen's Hospital, Romford
6. Brighton and Sussex University Hospitals NHS Trust, Brighton
7. Buckinghamshire Healthcare NHS Trust (includes both Stoke Mandeville Hospital & Wycombe Hospital)
8. Cambridge University Hospitals NHS Foundation Trust, Cambridge
9. Cheltenham General Hospital, Cheltenham
10. Clatterbridge Centre for Oncology NHS Foundation Trust
11. Cumberland Infirmary, North Cumbria University Hospitals NHS Trust, Carlisle
12. Essex County Hospital, Colchester Hospital University NHS Foundation Trust, Colchester
13. Gloucestershire Royal Hospitals, Gloucester
14. Guy's and St Thomas' Hospital NHS Trust, London (includes both Guy’s Hospital & St Thomas’ Hospital)
15. Hammersmith Hospital NHS Trust, London
16. Hereford County Hospital, Hereford
17. Hinchingbrooke Health Care NHS Trust

18. James Paget University Hospitals NHS Foundation Trust
19. Lancashire Teaching Hospitals NHS Foundation Trust, Royal Preston Hospital
20. Mount Vernon Hospital, East North Hertfordshire NHS Trust, Hertfordshire
21. Norfolk and Norwich University Hospitals NHS Foundation Trust, Norwich
22. Nottingham City Hospital, Nottingham University Hospitals NHS Trust, Nottingham
23. Oxford Radcliffe Hospital NHS Trust
24. Peterborough And Stamford Hospitals NHS Foundation Trust
25. Poole Hospital NHS Foundation Trust, Poole
26. Portsmouth Hospitals NHS Trust
27. Royal Berkshire NHS Foundation Trust, London Road, Reading
28. Royal Cornwall Hospital, Truro, Cornwell
29. Royal Devon & Exeter NHS Foundation Trust
30. Royal Marsden NHS Foundation Trust, London
31. Royal Surrey County Hospital, Guildford, Surrey
32. South Tees Hospitals NHS Trust, The James Cook University Hospital, Middlesbrough
33. Southampton University Hospitals NHS Trust, Southampton
34. Southend University Hospital NHS Foundation Trust
35. Taunton And Somerset NHS Foundation Trust
36. The Christie NHS Foundation Trust, Manchester
37. The Royal Wolverhampton Hospitals NHS Trust
38. University College London Hospitals NHS Trust, London
39. University Hospitals Coventry and Warwickshire NHS Trust
40. University Hospitals of Leicester NHS Trust, Leicester
41. Western Sussex Hospitals NHS Trust, Worthing Hospital
42. Worcestershire Acute Hospitals NHS Trust

To be activated in 2012:

43. Betsi Cadwaladr University Health Board (includes both Glan Clywd & Ysbyty Gwynedd Hospitals)
44. Plymouth Hospitals NHS Trust
45. United Lincolnshire Hospitals NHS Trust, Lincoln (includes both Lincoln County & Pilgrim Hospitals)
46. Newcastle Hospitals NHS Foundation Trust, Newcastle
47. Northampton General Hospital NHS Trust
48. University Hospital of North Staffordshire NHS Trust

16.3. Canada - NCIC CTG:

1. Queen Elizabeth II Centre for Research, Halifax, Nova Scotia. (R.Grimshaw, J. Bentley)
2. Atlantic Health Sciences Corporation, Saint John, New Brunswick (J.Carson, M.Burnell)
3. Centre Hospitalier de Sherbrooke, Sherbrooke, Quebec (P. Bessette)
4. McGill Oncology, Montreal, Quebec (L. Portelance)
5. Hopital Notre Dame, Montreal, Quebec (D. Provencher, T.V.Nguyen)

6. Sunnybrook Health Sciences Centre, Toronto, Ontario (G. Thomas)
7. Princess Margaret Hospital, Toronto, Ontario (A.Fyles, B.Rosen)
8. London Regional Cancer Centre, London, Ontario (A.Hammond)
9. Windsor Regional Cancer Centre, Windsor, Ontario (S.Yoshida)
10. Saskatoon Cancer Centre, Saskatoon, Saskatchewan (C. Giede)
11. Tom Baker Cancer Centre, Calgary, Alberta (P. Ghatage)
12. Vancouver Island Cancer Centre, Victoria, B.C. (J.Michels)

16.4. Australia and New Zealand - ANZGOG

1. Auckland Hospital, Auckland, NZ (S. Brooks)
2. Christchurch Hospital, Christchurch, NZ (M. Vaughan)
3. Mater Adults Hospital, South Brisbane, Queensland (P. Mainwaring, J. Ramsay)
4. Monash Medical Centre/Peter MacCallum Cancer Centre Moorabbin, Victoria (P. Khaw, G. Goss)
5. Palmerston North Hospital, Palmerston North, NZ (C. Hardie)
6. Peter McCallum Cancer Centre, Melbourne, Victoria (L. Mileshkin)
7. Prince of Wales Hospital, Randwick, NSW (M. Friedlander)
8. Royal Brisbane & Women’s Hospital, Brisbane, Queensland (R. Cheuk)
9. Royal Hobart Hospital, Hobart, Tasmania (P. Blomfield)
10. Royal North Shore Hospital, Sydney, NSW (G. Lamoury)
11. Royal Prince Alfred Hospital, Sydney, NSW (C. Milross)
12. Waikato, Hamilton, NZ (M. Kuper)
13. Wellington Blood & Cancer Centre, Wellington, NZ (C. Johnson)
14. Westmead Hospital, Sydney, NSW (G. Wain)
15. Wollongong Hospital; Illawarra Cancer Care Centre, Sydney, NSW (K. Foo, A. Glasgow)
16. Calvary Mater Newcastle Hospital, Waratah, NSW (A. Capp)
17. Launceston General Hospital, Launceston, Tasmania (D. Byram)
18. Liverpool Hospital, Sydney, NSW (S. Vinod)

16.5. Italy - MaNGO Group

1. Policlino Consorzionale, Bari (G. Cormio)
2. Spedali Civili, Brescia (S. Pecorelli, G. Tognon)
3. Instituto Nazionale per la Ricerca sul Cancro, Genova (M. Bruzzone)
4. A. O. Ospedale di Lecco (A. Colombo)
5. Ulss 12 veneziana, Ospedale di Mestre (T. Maggino)
6. Istituto Nazionale dei Tumori, Milano (F. Raspagliesi, F. Zanaboni)
7. A. O. Niguarda, Milano (S. Siena)
8. A. O. San Gerardo, Monza (A. A. Lissoni)
9. Istituto Oncologico Veneto, Padova (M. O. Nicoletto)
10. A. O. “V. Cervello”, Palermo (D. Gueli Alletti)
11. Azienda Ospedaliera di Parma (G.B. Nardelli)

12. Azienda Ospedaliera di Pisa (A. Gadducci)
13. Ospedale S. Maria delle Croci, Ravenna (G. Comerci)
14. Arcispedale Santa Maria Nuova, Reggio Emilia (T. Palmieri)
15. Presidio Ospedaliero Sant'Anna, Torino (D. Katsaros)
16. Ospedale Mauriziano Umberto I, Torino (P. Zola)
17. A. O. Ospedale Treviglio-Caravaggio, Treviglio (R. Grassi)
18. Azienda Ospedaliera di Urbino (E. Fusco)
19. Ospedale "F. Del Ponte", Varese (N. Donadello)
20. Ospedale Civile Maggiore, Verona (T. Sava)

To be activated in 2012:

21. Ospedale Madre salus, Legnago
22. Poliambulanza Hospital, Brescia

16.6. Austria - Vienna

1. Medizinischen Universität Wien / AKH Wien (A. Sturdza / R. Pötter)

16.7. France – UNICANCER (FEDEGYN Group)

1. Institut Gustave Roussy, Villejuif (Ch. Haie-Meder)
2. Centre Oscar Lambret, Lille Cedex (A. Lesoin)
3. Centre Val d'Aurelle, Montpellier (C. Kerr)
4. Institut Curie, Paris Cedex (S. Scholl)
5. Institut Jean Godinot, Reims (S. Maillard)
6. Centre Paul Strauss, Strasbourg Cedex (Th. Petit)
7. Centre Francois Baclesse, Caen Cedex (D. Lerouge)
8. Centre Francois Baclesse, Marseille Cedex (F. Rousseau)
9. Centre Rene Gauducheau, Saint Herblain Cedex (D. Berton-Rigaud)
10. Institut Bergonie, Bordeaux Cedex (L. Thomas)
11. CHU Tours- Hopital Bretonneau, Tours Cedex (I. Barillot)
12. Centre Georges-Francois Leclerc, Dijon (K. Peignaux)
13. Centre Georges-Francois Leclerc, Vandoeuvre les Nancy Cedex (C. Brunaud)
14. Institut Claudius Regaud, Toulouse (L. Gladieff)
15. Centre Henri Becquerel, Rouen (Ch. Hanzen)
16. CHD Vendee, La Roche sur Yon (A. Zawadi)
17. Centre Jean Perrin, Clermont Ferrand (J-L Achard)
18. Centre Antoine Lacassagne, Nice (Ph. Follana)

19. References

1. Aalders J, Abeler V, Kolstad P, et al: Postoperative external irradiation and prognostic parameters in stage I endometrial carcinoma: clinical and histopathologic study of 540 patients. Obstet Gynecol 56:419-427, 1980
2. Creutzberg CL, van Putten WL, Koper PC, et al: Surgery and postoperative radiotherapy versus surgery alone for patients with stage-1 endometrial carcinoma: multicentre randomised trial. PORTEC Study Group. Post Operative Radiation Therapy in Endometrial Carcinoma. Lancet 355:1404-1411, 2000
3. Keys HM, Roberts JA, Brunetto VL, et al: A phase III trial of surgery with or without adjunctive external pelvic radiation therapy in intermediate risk endometrial adenocarcinoma: a Gynecologic Oncology Group study. Gynecol Oncol 92:744-751, 2004
4. ASTEC/EN.5 Study Group, Blake P, Swart AM, Orton J, Kitchener H, Whelan T, Lukka H, Eisenhauer E, Bacon M, Tu D, Parmar MK, Amos C, Murray C, Qian W. Adjuvant external beam radiotherapy in the treatment of endometrial cancer (MRC ASTEC and NCIC CTG EN.5 randomised trials): pooled trial results, systematic review, and meta-analysis. Lancet 2009 Jan 10;373(9658):137-46, Epub 2008.
5. Nout RA, Putter H, Jürgenliemk-Schulz IM, Jobsen JJ, Lutgens LCHM, van der Steen-Banasik EM, et al. Vaginal brachytherapy versus external beam pelvic radiotherapy for high-intermediate risk endometrial cancer: Results of the randomized PORTEC-2 trial. J Clin Oncol 26: 2008 (May 20 suppl; abstr LBA5503)
6. Straughn JM, Huh WK, Orr JW, et al: Stage IC adenocarcinoma of the endometrium: survival comparisons of surgically staged patients with and without adjuvant radiation therapy small star, filled. Gynecol Oncol 89:295-300, 2003
7. Greven KM, Randall M, Fanning J, et al: Patterns of failure in patients with stage I, grade 3 carcinoma of the endometrium. Int J Radiat Oncol Biol Phys 19:529-534, 1990
8. Creutzberg CL, van Putten WL, Warlam-Rodenhuis CC, et al: Outcome of high-risk stage IC, grade 3, compared with stage I endometrial carcinoma patients: the Postoperative Radiation Therapy in Endometrial Carcinoma Trial. J Clin Oncol 22:1234-1241, 2004
9. Naumann RW, Higgins RV, Hall JB: The use of adjuvant radiation therapy by members of the Society of Gynecologic Oncologists. Gynecol Oncol 75:4-9, 1999
10. Koh WJ, Tran AB, Douglas JG, et al: Radiation therapy in endometrial cancer. Best Pract Res Clin Obstet Gynaecol 15:417-432, 2001
11. Greven KM, Corn BW: Endometrial cancer. Curr Probl Cancer 21:65-127, 1997
12. Jereczek-Fossa BA: Postoperative irradiation in endometrial cancer: still a matter of controversy. Cancer Treat Rev 27:19-33, 2001
13. Briet JM, Hollema H, Reesink N, et al: Lymphvascular space involvement: an independent prognostic factor in endometrial cancer. Gynecol Oncol 96:799-804, 2005
14. Citron JR, Sutton H, Yamada SD, et al: Pathologic stage I-II endometrial carcinoma in the elderly: radiotherapy indications and outcome. Int J Radiat Oncol Biol Phys 59:1432-1438, 2004
15. Alektiar KM, McKee A, Lin O, et al: Is there a difference in outcome between stage I-II endometrial cancer of papillary serous/clear cell and endometrioid FIGO Grade 3 cancer? Int J Radiat Oncol Biol Phys 54:79-85, 2002
16. Creasman WT, Kohler MF, Odicino F, et al: Prognosis of papillary serous, clear cell, and grade 3 stage I carcinoma of the endometrium. Gynecol Oncol 95:593-596, 2004
17. Hamilton, C. A., Liu, L., Osann, K. N., Vasilev, S. A., Berman, M. L., Husain, A., Teng, N. N., Kapp, D. S., and Chan, J. K. Observation versus adjuvant treatment in early stage uterine serous carcinoma. Proc Int Gynecol Cancer Soc 2004, Abstract 57. 2005.
18. Creasman WT, Morrow CP, Bundy BN, et al: Surgical pathologic spread patterns of endometrial cancer. A Gynecologic Oncology Group Study. Cancer 60:2035-2041, 1987
19. COSA-NZ-UK endometrial cancer study group: Pelvic lymphadenectomy in high risk endometrial cancer. Int J Gynecol Cancer 6:102-107, 1996
20. Trimble EL, Kosary C, Park RC: Lymph node sampling and survival in endometrial cancer. Gynecol Oncol 71:340-343, 1998
21. Cragun JM, Havrilesky LJ, Calingaert B, et al: Retrospective Analysis of Selective Lymphadenectomy in Apparent Early-Stage Endometrial Cancer. J Clin Oncol 2005
22. Morrow CP, Bundy BN, Kurman RJ, et al: Relationship between surgical-pathological risk factors and outcome in clinical stage I and II carcinoma of the endometrium: a Gynecologic Oncology Group study. Gynecol Oncol 40:55-65, 1991
23. Mohan DS, Samuels MA, Selim MA, et al: Long-term outcomes of therapeutic pelvic lymphadenectomy for stage I endometrial adenocarcinoma [see comments]. Gynecol Oncol 70:165-171, 1998
24. Fanning J, Nanavati PJ, Hilgers RD: Surgical staging and high dose rate brachytherapy for endometrial cancer: limiting external radiotherapy to node-positive tumors. Obstet Gynecol 87:1041-1044, 1996
25. Kilgore LC, Partridge EE, Alvarez RD, et al: Adenocarcinoma of the endometrium: survival comparisons of patients with and without pelvic node sampling. Gynecol Oncol 56:29-33, 1995
26. ASTEC study group, Kitchener H, Swart AM, Qian Q, Amos C, Parmar MK. Efficacy of systematic pelvic lymphadenectomy in endometrial cancer (MRC ASTEC trial): a randomised study. Lancet 2009 Jan 10; 373(9658):125-36. Epub 2008.

27. Benedetti Panici P, Basile S, Maneschi F, Lissoni AA, et al: Systematic pelvic lymphadenectomy vs no lymphadenectomy in early-stage endometrial carcinoma: Randomized clinical trial. J Natl Cancer Inst 100:1–10, 2008
28. Thigpen JT, Brady MF, Homesley HD, et al: Phase III trial of doxorubicin with or without cisplatin in advanced endometrial carcinoma: a gynecologic oncology group study. J Clin Oncol 22:3902-3908, 2004
29. Aapro MS, Van Wijk FH, Bolis G, et al: Doxorubicin versus doxorubicin and cisplatin in endometrial carcinoma: definitive results of a randomised study (55872) by the EORTC Gynaecological Cancer Group. Ann Oncol 14:441-448, 2003
30. Fleming GF, Filiaci VL, Bentley RC, et al: Phase III randomized trial of doxorubicin + cisplatin versus doxorubicin + 24-h paclitaxel + filgrastim in endometrial carcinoma: a Gynecologic Oncology Group study. Ann Oncol 15:1173-1178, 2004
31. Fleming GF, Brunetto VL, Cella D, et al: Phase III trial of doxorubicin plus cisplatin with or without paclitaxel plus filgrastim in advanced endometrial carcinoma: a Gynecologic Oncology Group Study. J Clin Oncol 22:2159-2166, 2004
32. Santin AD, Bellone S, O'Brien TJ, et al: Current treatment options for endometrial cancer. Expert Rev Anticancer Ther 4:679-689, 2004
33. Hoskins PJ, Swenerton KD, Pike JA, et al: Paclitaxel and carboplatin, alone or with irradiation, in advanced or recurrent endometrial cancer: a phase II study. J Clin Oncol 19:4048-4053, 2001
34. Scudder SA, Liu PY, Wilczynski SP, et al: Paclitaxel and carboplatin with amifostine in advanced, recurrent, or refractory endometrial adenocarcinoma: a phase II study of the Southwest Oncology Group. Gynecol Oncol 96:610-615, 2005
35. Morrow CP, Bundy BN, Homesley HD, et al: Doxorubicin as an adjuvant following surgery and radiation therapy in patients with high-risk endometrial carcinoma, stage I and occult stage II: a Gynecologic Oncology Group Study. Gynecol Oncol 36:166-171, 1990
36. Randall ME, Filiaci VL, Muss, H, et al: Randomized Phase III trial of whole abdominal irradiation versus doxorubicin and cisplatin chemotherapy in advanced endometrial carcinoma: a GOG study. J Clin Oncol 24(1): 36-44, 2006.
37. Susumu N, Sagae S, Udagawa Y, Niwa K, Kuramota H, Satoh S, Kudo R: Randomized phase III trial of pelvic radiotherapy versus cisplatin-based combined chemotherapy in patients with intermediate- and high-risk endometrial cancer: A Japanese Gynecologic Oncology Group study. Gynecol Oncol 108: 226-233, 2008.
38. Maggi R, Lissoni A, Spina F, Melpignano M, Zola P, Favalli G, et al. Adjuvant chemotherapy vs. radiotherapy in high-risk endometrial carcinoma: results of a randomised trial. Br J Cancer 2006;95:266-271
39. Mundt AJ, McBride R, Rotmensch J, et al: Significant pelvic recurrence in high-risk pathologic stage I--IV endometrial carcinoma patients after adjuvant chemotherapy alone: implications for adjuvant radiation therapy. Int J Radiat Oncol Biol Phys 50:1145-1153, 2001
40. Frigerio L, Mangili G, Aletti G, et al: Concomitant radiotherapy and paclitaxel for high-risk endometrial cancer: first feasibility study. Gynecol Oncol 81:53-57, 2001
41. Reisinger SA, Asbury R, Liao SY, et al: A phase I study of weekly cisplatin and whole abdominal radiation for the treatment of stage III and IV endometrial carcinoma: a Gynecologic Oncology Group pilot study. Gynecol Oncol 63:299-303, 1996
42. Greven K, Winter K, Underhill K, et al: Final analysis of RTOG 9708: Adjuvant postoperative irradiation combined with cisplatin/paclitaxel chemotherapy following surgery for patients with high-risk endometrial cancer. Gynecol Oncol 2006;103:155-159
43. Hogberg T, Rosenberg P, Kristensen G., et al. A randomized phase-III study on adjuvant treatment with radiation (RT) ± chemotherapy (CT) in early-stage high-risk endometrial cancer (NSGO-EC-9501/EORTC 55991). J Clin Oncol 2007, ASCO Annual Meeting Proceedings Part I. Vol 25, No.18S: 5503.
44. Smith TJ, Khatcheressian J, Lyman GH, et al. 2006 Update of ASCO Recommendations for the Use of White Blood Cell Growth Factors: An Evidence-Based Clinical Practice Guideline. J Clin Oncol 2006;24(19):3187-205.
45. FIGO Committee on gynaecologic oncology. Revised FIGO staging for carcinoma of the vulva, cervix, and endometrium. Int J Gynecol Obstet 2009;105:103-4.
46. Khalifa MA, Dodge J, Covens A, et al: Slide review in gynecologic oncology ensures completeness of reporting and diagnostic accuracy. Gynecol Oncol 90: 425-430, 2003

APPENDIX A. SUMMARY PORTEC-3

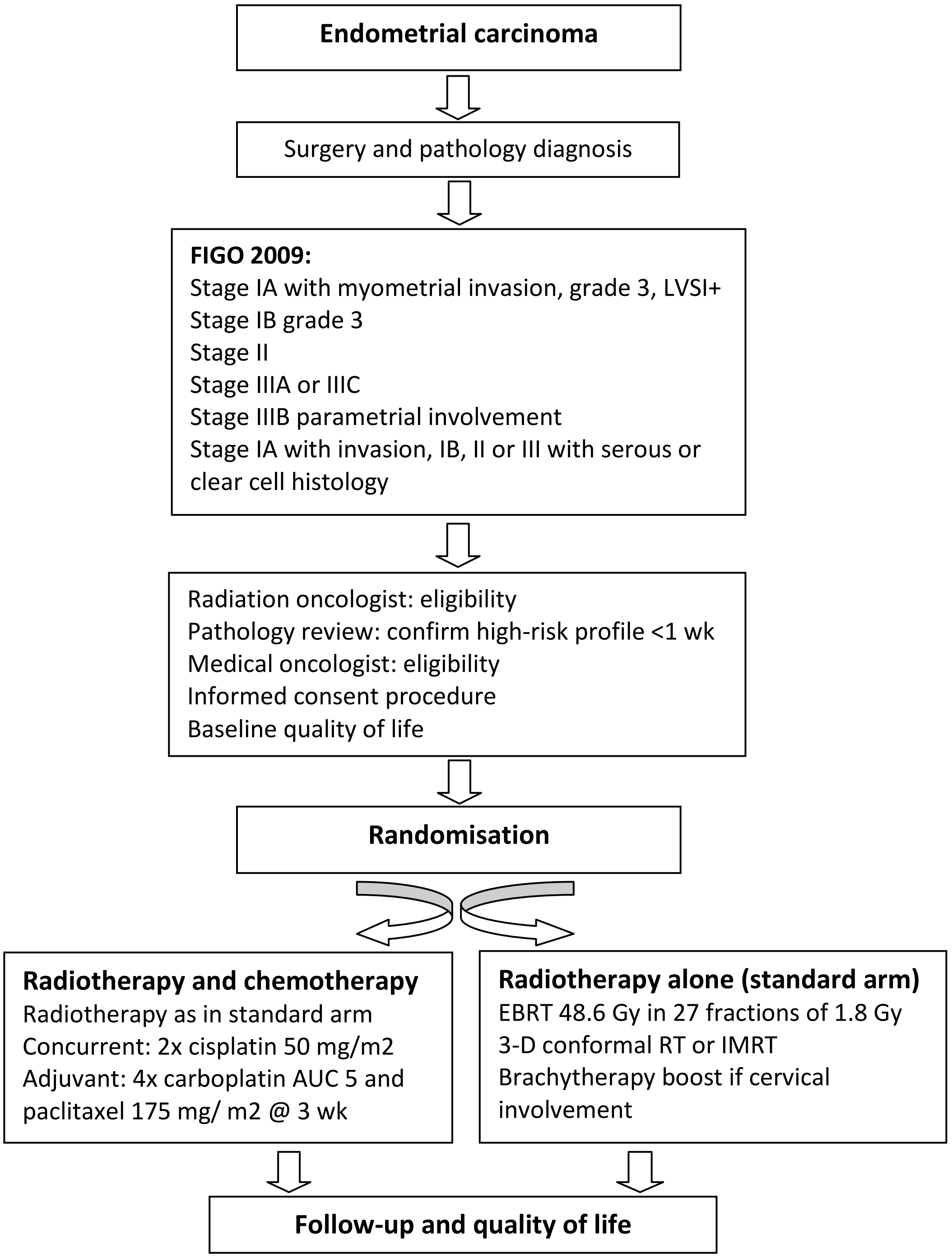

APPENDIX B. FIGO STAGING

FIGO 1988 staging for carcinoma of the corpus uteri

**Stage I**	**Tumor confined to corpus uteri**
stage IA	Tumor limited to endometrium
stage IB	Tumor invades up to less than one half of myometrium
stage IC	Tumor invades to more than one half of myometrium
**Stage II**	**Tumor invades cervix but does not extend beyond uterus**
stage IIA	Endocervical glandular involvement only
stage IIB	Cervical stromal invasion
**Stage III**	**Local and/or regional spread**
stage IIIA	Tumor involves serosa and/or adnexa (direct extension or metastasis) and/or cancer cells in ascites or peritoneal washings
stage IIIB	Vaginal involvement (direct extension or metastasis)
stage IIIC	Metastasis to pelvic and/or para-aortic lymph nodes
**Stage IV**	**Invasion in other organs and/or distant metastasis**
stage IVA	Tumor invades bladder mucosa and/or bowel mucosa
stage IVB	Distant metastasis (*excluding:* metastasis to vagina, pelvic serosa, or adnexa, *including:* metastasis to intra-abdominal lymph nodes other than para-aortic and/or inguinal lymph nodes)

FIGO 2008 staging for carcinoma of the endometrium

**Stage I***	**Tumor confined to the corpus uteri**
stage IA*	No or less than half myometrial invasion
stage IB*	More than half myometrial invasion
**Stage II***	**Tumor invades cervical stroma, but does not extend beyond the uterus****
**Stage III***	**Local and/or regional spread of the tumor**
stage IIIA*	Tumor invades the serosa of the corpus uteri and/or adnexae#
stage IIIB*	Vaginal and/or parametrial involvement#
stage IIIC*	Metastasis to pelvic and/or para-aortic lymph nodes#
IIIC1*	Positive pelvic lymph nodes
IIIC2*	Positive para-aortic lymph nodes with or without pelvic nodes
**Stage IV***	**Tumor invades bladder and/or bowel mucosa, and/or distant metastasis**
stage IVA*	Tumor invasion of bladder and/or bowel mucosa
stage IVB*	Distant metastasis, including intra-abdominal metastases and/or inguinal lymph nodes

* Either G1, G2 or G3 (G is FIGO grade)

** Endocervical glandular involvement only should be considered as Stage I and no longer as Stage II

Positive cytology has to be reported separately, without changing the stage.

APPENDIX C. PERFORMANCE STATUS (WHO-ECOG)

**Grade 0**	Fully active, able to carry out all normal (pre-disease) activity without restriction
**Grade 1**	Restricted in physically strenuous activity but ambulatory and able to carry out light work, e.g., light house work, office work
**Grade 2**	Ambulatory and capable of all self-care but unable to carry out any work activities. Up and about more than 50% of waking hours
**Grade 3**	Capable of only limited self-care; confined to bed or chair more than 50% of waking hours
**Grade 4**	Completely disabled; cannot carry out any self-care; totally confined to bed or chair

APPENDIX D. HISTOLOGIC CLASSIFICATION AND GRADING SYSTEM

International Society of Gynecologic Pathologists Classification for Endometrial Carcinomas

1. Endometrial adenocarcinoma
   - Papillary/villoglandular
   - Secretory
   - Ciliated cell
   - Adenocarcinoma with squamous differentiation
2. Mucinous carcinoma
3. Serous carcinoma
4. Clear-cell carcinoma
5. Squamous carcinoma
6. Undifferentiated carcinoma
7. Mixed types
8. Miscellaneous carcinoma
9. Metastatic carcinoma

Histologic classification of mixed carcinomas:
Mixed serous and endometrioïd carcinomas and mixed clear cell and endometrioid carcinomas should be classified as serous or clear cell carcinomas if they contain **at least 25%** of a serous or clear cell component, respectively, and otherwise be classified as endometrioid. However, for these mixed carcinomas the percentage of each component should be recorded.

International Federation of Gynecology and Obstetrics (FIGO) and Armed Forces Institute of Pathology (AFIP) histologic grading system

G1 tumors have 5% or less of a nonsquamous or nonmorular solid growth pattern

G2 tumors have 6% to 50% of a nonsquamous or nonmorular solid growth pattern

G3 tumors have more than 50% of a nonsquamous or nonmorular solid growth pattern

A higher degree of nuclear atypia (in comparison with the architectural grade) raises the grade of a G1 or G2 tumor by 1.

Adenocarcinomas with squamous differentiation are graded according to the nuclear grade of the glandular component.

APPENDIX E. RADIOTHERAPY SPECIFICATONS AND QUALITY ASSURANCE

Description of the Clinical Target Volume (CTV)

The CTV should include the upper 50% of the vagina, the vaginal tissues cranial to the vaginal marker, the paravaginal / parametrial soft tissues, and the distal common, external, and internal iliac lymph node regions as specified in section 7.3.1. (page 13-14). Inclusion of the subaortic pre-sacral nodes is recommended for tumors with involvement of the cervix.

Contouring of the CTV should be done according to literature data and taking institutional preferences and practices into account.

Useful guidelines and contouring atlas can be found at:

- RTOG website (consensus guidelines for delineation of the CTV): http://www.rtog.org/pdf_file2.html?pdf_document=GYN-Atlas.pdf
- Taylor A, Rockall AG, Reznek RH, Powell MEB. Mapping pelvic lymph nodes: guidelines for delineation in intensity-modulated radiotherapy. Int J Radiat Oncol Biol Phys. 2005;63:1604-12 and contouring atlas:
- Taylor A, Rockall AG, Powel MEB. An Atlas of the Pelvic Lymph Node Regions to Aid Radiotherapy Target Volume Definition. Clinical Oncology 2007 doi: 10.1016/j.clon.2007.05.002
- Small W Jr., Mell LK, Anderson P et al. Consensus guidelines for the delineation of the clinical target volume for intensity modulated pelvic radiotherapy in the postoperative treatment of endometrial and cervical cancer. Int J Radiat Oncol Biol Phys 2007, Nov 23

General guideline for contouring CTV:

The superior portion of the CTV should be defined by adding a 7 mm margin around the common iliac vessels seen on the axial CT slice. The CTV should be extended to include adjacent visible or suspicious lymph nodes, lymphoceles, and pertinent surgical clips, and modified to exclude bone, psoas muscle, and bowel. The CTV should follow the external and internal iliac vessels bilaterally, and a 7 mm margin around the vessels should be maintained, excluding bone, bowel, or muscle, including suspicious lymph nodes, lymphoceles, and pertinent surgical clips.

To cover the presacral region, the volumes on each side of the pelvis should be connected with a 10-mm strip over the anterior sacrum on the level of S1-S2.

Vaginal cuff tissue frequently extends superior to the vaginal marker, and this tissue should be included in the CTV. The volume of tissue to be included above the vaginal marker will depend on the location of adjacent small bowel (that will not be considered CTV) and the amount of soft tissue present. Generally, the minimum tissue to be included above the vaginal marker or vaginal cuff tissue is 1 cm. At the inferior portion of the CTV the internal iliac vessels may become poorly

visualized. In this case, the CTV should be bounded posteriorly by the piriformis muscle, even if the CTV extends more than 7 mm beyond visible vasculature.
Ensure the lateral border of the volume extends to the psoas muscle and pelvic sidewall. Continue the medial border around the external iliac vessels posteriorly, parallel to the sidewall, until it joins the medial contour of the internal iliac vessels to encompass the obturator region. This creates a strip medial to the pelvic sidewall that should be at least 17 mm wide.
The distal lateral external iliac nodes should be encompassed if there is other iliac lymph node involvement, and in that case the contour should be extended around the external iliac artery anterolaterally along the iliopsoas muscle by an additional 10 mm.

A uniform 3-dimensional CTV expansion of 7-10 mm is used to obtain the PTV. Because of internal organ movement due to changes in bladder and rectum filling the CTV-PTV margin for the upper vaginal region should be 12mm anterior-posterior and superior-inferior.
An alternative way to account for internal organ movement due to changes in bladder filling is to obtain planning CT scans both with a full and empty bladder. The vaginal/parametrial CTV should be outlined on both CT scans and these volumes should then be merged to form an integrated target volume (ITV). This ITV should be used for treatment planning to account for daily variations in the location of the vaginal cuff related to variation in bladder filling.

It is recommended to use a protocol for standard bladder filling during RT. In case of huge rectal filling at the planning CT scan, a repeat scan is recommended after a few days (and after using laxatives as appropriate).

Radiotherapy Quality Assurance

Radiotherapy quality control for the PORTEC-3 trial will be conducted by the Trans Tasman Radiation Oncology Group (TROG; where the trial is known as “TROG 08.04”), in liaison with ANZGOG and in close collaboration with the lead group and other participating groups.
In accordance with TROG policy, a technical review will be conducted for this study. Remote technical audits will be conducted by an independent reviewer. All Radiation Oncology centers participating in this study will be required to submit a recent case for review, and further audits will be undertaken of 1 in every 5 cases or at least once a year for each centre. Guidelines associated with this review process are contained within the TROG documentation relevant to this study.

Copies of CRF will be used to confirm compliance with the protocol treatment requirements; if needed copies of source documents will be used to verify specific information.

A checklist of information required for each RT QA case will be provided by the TROG QA Office. Review material will be required within four weeks of QA request. Information from the CRF required for review will include:

- Treatment prescription (including total dose and number of fractions)
- Daily dose record (including dates of treatment delivery)
- Radiotherapy treatment planning (RTP) electronic data file exported in DICOM RT or RTOG format for SWAN review
- Documentation to verify the dates of portal images taken during radiotherapy

The TROG QA Office will coordinate the review in consultation with the TROG Study PI and report to the Trial Management Committee. Results will be reported to the TMC at least 6 monthly and to the TROG Scientific Committee biannually.

All queries relating to the radiotherapy guidelines and quality assurance procedures should be directed to the TROG Study Chair.

TROG Study Chair: Dr Pearly Khaw
Division of Radiation Oncology
Peter MacCallum Cancer Centre
Locked Bag 1
A'Beckett Street VIC 8006
Tel: +61 3 96561111
Fax: +61 3 96561424
Email: pearly.khaw@petermac.org

APPENDIX F. COMMON TERMINOLOGY CRITERIA FOR ADVERSE EVENTS

The National Cancer Institute Common Terminology Criteria for Adverse Events (CTCAE) v3.0 will be used for scoring of adverse events. The CTCAE v3.0 Document (pdf file), Index, Instructions and Guidelines, FAQ, Mapping documents and other tools can be downloaded from http://ctep.cancer.gov/reporting/ctcnew.html

The CTC website http://ctep.cancer.gov/reporting/ctc.html also includes the CTCAE Dictionary, a web-based application which assists in locating appropriate adverse event terms from CTCAE v3.0, and “Responsible Adverse Event (AE) Reporting: Finding Appropriate AE Terms”, a Power Point slide presentation which provides an overview of AE related information and illustrates the search capabilities of the tools available from the CTCAE v3.0 website.

The adverse event (AE) grade refers to its severity. The CTCAE v3.0 displays Grades 1 through 5 with unique clinical descriptions of severity for each AE, based on this general guideline:

Grade 1 Mild AE
Grade 2 Moderate AE
Grade 3 Severe AE
Grade 4 Life-threatening or disabling AE
Grade 5 Death related to AE

Common Terminology Criteria for Adverse Events v3.0 (CTCAE)

Publish Date: August 9, 2006
Full CTCAE available at http://ctep.cancer.gov/forms/ctcaev3

Grade	1	2	3	4	5
	Mild	Moderate	Severe	Life-threatening	Death
**Allergy**					
Allergic reaction/ Hypersensitivity (including drug fever)	Transient flushing or rash; drug fever <38° C (<100.4° F)	Rash; flushing; urticaria; dyspnea; drug fever ≥38°C (≥100.4° F)	Symptomatic bronchospasm, with or without urticaria; parenteral medication(s) indicated; allergy related edema/angioedema; hypotension	Anaphylaxis	Death
Allergy/Immunology, other, specify	Mild	Moderate	Severe	Life-threatening; disabling	Death
**Auditory/hearing**					
Auditory/hearing	-	Hearing loss not requiring hearing aid or intervention (i.e. not interfering with ADL)	Hearing loss requiring hearing aid or intervention (i.e. interfering with ADL)	Profound bilateral hearing loss (>90 dB)	-
**Cardiovascular (general)**					
Edema	Mild	Moderate	Severe	Life-threatening; disabling	Death`

Hypertension	Asymptomatic, transient (<24 hrs) increase by >20 mmHG (diastolic) or to >150/100 if previously WNL; intervention not indicated	Recurrent or persistent (≥24 hrs) or symptomatic increase by >20 mmHg (diastolic) or to >150/100 if previously WNL; monotherapy may be indicated	Requiring more than one drug or more intensive therapy than previously	Life-threatening consequences (e.g. hypertensive crisis)	Death
Hypotension	Changes, intervention not indicated	Brief (<24 hrs) fluid replacement or other therapy; no physiologic consequences	Sustained (≥24 hrs) therapy, resolves without persisting physiologic consequences	Shock (e.g. acidemia; impairment of vital organ function)	Death
Cardiac ischemia/infarction	Asymptomatic arterial narrowing without ischemia	Asymptomatic and testing suggesting ischemia; stable angina	Symptomatic and testing consistent with ischemia; unstable angina; intervention indicated	Acute myocardial infarction	Death
Cardiac left ventricular failure	Mild	Moderate	Severe	Life-threatening; disabling	Death
Cardiovascular general – other, specify	Mild	Moderate	Severe	Life-threatening; disabling	Death
**Dermatological/ skin**					
Alopecia (hair loss)	Thinning or patchy	Complete	-	-	-
**Grade**	**1**	**2**	**3**	**4**	**5**
Injection site reaction; extravasation changes	Pain; itching; erythema	Pain or swelling, with inflammation or phlebitis	Ulceration or necrosis that is severe; operative intervention indicated	-	-
Radiation dermatitis	Faint erythema or dry desquamation	Moderate to brisk erythema; patchy moist desquamation, mostly confined to skin folds and creases; moderate edema	Moist desquamation other than skin folds or creases; bleeding induced by minor trauma or abrasion	Skin necrosis or ulceration or full thickness dermis; spontaneous bleeding from involved site	Death
**Gastro-Intestinal**					
Anorexia	Loss of appetite without alteration in eating habits	Oral intake altered without significant weight loss or malnutrition; oral nutritional supplements indicated	Associated with significant weight loss or malnutrition (e.g. inadequate oral caloric and/or fluid intake); IV fluids, tube feedings or TPN indicated	Life-threatening consequences	Death
Constipation	Occasional or intermittent symptoms; occasional use of stool softeners, laxatives, dietary modification or enema	Persistent symptoms with regular use of laxatives or enemas indicated	Symptoms interfering with ADL; obstipation with manual evacuation indicated	Life-threatening consequences (e.g. obstruction, toxic megacolon)	Death
Dehydration	Increased oral fluids indicated; dry mucous membranes; diminished skin turgor	IV fluids indicated <24 hrs	IV fluids indicated ≥24 hrs	Life-threatening consequences (e.g. haemodynamic collapse)	Death
Diarrhoea	Increase of <4 stools per day over baseline; mild increase in ostomy output compared to baseline	Increase of 4-6 stools per day over baseline; IV fluids indicated <24 hrs; moderate increase in ostomy output compared to baseline; not interfering with ADL	Increase of ≥7 stools per day over baseline; incontinence; IV fluids ≥24 hrs; hospitalisation; severe increase in ostomy output compared to baseline; interfering with ADL	Life-threatening circumstances (e.g. haemodynamic collapse)	Death
Ileus, GI (functional obstruction of bowel, i.e. neuroconstipation)	Asymptomatic, radiographic findings only	Symptomatic; altered GI function (e.g. altered dietary habits); IV fluids indicated <24hrs	Symptomatic and severely altered GI function; IV fluids, tube feeding, or TPN indicated ≥24hrs	Life-threatening consequences	Death
Nausea	Loss of appetite without alteration in eating habits	Oral intake decreased without significant weight loss, dehydration or malnutrition; IV fluids indicated <24hrs	Inadequate oral caloric or fluid intake; IV fluids, tube feedings or TPN indicated ≥24 hrs	Life threatening consequences	Death
Proctitis	Rectal discomfort, intervention not indicated	Symptoms not interfering with ADL; medical intervention indicated	Stool incontinence or other symptoms interfering with ADL; operative intervention indicated	Life-threatening consequences (e.g. perforation)	Death

Stomatitis/ pharyngitis	Mild	Moderate	Severe	Life-threatening; disabling	Death
**Grade**	**1**	**2**	**3**	**4**	**5**
Vomiting	1 episode in 24 hrs	2-5 episodes in 24 hrs; IV fluids indicated <24 hours	≥ 6 episodes in 24 hrsl; IV fluids or TPN indicated ≥ 24hrs	Life-threatening consequences	Death
Gastrointestinal – other, specify	Mild	Moderate	Severe	Life-threatening; disabling	Death
**Haematological**					
Haemoglobin	<LLN – 10.0 g/dL<br><LLN – 6.2 mmol/L<br><LLN – 100 g/L	<10.0 – 8.0 g/dL<br><6.2 – 4.9 mmol/L<br><100 – 80 g/L	<8.0 – 6.5 g/dL<br><4.9 – 4.0 mmol/L<br><80-65 g/L	<6.5 g/dL<br><4.0 mmol/L<br><65 g/L	Death
Leukocytes / WBC	<LLN – 3000/mm^3<br><LLN – 3.0 x 10^9 /L	<3000 – 2000/mm^3<br><3.0 – 2.0 x 10^9 /L	<2000 – 1000/mm^3<br><2.0 – 1.0 x 10^9 /L	<1000/mm^3<br><1.0 x 10^9 /L	Death
Lymphocytes	<LLN – 800/mm^3<br><LLN – 0.8 x 10^9 /L	<800 – 500/mm^3<br><0.8 – 0.5 x 10^9 /L	<500 – 200/mm^3<br><0.5 – 0.2 x 10^9 /L	<200/mm^3<br><0.2 x 10^9 /L	Death
Neutrophils/ granulocytes (ANC/AGC)	<LLN – 1500/mm^3<br><LLN – 1.5 x 10^9 /L	<1500 – 1000/mm^3<br><1.5 – 1.0 x 10^9 /L	<1000 – 500/mm^3<br><1.0 – 0.5 x 10^9 /L	<500/mm^3<br><0.5 x 10^9 /L	Death
Platelets	LLN – 75,000/mm^3<br><LLN – 75.0 x 10^9 /L	<75,000 – 50,000/mm^3<br><75.0 – 50.0 x 10^9/L	<50,000 – 25/000/mm^3<br><50.0 – 25.0 x 10^9/L	<25,000/mm^3<br><25.0 x 10^9 /L	Death
**Infection**					
Febrile neutropenia – fever without clinically or microbiologically documented infection (ANC <1.0 x 10^9/L fever ≥ 38.5^o C)	-	-	Present	Life-threatening consequences (e.g. septic shock, hypotension, acidosis, necrosis)	Death
Infection with neutropenia Documented infection with grade 3 or 4 neutrophils (ANC <1.0 x 10^9/L)	-	Localized, local intervention indicated	IV antibiotic, antifungal or antiviral intervention indicated; interventional radiology or operative intervention indicated	Life-threatening consequences (e.g. septic shock, hypotension, acidosis, necrosis)	Death
Infection without neutropenia Infection with normal ANC or Grade 1-2 neutrophils	-	Localized, local intervention indicated	IV antibiotic, antifungal or antiviral intervention indicated; interventional radiology or operative intervention indicated	Life-threatening consequences (e.g. septic shock, hypotension, acidosis, necrosis)	Death
**Lymphatics**					
Lymphatics	Mild	Moderate	Severe	Life-threatening; disabling	Death
**Neurological**					
Neuropathy – Motor	Asymptomatic, weakness on exam/testing only	Symptomatic weakness interfering with function, but not interfering with ADL	Weakness interfering with ADL; bracing or assistance to walk (e.g. cane or walker) indicated	Life-threatening; disabling (e.g. paralysis)	Death
**Grade**	**1**	**2**	**3**	**4**	**5**
Neuropathy - sensory	Asymptomatic; loss of deep tendon reflexes or paresthesia (including tingling) but not interfering with function	Sensory alteration or paresthesia (including tingling), interfering with function, but not interfering with ADL	Sensory alteration or paresthesia interfering with ADL	Disabling	Death
Neuropathy – other, specify	Mild	Moderate	Severe	Life threatening; disabling	Death
**Pain**					

Arthralgia (joint paint)	Mild pain not interfering with function	Moderate pain; pain or analgesics interfering with function, but not interfering with ADL	Severe pain; pain or analgesics severely interfering with ADL	Disabling	-
Dyspareunia (coital pain)	Mild pain not interfering with function	Moderate pain; pain or analgesics interfering with function, but not interfering with ADL	Severe pain; pain or analgesics severely interfering with ADL	Disabling	-
Myalgia (muscle pain)	Mild pain not interfering with function	Moderate pain; pain or analgesics interfering with function, but not interfering with ADL	Severe pain; pain or analgesics severely interfering with ADL	Disabling	-
Pain – other, Specify	Mild pain not interfering with function	Moderate pain; pain or analgesics interfering with function, but not interfering with ADL	Severe pain; pain or analgesics severely interfering with ADL	Disabling	-
**Pulmonary/ Upper Respiratory**					
Dyspnoea (shortness of breath)	Dyspnea on exertion, but can walk 1 flight of stairs without stopping	Dyspnea on exertion but unable to walk 1 flight of stairs or 1 city block (0.1km) without stopping	Dyspnea with ADL	Dyspnea at rest; intubation/ventilator indicated	Death
Pulmonary/ Upper Respiratory – other: specify	Mild	Moderate	Severe	Life-threatening; disabling	Death
**Musculoskeletal**					
Osteonecrosis	Asymptomatic, radiographic findings only	Symptomatic and interfering with function, but not interfering with ADL; minimal bone removal indicated (i.e. minor sequestrectomy)	Symptomatic and interfering with ADL; operative intervention or hyperbaric oxygen indicated	Disabling	Death
Musculoskeletal – other, specify	Mild	Moderate	Severe	Life-threatening; disabling	Death
**Renal/ Genitourinary**					
Bladder spasms	Symptomatic, intervention not indicated	Symptomatic, antispasmodics indicated	Narcotics indicated	Major surgical intervention indicated (e.g. cystectomy)	-
**Grade**	**1**	**2**	**3**	**4**	**5**
Creatinine	>ULN – 1.5xULN	>1.5 – 3.0 ULN	>3.0 - 6.0 x ULN	> 6.0 x ULN	Death
Renal failure	-	-	Chronic dialysis not indicated	Chronic dialysis or renal transplant indicated	Death
Dysuria	Mild pain not interfering with function	Moderate pain; pain or analgesics interfering with function, but not interfering with ADL	Severe pain; pain or analgesics severely interfering with ADL	Disabling	
Incontinence, urinary	Occasional (e.g. with coughing, sneezing etc), pads not indicated	Spontaneous, pads indicated	Interfering with ADL; intervention indicated (e.g. clamp, collagen injections)	Operative intervention indicated (e.g. cystectomy or permanent urinary diversion	-
Urinary frequency/urgency	Increase in frequency or nocturia up to 2 x normal; enuresis	Increase >2 x normal but <hourly	≥1 x/hr; catheter indicated	-	-
Renal/ Genitourinary – other, specify	Mild	Moderate	Severe	Life-threatening; disabling	Death
**Sexual/ Reproductive**					
Libido	Decrease in interest but not affecting relationship; intervention not indicated	Decrease in interest and adversely affecting relationship; intervention indicated	-	-	-
Vaginal dryness	Mild	Interfering with sexual function; dyspareunia; intervention indicated	-	-	-
**Other Toxicity**					
Specify	Mild	Moderate	Severe	Life-threatening; disabling	Death

APPENDIX G. QUALITY OF LIFE QUESTIONNAIRE – *please note: Dutch, French, Italian and German QoL versions can be downloaded from* www.clinicalresearch.nl/portec3

EORTC QLQ-C30 (version 3)

We are interested in some things about you and your health. Please answer all of the questions yourself by circling the number that best applies to you. There are no "right" or "wrong" answers. The information that you provide will remain strictly confidential.

Please fill in your first initial: |___|
Your birth date (Day, Month, Year): |__|__||__|__||__|__|__|__|
Today's date (Day, Month, Year): |__|__||__|__||__|__|__|__|

	Not at All	A Little	Quite a Bit	Very Much
1. Do you have any trouble doing strenuous activities, like carrying a heavy shopping bag or a suitcase?	1	2	3	4
2. Do you have any trouble taking a long walk?	1	2	3	4
3. Do you have any trouble taking a short walk outside of the house?	1	2	3	4
4. Do you need to stay in bed or a chair during the day?	1	2	3	4
5. Do you need help with eating, dressing, washing yourself or using the toilet?	1	2	3	4

**During the past week:**	Not at All	A Little	Quite a Bit	Very Much
6. Were you limited in doing either your work or other daily activities?	1	2	3	4
7. Were you limited in pursuing your hobbies or other leisure time activities?	1	2	3	4
8. Were you short of breath?	1	2	3	4
9. Have you had pain?	1	2	3	4
10. Did you need to rest?	1	2	3	4
11. Have you had trouble sleeping?	1	2	3	4
12. Have you felt weak?	1	2	3	4
13. Have you lacked appetite?	1	2	3	4
14. Have you felt nauseated?	1	2	3	4
15. Have you vomited?	1	2	3	4

Please go on to the next page

**During the past week:**	**Not at All**	**A Little**	**Quite a Bit**	**Very Much**
16. Have you been constipated?	1	2	3	4
17. Have you had diarrhea?	1	2	3	4
18. Were you tired?	1	2	3	4
19. Did pain interfere with your daily activities?	1	2	3	4
20. Have you had difficulty in concentrating on things, like reading a newspaper or watching television?	1	2	3	4
21. Did you feel tense?	1	2	3	4
22. Did you worry?	1	2	3	4
23. Did you feel irritable?	1	2	3	4
24. Did you feel depressed?	1	2	3	4
25. Have you had difficulty remembering things?	1	2	3	4
26. Has your physical condition or medical treatment interfered with your family life?	1	2	3	4
27. Has your physical condition or medical treatment interfered with your social activities?	1	2	3	4
28. Has your physical condition or medical treatment caused you financial difficulties?	1	2	3	4

For the following questions please circle the number between 1 and 7 that best applies to you:

29. How would you rate your overall health during the past week?

1 2 3 4 5 6 7

Very poor Excellent

30. How would you rate your overall quality of life during the past week?

1 2 3 4 5 6 7

Very poor Excellent

© Copyright 1995 EORTC Quality of Life Group. All rights reserved. Version 3.0

EORTC QLQ – CX24

Patients sometimes report that they have the following symptoms or problems. Please indicate the extent to which you have experienced these symptoms or problems, please answer by circling the number that best applies to you.

During the past week:	Not at all	A little	Quite a bit	Very much
31. Have you had cramps in your abdomen?	1	2	3	4
32. Have you had difficulty in controlling your bowels?	1	2	3	4
33. Have you had blood in your stools (motions)?	1	2	3	4
34. Did you pass water/urine frequently?	1	2	3	4
35. Have you had pain or a burning feeling when urinating?	1	2	3	4
36. Have you had leaking of urine?	1	2	3	4
37. Have you had difficulty emptying your bladder?	1	2	3	4
38. Have you had swelling in one or both legs?	1	2	3	4
39. Have you had pain in your lower back?	1	2	3	4
40. Have you had tingling or numbness in your hands or feet?	1	2	3	4
41. Have you had irritation or soreness in your vagina or vulva?	1	2	3	4
42. Have you had discharge from your vagina?	1	2	3	4
43. Have you had abnormal bleeding from your vagina?	1	2	3	4
44. Have you had hot flushes and/or sweats?	1	2	3	4
45. Have you felt physically less attractive as a result of your disease or treatment?	1	2	3	4
46. Have you felt less feminine as a result of your disease or treatment?	1	2	3	4
47. Have you felt dissatisfied with your body?	1	2	3	4

Please go on to the next page

**During the past 4 weeks:**	**Not at all**	**A little**	**Quite a bit**	**Very much**
48. Have you worried that sex would be painful?	1	2	3	4
49. Have you been sexually active?	1	2	3	4

**Answer these questions only if you have been sexually active during the past <u>4 weeks</u>:**	**Not at all**	**A little**	**Quite a bit**	**Very much**
50. Has your vagina felt dry during sexual activity?	1	2	3	4
51. Has your vagina felt short?	1	2	3	4
52. Has your vagina felt tight?	1	2	3	4
53. Have you had pain during sexual intercourse or other sexual activity?	1	2	3	4
54. Was sexual activity enjoyable for you?	1	2	3	4

<u>EORTC QLQ - OV28 (subscale)</u>

**During the past week:**	**Not at All**	**A Little**	**Quite a Bit**	**Very Much**
55. Did you have a bloated feeling in your abdomen / stomach?	1	2	3	4
56. Were you troubled by passing wind / gas / flatulence?	1	2	3	4
57. Have you lost any hair?	1	2	3	4
58. Answer this question only if you had any hair loss: Were you upset by the loss of your hair?	1	2	3	4
59. Did food and drink taste different from usual?	1	2	3	4
60. Have you had tingling hands or feet?	1	2	3	4
61. Have you had numbness in your fingers or toes?	1	2	3	4
62. Have you felt weak in your arms or legs?	1	2	3	4
63. Did you have aches or pains in your muscles or joints?	1	2	3	4
64. Did you have problems with hearing?	1	2	3	4

© Copyright EORTC Quality of Life Group. All rights reserved

APPENDIX H. PATIENT INFORMATION (NETHERLANDS)

Patienteninformatie ten behoeve van een wetenschappelijk onderzoek:

PORTEC-3, een onderzoek naar de toevoeging van chemotherapie tijdens en na de uitwendige bestraling bij patiënten met baarmoederkanker (endometriumcarcinoom).

Geachte mevrouw,

U bent gevraagd om deel te nemen aan een wetenschappelijk onderzoek. U hebt hierover een gesprek met uw arts gehad. Deze schriftelijke informatie is bedoeld als herhaling en aanvulling op wat uw arts heeft uitgelegd. Voor u een beslissing neemt is het belangrijk dat u deze informatie goed leest.

Wat is het doel van de studie?

Kort geleden bent u geopereerd door de gynaecoloog voor baarmoederkanker (endometriumcarcinoom). Bij deze operatie zijn in ieder geval uw baarmoeder en eierstokken verwijderd, met als eindresultaat dat alle zichtbare kanker is weggenomen.
We weten dat sommige patiënten een wat hoger risico hebben dat de kanker op een later moment terug komt. Bij het weefselonderzoek na de operatie is gebleken dat u bij deze groep hoort.
Uit eerder onderzoek is bekend dat de kans op terugkeer van kanker wordt verminderd door radiotherapie (bestraling), en uw arts heeft u daarom verwezen naar de radiotherapeut-oncoloog.

De standaard behandeling bestaat uit een serie uitwendige bestralingen op het bekkengebied. Toevoeging van chemotherapie tijdens de bestraling maakt deze behandeling waarschijnlijk nog effectiever. Daarnaast kan chemotherapie mogelijke tumorcellen buiten het bekken, die niet in het bestralingsgebied liggen, bestrijden.
Uit studies bij andere soorten kanker is gebleken dat chemotherapie het optreden van uitzaaiingen kan voorkomen. Ook voor baarmoederkanker zijn er eerste aanwijzingen hiervoor. Daarom hebben de meeste Nederlandse centra (bestralingsafdelingen, afdelingen gynaecologie en interne oncologie) besloten hun patiënten te vragen mee te werken aan dit onderzoek.

Het doel van de studie is te onderzoeken of de toevoeging van chemotherapie tijdens en na bestraling de overlevingskansen verbetert en de kans op uitzaaiingen verkleint. Hierbij wordt gelet op de resultaten van de behandeling (een zo laag mogelijke kans op terugkeer van de ziekte), maar ook op de bijwerkingen van de behandelingen en de “kwaliteit van leven” die u ervaart.
Het betreft een fase 3 (vergelijkend) onderzoek.

Wat betekent de studie voor mij?

Als u besluit mee te doen aan het onderzoek, wordt door middel van loting bepaald welke behandeling u krijgt:

1. een serie van 27 uitwendige bestralingen (standaard behandeling), óf
2. dezelfde bestralingen, gecombineerd met 2 kuren chemotherapie tijdens de bestraling, en 4 kuren chemotherapie na de bestraling, telkens om de 3 weken.

Deze loting (randomisatie) is om twee gelijke groepen te krijgen, wat nodig is om de uitkomst van de behandelingen later goed te kunnen vergelijken. Noch uzelf, noch uw artsen kunnen bepalen welke van de twee behandelingen u krijgt.

Wat houdt bestraling in?

De bestraling is de standaard behandeling voor baarmoederkanker in uw situatie. De bestraling zelf maakt dus *geen* deel uit van het onderzoek, en blijft hetzelfde of u wel of niet aan het onderzoek deelneemt.
U krijgt een serie van 27 bestralingen, die dagelijks worden gegeven (op werkdagen, dus 5x per week). Deze bestralingsbehandeling wordt poliklinisch uitgevoerd en duurt per dag zo’n 10-15 minuten.
Bij sommige patiënten wordt na de uitwendige bestraling ook inwendige bestraling gegeven, in 1 tot 3 behandelingen. Hierbij wordt het gebied rond het bovenste gedeelte van de vagina van binnenuit bestraald via een gladde cilinder. Voor het inbrengen hiervan is geen narcose nodig. U voelt van de bestraling niets. Zodra de bestraling klaar is wordt de cilinder verwijderd. De inwendige bestraling wordt poliklinisch gegeven, en u kunt direct erna naar huis.

Wat zijn de bijwerkingen van de bestraling?

Tijdens de serie uitwendige bestralingen zullen geleidelijk bijwerkingen gaan optreden, doorgaans vanaf de 2e-3e bestralingsweek. Klachten die *kunnen* optreden zijn:

- vermoeidheid
- vaker en sterkere aandrang voor ontlasting
- zachtere ontlasting tot diarree
- krampen in de buik
- vaker aandrang voor plassen met soms branderigheid bij het plassen
- irritatie van het slijmvlies van uw vagina.

De bijwerkingen zijn vooral aanwezig tijdens de 2 laatste bestralingsweken en de eerste 2 weken na afloop van de bestraling. Of en hoe sterk deze bijwerkingen optreden verschilt sterk van persoon tot persoon.
De klachten van de darmen komen het meest voor. U krijgt dieetadviezen en zonodig medicijnen tegen diarree. Deze dieetadviezen bestaan vooral uit het vermijden van sterk gekruid en vet voedsel, en gasvormende spijzen zoals ui en prei.
Vanaf 1-2 weken na het einde van de bestraling zullen de klachten verminderen, en doorgaans kunt u na enkele weken zonder problemen uw normale dieet hervatten. Een kleine groep patiënten houdt langere tijd last van diarree met sterke aandrang. Ook vermoeidheid kan bij sommige patiënten langer aanhouden.

Wat houdt chemotherapie in?

Tijdens de bestraling worden 2 kuren cisplatine gegeven, en na de bestraling 4 kuren carboplatine en paclitaxel (ook ‘taxol’ genoemd). Voor iedere kuur wordt d.m.v. bloedonderzoek (en eventueel urine onderzoek) gecontroleerd of uw bloedaanmaak en nierfunctie voldoende zijn voor het krijgen van de chemotherapie.

1. *Chemotherapie tijdens de bestraling.*

Tijdens de bestraling bestaat de chemotherapie uit het middel cisplatine, dat in de 1e en 4e week van de bestraling in een infuus wordt gegeven. Dit gebeurt tijdens een dagopname, of een korte opname waarbij u 1 nacht in het ziekenhuis blijft. Voor en na het cisplatine infuus krijgt u een infuus met vocht, en ook krijgt u middelen tegen misselijkheid en braken in het infuus toegediend. De bestraling wordt op die dag meestal één tot enkele uren na de toediening van de chemotherapie gegeven.

2. *Chemotherapie na de bestraling*.

Na het einde van de bestraling bestaat de chemotherapie uit een combinatie van 2 middelen: carboplatine en paclitaxel. Hiervan worden 4 kuren gegeven, telkens met 3 weken ertussen. Deze kuren worden poliklinisch gegeven op een afdeling voor dagbehandeling. De middelen worden via een infuus toegediend, dit duurt enkele uren. Ook bij deze kuren krijgt u medicijnen om misselijkheid en braken te onderdrukken.

Wat zijn de bijwerkingen van chemotherapie?

1. *Cisplatine, 2 kuren tijdens de bestraling.*

Bijwerkingen van cisplatine zijn misselijkheid en braken, daarom krijgt u medicijnen om dit te voorkomen toegediend in het infuus. Ook voor thuis krijgt u een recept voor medicijnen mee. Meestal zijn de klachten mild. Als misselijkheid optreedt is dit meestal gedurende de eerste 3 dagen na de kuur.
Cisplatine kan nierschade geven. Om dit te voorkomen krijgt u voor en na cisplatine via een infuus vocht toegediend. Hiervoor wordt u gedurende een hele dag, of soms een dag en nacht, opgenomen op de dagbehandeling of verpleegafdeling.
Ook thuis is een goede vochtinname belangrijk, zeker als er ook diarree is ontstaan of als u heeft gebraakt. Het is daarom van belang dat u ook thuis goed blijft drinken, liefst 1½-2 liter vocht per dag. Als dit niet lukt moet u contact opnemen met uw behandelend internist-oncoloog.
Andere bijwerkingen van cisplatine (die zelden voorkomen bij de dosering die u krijgt) kunnen zijn: gehoorverlies, tintelingen of een doof gevoel in de tenen en eventueel de vingers. Van de cisplatine kuren krijgt u *geen* haaruitval.

2. *Carboplatine en paclitaxel, 4 kuren na de bestraling*

Carboplatine heeft minder bijwerkingen dan cisplatine. De kans op nierschade is veel kleiner. Misselijkheid en braken treden minder vaak op, terwijl ook gehoorverlies en tintelingen van de voeten en de handen slechts zelden voorkomen.
Paclitaxel kan een allergische reactie geven, waardoor u bijvoorbeeld huiduitslag of klachten van benauwdheid en zwelling van het gezicht kan krijgen. Om deze reactie te voorkomen krijgt u vóór toediening van paclitaxel medicijnen toegediend. Ook krijgt u medicijnen tegen misselijkheid en het braken. Mogelijk ontstaat geringe diarree. Ook spierpijnen kunnen voorkomen in de eerste dagen na de kuur.

Paclitaxel kan ook -meestal tijdelijk- een doof gevoel of tintelingen in de voeten en handen geven.
Paclitaxel geeft bij alle patiënten haaruitval. Voor de behandeling krijgt u informatie over het aanvragen van een pruik. De haaruitval komt ongeveer 3 weken na de 1e kuur. Na afloop van de chemotherapie zal het ongeveer 3 weken duren voor uw haar weer begint te groeien. Het haar zal in alle gevallen terugkomen.

Cisplatine, carboplatine en paclitaxel kunnen remming van de aanmaak van witte bloedcellen, rode bloedcellen en bloedplaatjes geven. Dit kan soms leiden tot een verlaagd aantal witte bloedcellen. Omdat de witte bloedcellen zorgen voor de afweer bij infecties, is het van groot belang dat u contact opneemt met uw behandelend (of dienstdoend) internist-oncoloog als u koorts heeft boven de 38.5^{0}C tijdens of na de behandeling met chemotherapie. De arts kan dan het aantal witte bloedcellen bepalen en beoordelen of ondersteuning met antibiotica via een infuus of met tabletten noodzakelijk is.

Wat houdt “kwaliteit van leven” onderzoek in?

Het kwaliteit van leven onderzoek bestaat uit een vragenlijst, die u voorafgaand aan de behandeling, na het einde van de bestraling, en dan na 6, 12 en 18 maanden, en na 2, 3, en 5 jaar (gerekend vanaf het begin van de behandeling) invult. Met deze vragenlijsten wordt de kwaliteit van leven, zoals u dat zelf ervaart, gemeten. Uzelf kunt namelijk het beste beoordelen hoe zwaar de behandeling is en wat de gevolgen zijn voor uw welbevinden. De kwaliteit van leven vragenlijst bestaat uit een algemeen gedeelte van 30 vragen, en een specifiek gedeelte (eveneens 30 vragen). Het invullen zal ongeveer 10-15 minuten duren.

De eerste vragenlijst krijgt u van uw radiotherapeut. Daarbij zit een adresformulier met de vraag of u het goed vindt dat de volgende vragenlijsten rechtstreeks vanuit het coördinerende centrum naar uw huisadres worden gestuurd. Indien u daarin toestemt, stuurt u de lijst met uw adresgegevens terug. U krijgt dan de volgende keren de vragenlijst thuis toegestuurd, en u kunt deze terugsturen in een gratis antwoordenveloppe.
De naam- en adresgegevens die u voor het kwaliteit van leven onderzoek invult, worden in een apart computerbestand bewaard dat *alleen* voor het toezenden van de vragenlijsten gebruikt wordt. Dit bestand maakt geen deel uit van de overige (gecodeerde) gegevens die voor het onderzoek worden bewaard.
De antwoorden op de vragenlijsten worden i.v.m. de privacy met grote zorg behandeld. Bij ontvangst van de lijsten worden de gegevens anoniem gemaakt en onder code bewaard, zodat ze later bij de bewerking niet herleidbaar zijn.
Voor de studie is het van groot belang dat u iedere lijst invult en opstuurt. Als het studiecentrum ze niet van u terug ontvangt, dan wordt u opnieuw (schriftelijk) benaderd met de vraag dit alsnog te doen. Als u liever niet meer mee wilt werken, staat het u uiteraard altijd vrij zich terug te trekken. Dit kunt u dan op het formulier aangeven.

Wat gebeurt er na de behandeling?

Na de behandeling zult u om en om controle afspraken krijgen bij uw gynaecoloog en uw radiotherapeut, en indien nodig ook bij de medisch oncoloog. De eerste twee jaar zullen deze controles om de 3 maanden zijn, daarna om de 6 maanden, en na 5 jaar ieder jaar.
De controle bestaat vooral uit het informeren hoe het met u gaat en of u klachten heeft, en lichamelijk onderzoek (onderzoek van de buik en inwendig onderzoek). Eén keer per jaar wordt bloed geprikt en een longfoto gemaakt. Dit zijn onderzoeken die ook plaatsvinden als u niet aan de studie meedoet, alhoewel niet alle centra jaarlijks bloedonderzoek en een longfoto zouden doen. Andere onderzoeken, zoals een CT scan, worden alleen gedaan als daar een aanleiding voor is.

Moet ik aan de studie meedoen?

Nee. Uw beslist zelf of u meedoet of niet. Als u besluit mee te doen, wordt u gevraagd het toestemmingsformulier in te vullen. U houdt altijd de vrijheid om op die beslissing terug te komen. U hoeft hiervoor geen verklaring te geven. Het wel of niet meedoen heeft op geen enkele wijze gevolgen voor de verstandhouding met uw arts.
Ook uw behandelend arts kan voorstellen uw deelname aan het onderzoek te stoppen als hij of zij vindt dat dit in uw situatie beter is. Uiteraard gebeurt dit in overleg met u.
Als u besluit *niet* mee te doen aan het onderzoek, wordt in principe de standaard behandeling, uitwendige bestraling, geadviseerd.

Wat zijn de mogelijke nadelen van deelname?

Als u in de groep komt die chemotherapie krijgt, kunnen de bijwerkingen optreden die hierboven genoemd zijn. U kunt dit verder bespreken met uw arts.

Wat zijn de mogelijke voordelen van deelname?

In een eerdere studie werden aanwijzingen gevonden dat chemotherapie de genezingskans kan verbeteren. Als u in de groep komt die chemotherapie krijgt, heeft u mogelijk een grotere genezingskans, maar zeker is dit niet.

Moet ik meteen beslissen?

Neemt u rustig enige bedenktijd voordat u beslist of u meedoet of niet. U kunt deze informatie dan nog eens bespreken met uw partner, familie, huisarts of met uw gynaecoloog. Aarzel niet uw vragen met uw behandelend arts(en) te bespreken.

Worden mijn gegevens vertrouwelijk behandeld?

U kunt ervan verzekerd zijn dat alle gegevens, die tijdens het onderzoek verzameld worden, vertrouwelijk behandeld worden. Behalve uw behandelend arts(en) zullen alleen bevoegde personen die onder toezicht van de behandelend arts staan, uw gegevens kunnen inzien.
De gegevens die voor het onderzoek worden bewaard, zullen worden gecodeerd voor ze naar het centrale computerbestand worden gestuurd. Dit betekent dat de gegevens onder code worden ingestuurd en bewaard, en alleen in uw eigen ziekenhuis (bij uw arts en daartoe bevoegde personen) bekend is welke medische gegevens bij die code horen.
Naast gegevens over de bestraling en eventuele chemotherapie, eventuele bijwerkingen, en het beloop na de behandeling, zullen medische gegevens over de operatie en het weefselonderzoek van de baarmoederkanker geregistreerd worden. Een stukje van het weefsel, waarop de diagnose baarmoederkanker gesteld is, wordt ter bevestiging van de diagnose naar het centrale pathologielaboratorium van deze studie gestuurd.
Wij lichten uw huisarts in over uw deelname aan dit onderzoek.
Gegevens of resultaten met betrekking tot het onderzoek worden in anonieme vorm verwerkt. De resultaten van dit onderzoek kunnen gebruikt worden in wetenschappelijke publicaties, maar ook dan zijn uw persoonlijke gegevens niet herkenbaar.

Wordt een stukje weefsel bewaard?

Een heel klein gedeelte (1 cm^3) van het tumorweefsel zal onder code in het centrale pathologielaboratorium worden bewaard voor wetenschappelijk onderzoek.
Het betreft hier onderzoek naar nieuwe eigenschappen van het weefsel die een voorspellende waarde kunnen hebben voor het resultaat van de behandeling en/of het beloop van de ziekte. Hiermee hopen we in de toekomst de behandeling nog verder te kunnen verbeteren, en preciezer te kunnen uitmaken welke patiënten baat van de behandelingen zullen hebben.
Het weefsel zal *uitsluitend* worden gebruikt voor aanvullend wetenschappelijk onderzoek dat past binnen de vraagstelling van deze studie, of hieruit voortvloeit, en betrekking heeft op baarmoederkanker. Mocht u bezwaar hebben tegen het bewaren van het stukje weefsel, dan kunt u dit apart op de toestemmingsverklaring aangeven.

Is er een verzekering voor deze studie?

Het ziekenhuis heeft een verzekering afgesloten waaruit eventuele schade als gevolg van het onderzoek betaald kan worden. Als u vindt dat u schade heeft ondervonden als gevolg van het onderzoek waaraan u meedoet (of heeft meegedaan) kunt u het beste met uw behandelend arts of met de arts-onderzoekers (zie onder) bespreken hoe het met de verzekering is geregeld.

Waar kan ik terecht met mijn vragen?

Mocht u verdere vragen hebben over deze studie dan kunt u die stellen aan uw behandelend radiotherapeut-oncoloog, medisch oncoloog en gynaecoloog, of aan (naam en telefoonnummer locale coördinator). Landelijke contactpersonen voor deze studie zijn: Dr C.L. Creutzberg (radiotherapeut-oncoloog, tel: 071-5261990), Dr N. Ottevanger (internist-oncoloog, tel: 024-3610353), Dr H. Nijman (gynaecoloog-oncoloog, tel: 050-3613000) of Dr R. Kruitwagen (gynaecoloog-oncoloog, tel: 043-3874767).
Ook kunt u contact opnemen met een onafhankelijke arts: (naam en telefoonnummer locale onafhankelijke arts), of met Prof. Dr J.Trimbos, gynaecoloog, tel. 071-5262845. Deze artsen hebben geen direct belang bij dit onderzoek, maar zijn wel op de hoogte van de aard en inhoud ervan.
Verdere informatie kunt u ook vinden in folders van KWF Kankerbestrijding: “Baarmoederkanker”, “Radiotherapie”, “Chemotherapie” en “Onderzoek naar nieuwe behandelingen van kanker”. U krijgt deze folders van uw arts, of kunt ze aanvragen via www.kwfkankerbestrijding.nl

TOESTEMMINGSVERKLARING
voor deelname aan wetenschappelijk onderzoek

Titel van het onderzoek: "**PORTEC-3, een onderzoek naar de toevoeging van chemotherapie tijdens en na de uitwendige bestraling bij patiënten met baarmoederkanker (endometriumcarcinoom)**".

Ik ben naar tevredenheid over het onderzoek geïnformeerd. Ik heb de schriftelijke informatie goed gelezen. Ik ben in de gelegenheid geweest om vragen te stellen over het onderzoek. Mijn vragen zijn naar tevredenheid beantwoord. Ik heb goed over deelname aan het onderzoek kunnen nadenken. Ik heb het recht mijn toestemming op ieder moment weer in te trekken zonder dat ik daarvoor een reden hoef te geven.

Ik geef toestemming voor deelname aan het onderzoek.

Ik geef ook toestemming voor deelname aan het kwaliteit van leven onderzoek.

Ik geef wel/geen* toestemming voor het onder code bewaren van een heel klein stukje van het tumorweefsel voor aanvullend wetenschappelijk onderzoek in de toekomst.

** doorhalen wat niet van toepassing is*

Naam en voorletters: ……..………………………………………………………………….

Geboortedatum: ………………..…………………………………………………………………

Handtekening: ………………………………………… Datum: ……………………………….

Ondergetekende verklaart dat de hierboven genoemde persoon zowel mondeling als schriftelijk over het bovenvermelde onderzoek is geïnformeerd Hij/zij verklaart tevens dat een voortijdige beëindiging van de deelname door bovengenoemde persoon van geen enkele invloed zal zijn op de zorg die hem of haar toekomt.

Naam en voorletters: ………………………………………………………………………….

Functie: ...…………………………………………………………………………………….

Handtekening: …..…………………………………… Datum: ……………………………….

Dit formulier is bestemd voor onderzoek met meerderjarigen die wilsbekwaam zijn. Bij dit soort onderzoek moet door de betrokkenen zelf toestemming worden verleend. Het origineel dient in het medisch dossier te worden bewaard.

APPENDIX I. FORMS AND PROCEDURES FOR COLLECTING DATA

Form nr	Title	When to complete
1	Randomization Checklist	Before and at registration
2	On Study Form	Immediately after registration
3	Radiotherapy Form	After completion of radiotherapy
4	Chemotherapy Form	After each cycle of chemotherapy
5	Off Treatment Form	After completion or discontinuation of treatment
6	Toxicity Form	At baseline, completion of radiotherapy, at each chemotherapy cycle (if CTC grade $\geq$ 2), and at each follow-up
7	Follow-up Form	Every 6 months from the date of randomization until year 5; at year 7 and 10; and at recurrence
8	Recurrence Form	In case of tumor recurrence / progression
9	Serious Adverse Event Form	In case of SAE (<24 h by fax)

Table for filling out forms

	Time after date of registration/randomization					
**Form**	**Registration**	**Completion of RT**	**Each chemo cycle**	**End of treatment**	**6-monthly until 5th year**	**At year 7 and 10**
**1**	**X**					
**2**	**X**					
**3**		**X**				
**4**			**X**			
**5**				**X**		
**6**	**X**	**(X)**	**(X)**		**X**	**(X)**
**7**					**X**	**X**
**8**	**………………….. in case of recurrence ……………………..**					
**9**	**………………….. in case of SAE ……………………..**					
**QoL**	**X**	**X**		**At 6, 12, 18, 24, 36, and 60 months**		

APPENDIX J. Checklist for investigations at registration, treatment and follow-up

	**Time after date of registration/randomization**				
	**Before Registration***	**Completion of RT and/or CT**	**1st - 5th year: every 6 months**	**1st - 5th year: annually**	**5th -7th year: annually**
**Medical history**	**X**	**X**	**X**		**X**
**Physical and pelvic exam**	**X**	**X**	**X**		**X**
**Tumor status**	**X**	**X**	**X**		**X**
**Performance status**	**X**	**X**	**X**		**X**
**Weight**	**X**	**X**	**X**		
**Toxicity scoring**	**X**	**X**	**X**		
**Chest X-ray**	**X**			**X**	
**Abdominal CT**	**X**		**….. on indication ….**		
**Blood count/chemistry and CA-125**	**X**	**X**		**X**	
**Quality of Life questionnaire**	**X**	**Completion of RT**	**At 6, 12, 18, 24, 36, and 60 months from randomization (Netherlands: these will be sent directly to the patient's home address)**		

*Investigations before registration should have been done within 30 days before date of registration (or for pre-surgery CT/MRI within 21 days prior to surgery).

Checklist for investigations before, during and after chemotherapy

Parameter	Before start of chemotherapy*	Day 1 of each cycle*	3 weeks after last chemotherapy
Medical history	X	X	X
Physical examination	X	X	X
Height	X		
Weight	X	X	X
Performance status	X	X	X
ECG	X		
Audiography (if indicated)	(X)	(X)	(X)
Neurological assessment (if indicated)	(X)	(X)	(X)
Hb, platelets, WBC and differentiation*	X	X	X
Na, K, Mg, Ca, P, Alb, Bili, AF (APh), ASAT, ALAT, LD, serum creatinine*	X	X	X
CA-125	X		X
Creatinine clearance*	X	X	X
Toxicity evaluation	X	X	X

* Hematology and chemistry tests should be done within 72 h before each cycle, and be repeated within 24 h of the cycle in case of abnormal values

APPENDIX K. GROUP-SPECIFIC APPENDICES

NB. For NCIC CTG Group-Specific Appendix: see separate document

UK GROUP SPECIFIC APPENDIX – Glossary of terms

**AE**	Adverse Event
**AF**	Alkaline Phosphatase
**AFIP**	Armed Forces Institute of Pathology
**Alb**	Albumin
**ALAT**	Alanine Transaminase
**ANC**	Absolute Neutrophil Count
**ANOVA**	Analysis of Variance
**AP**	Doxorubicin and Cisplatin
**AR**	Adverse Reaction
**ASAT**	Aspartate Transaminase
**AUC**	Area Under the Curve
**AZGOG**	Australian & New Zealand Gynaecological Oncology Group
**Bili**	Bilirubin
**BSA**	Body Surface Area
**Ca**	Calcium
**C&G**	Cockcroft and Gault
**CAP**	Cyclophosphamide, Doxorubicine and Cisplatin
**CI**	Chief Investigator
**CMT**	Combined Modality Treatment
**COREC**	Central Office for Research Ethics Committee
**CRF**	Case Report Form
**CT**	Computerised Tomography
**CTA**	Clinical Trial Authorisation
**CTAAC**	Clinical Trials Advisory & Awards Committee
**CTC**	Common Toxicity Criteria
**CTCAE**	see NCI CTCAE
**CTSA**	Clinical Trial Site Agreement
**CTV**	Clinical Target Volume
**CXR**	Chest X-Ray
**DPA**	Data Protection Act
**DSMB**	Data and Safety Monitoring Board
**ECG**	Electrocardiogram
**EDTA**	Ethylene Diamine Tetra Acetate
**EORTC**	European Organisation for Research and Treatment of Cancer
**EudraCT**	European Clinical Trials Database
**FBC**	Full Blood Count
**FFS**	Failure-Free Survival
**FIGO**	International Federation of Gynaecology and Obstetrics
**GCP**	Good Clinical Practice
**G-CSF**	Granulocyte Colony Stimulating Factor
**GFR**	Glomerular Filtration Rate
**GI**	Gastrointestinal
**GOG**	Gynaecologic Oncology Group
**Gy**	Gray
**Hb**	Haemoglobin
**Ht**	Height
**HDR**	High-Dose Rate
**ICH GCP**	International Conference of Harmonisation - Good Clinical Practice
**ICRU**	International Commission on Radiation Units
**IDMC**	Independent Data Monitoring and Ethics Committee
**IMP**	Investigational Medicinal Product

**IMRT**	Intensity-Modulated Radiation Therapy
**ISRCTN**	International Standard Randomised Controlled Trial Number
**IV**	Intravenous
**K**	Potassium
**LAVH**	Laparoscopic Assisted Vaginal Hysterectomy
**LD**	Lactate Dehydrogenase
**LDR**	Low-Dose Rate
**LLN**	Lower Limit of Normal
**LN**	Lymphadenectomy
**LUMC**	Leiden University Medical Centre
**LVSI**	Lymph-Vascular Space Invasion
**ManGO**	Mario Negri Gynaecological Oncology Group
**METC**	Medical Ethics Committee (NL)
**Mg**	Magnesium
**MHRA**	Medicines and Healthcare products Regulatory Agency
**MREC**	Multicentre Research Ethics Committee
**MRI**	Magnetic Resonance Imaging
**Na**	Sodium
**NaCl**	Sodium Chloride
**NCIC**	National Cancer Institute of Canada
**NCI-CTCAE**	National Cancer Institute Common Terminology Criteria for Adverse Events
**NCRI**	National Cancer Research Institute
**NCRN**	National Cancer Research Network
**OS**	Overall Survival
**P**	Phosphate
**PA**	Pathology Assessment
**PDR**	Pulse Dose Rate
**PFS**	Progression Free Survival
**PI**	Principal Investigator
**PTV**	Planning Target Volume
**QA**	Quality Assurance
**QLQ**	Quality of Life Questionnaire
**QOL**	Quality of Life
**RT**	Radiotherapy
**RTOG**	Radiation Therapy Oncology Group
**SAE**	Severe Adverse Event
**SAR**	Serious Adverse Reaction
**SmPC**	Summary of Product Characteristics
**SSA**	Site Specific Assessment
**SUSAR**	Suspected Unexpected Serious Adverse Reaction
**TAH-BSO**	Total Abdominal Hysterectomy with Bilateral Salpingo-Oophorectomy
**TAP**	Doxorubicin, Cisplatin, Paclitaxel and Filgrastim Support
**TLH-BSO**	Total Laparoscopic Hysterectomy with Bilateral Salpingo-Oophorectomy
**TMG**	Trial Management Group
**TSC**	Trial Steering Committee
**UCL**	University College London
**U&E**	Urea and Electrolyte
**ULN**	Upper Limit of Normal
**UMC**	University Medical Centre Groningen (NL)
**WAI**	Whole Abdominal Radiotherapy
**WBC**	White Blood Cells
**WHO**	World Health Organisation
**WPI**	Whole Pelvic Radiotherapy

UK GROUP SPECIFIC APPENDIX - Surgical Management of Patients

The following surgical procedures are permitted for patients considered eligible for the PORTEC-3 trial (please see Protocol section 7.2 for further information)

TAH-BSO
Total Abdominal Hysterectomy with Bilateral Salpingo-Oophorectomy

TAH-BSO plus Lymphadenectomy
Total Abdominal Hysterectomy with Bilateral Salpingo-Oophorectomy and Lymphadenectomy

LAVH
Laparoscopic Assisted Vaginal Hysterectomy

LAVH plus Lymphadenectomy
Laparoscopic Assisted Vaginal Hysterectomy and lymphadenectomy

TLH-BSO
Total Laparoscopic Hysterectomy with Bilateral Salpingo-Oophorectomy

LAPAROSCOPIC SURGERY
Laparoscopic procedures are not permitted for Grade 3 tumors

Patients who have undergone laparoscopic surgery will only be considered eligible for the PORTEC-3 trial if they fall within the following two categories:

- During surgery converted from laparoscopic to abdominal surgery if intra-abdominal metastases are identified

  OR

- Centre uses laparoscopic surgery for high-risk disease as routine procedure after substantial experience, completion of learning curve and clinical evaluation

UK GROUP SPECIFIC APPENDIX - UK Chemotherapy Guidance

Cisplatin, Carboplatin and Paclitaxel should be given according to local practice. The pre and post hydration regimen should be at the discretion of the site or according to local policy. An example of suggested doses and schedules is outlined below. For further information, please see the Summary of Product Characteristics at ***http://emc.medicines.org.uk/***

Chemotherapy regimen	Dose	Route	Days
**Cisplatin**<br>Should be administered by IV infusion over a 1-2 hour period<br><br>***Suggested Pre-treatment***: 1000ml NaCl 0.9% over 1-hour period. (If urine output remains low, a further 500ml NaCl 0.9% over 30 minutes).<br>100ml Mannitol 10% over 10 minutes<br><br>***Treatment***: Cisplatin 50mg/m^2 in 500ml NaCl 0.9% over 60 minutes<br><br>***Suggested Post-treatment***:<br>100 ml Mannitol 10% over 10 minutes.<br>1000ml NaCl 0.9% + 20mmol MgSO4 + 20mmol KCL over a 2-hour period	50 mg/m^2	IV	1,22
**Carboplatin***<br>Should be administered by IV infusion over 30 minute period in 500ml Dextrose 5%	AUC 5 (calculated AUC)	IV	1,22,43,64
**Paclitaxel**<br>Should be administered by IV infusion over a 3-hour period in 500ml NaCl 0.9%<br><br>***Suggested Pre-treatment 30 minutes prior to Paclitaxel***:<br>Dexamethasone 20mg IV<br>Chlorphenamine 10mg IV<br>Ranitidine 50mg IV (over at least 2 minutes)	175mg/m^2	IV	1,22,43,64

**** The dose calculation of Carboplatin in mg is calculated according to the following Calvert formula:***

Dose (mg) = target AUC (mg/ml x min) x [GFR ml/min + 25]

NB. ***EDTA or the Cockcroft and Gault formula should be used to calculate GFR. Please see Group Specific Appendix for calculation of GFR by Cockcroft and Gault formula***

Anti-emetic therapy – Please see section Protocol section 7.4.3 for suggested pre-and post-anti-emetic therapy

UK GROUP SPECIFIC APPENDIX
Cockcroft and Gault Formula for Calculating Glomerular Filtration Rate

---

GFR – Glomerular Filtration Rate (ml/min) =

If creatinine measured in μmol/l:

Males:

$$\frac{1.23 \times (140 - \text{age}) \times \text{weight (kg)}}{\text{Serum creatinine } (\mu\text{mol/l})}$$

Females:

$$\frac{1.05 \times (140 - \text{age}) \times \text{weight (kg)}}{\text{Serum creatinine } (\mu\text{mol/l})}$$

If creatinine measured in mg/%:

Males:

$$\frac{(140 - \text{age}) \times \text{wt (kg)}}{72 \times \text{serum creatinine}}$$

Females:

$$\frac{(140 - \text{age}) \times \text{wt (kg)} \times 0.85}{72 \times \text{serum creatinine}}$$

Calculating Carboplatin Dose

Total Dose (mg) = target AUC (mg/ml x min) x [GFR ml/min + 25]

UK GROUP SPECIFIC APPENDIX
Pharmacovigilance - Definitions: ICH GCP E6

Adverse Event (AE): any untoward medical occurrence in a patient or clinical trial subject administered a medicinal product and which does not necessarily have a causal relationship with this treatment. *An AE can therefore be any unfavourable and unintended sign (including an abnormal laboratory finding), symptom, or disease temporally associated with the use of an investigational medicinal product (IMP), whether or not considered related to the IMP.*

Adverse Reaction (AR): all untoward and unintended responses to an IMP related to any dose administered. *All AE's judged by either the reporting investigator or the sponsor as having reasonable causal relationship to a medicinal product qualify as adverse reactions. The expression reasonable causal relationship means to convey in general that there is evidence or argument to suggest a causal relationship.*

Serious Adverse Event (SAE) or Serious Adverse Reaction (SAR): any untoward medical occurrence or effect that at any dose:

- results in death (*during treatment with, and for 30 days after stopping study treatment).*
- is life-threatening – *refers to an event in which the subject was at risk of death at the time of the event; it does not refer to an event which* ***hypothetically*** *might have caused death if it were more severe.*
- requires hospitalisation, or prolongation of existing inpatients' hospitalization (*hospitilisation for elective treatment of a pre-existing condition is not included*).
- results in persistent or significant disability or incapacity.
- is a congenital anomaly or birth defect.
- an important medical event.

Important medical events are those which may not be immediately life threatening, but are clearly of major clinical significance. They may jeopardise the patient and may require intervention to prevent one of the other serious outcomes.

Medical judgement should be exercised in deciding whether an AE/AR is serious in other situations. Important AE/AR's that are not immediately life-threatening or do not result in death or hospitalisation but may jeopardise the subject or may require intervention to prevent one of the other outcomes listed in the definition above, should also be considered serious.

N.B. Progressive disease and death due to disease are not considered SAEs but should be reported on the relevant forms.

Suspected Unexpected Serious Adverse Reaction (SUSAR): any suspected adverse reactions related to an IMP that are both unexpected and serious.

Causality

Most adverse events and adverse drug reactions that occur in this trial, whether they are serious or not, will be expected treatment related toxicities due to the drugs used in this trial. The assignment of causality should be made by the Investigator responsible for the care of the patient, using the definitions in the table below.

If any doubt about the causality exists the Investigator should inform the Trials Centre who will notify the Chief Investigators. The pharma companies and/or other clinicians may be asked to advise in difficult cases. In the case of discrepant views on causality between the Investigator and others, the case will be discussed by all parties. In the event that no agreement is made, the MHRA will be informed of both points of view.

**Causality**	**Description**
Unrelated	There is no evidence of any causal relationship.
Unlikely	There is little evidence to suggest there is a causal relationship (e.g. the event did not occur within a reasonable time after administration of the trial medication). There is another reasonable explanation for the event (e.g. the patient's clinical condition, other concomitant treatment).
Possible	There is some evidence to suggest a causal relationship (e.g. because the event occurs within a reasonable time after administration of the trial medication). However, the influence of other factors may have contributed to the event (e.g. the patient's clinical condition, other concomitant treatments).
Probable	There is evidence to suggest a causal relationship and the influence of other factors is unlikely.
Definite	There is clear evidence to suggest a causal relationship and other possible contributing factors can be ruled out.

UK GROUP SPECIFIC APPENDIX
Reporting Guidelines for Serious Adverse Events/SUSARs

All adverse events/reactions should be reported. Depending on the nature of the event the reporting procedures below should be followed. Any questions concerning adverse event/reaction reporting should be directed to the trials centre.

Adverse Reactions
All toxicities whether expected or not, should be recorded in the toxicity section of the relevant case report form (Toxicity Form 6) and sent to the Trials Centre within one month of the form being due. The severity for each adverse event will be determined by using the Common Terminology Criteria for Adverse Events v3.0 (CTCAE) as a guideline, wherever possible. The criteria are available online at http://ctep.cancer.gov/forms/CTCAEv3.pdf. In cases where the CTCAE criteria do not apply, severity should be coded according to the following criteria:

1 = Mild
2 = Moderate
3 = Severe
4 = Life threatening
5 = Fatal

Serious Adverse Events (SAE)
All SAEs occurring during treatment and up to 30 days following the last administration of study treatment must be recorded on the Serious Adverse Event Form 9. An SAE form must be completed, signed, dated and faxed to the Trials Centre within 24 hours (1 working day) of the treating clinician becoming aware of the SAE. A copy will be forwarded to the IKW central data management office in the Netherlands by the Cancer Trials Centre. All sections on the SAE form must be completed.

Note: see PORTEC-3 Case Report Form Completion Guidelines in section 12 of the Investigator Site File for notes on completion of SAE form. Any relevant treatment forms and anonymised copies of relevant investigations should be included if possible.

Reporting of SUSARs to Regulatory Authorities
Under the terms of the Clinical Trial Agreement between LUMC and CR UK and UCL Cancer Trials Centre, LUMC will inform all participating Country Co-ordinating Centres (including the CR UK and UCL Cancer Trials Centre) within 6 days of any fatal/life threatening events and within 14 days for all other events to ensure that Country Co-ordinators can forward this information to the appropriate Regulatory Authorities within the timelines stipulated in 'The Medicines for Human Use (Clinical Trials) Regulations 2004' (SI 2004 No. 1031). - i.e. fatal and life-threatening events within 7 days of notification with a follow-up report within a further 8 days and non-threatening events within 15 days.

The CR UK and UCL Cancer Trials Centre will be required to forward all SUSAR reports received from LUMC within 24 hours to the MHRA, and UK only SUSARs to MREC to comply with the above timelines.

Principal investigators should inform their Local Research Ethics Committee and/or Research & Development Office of any SUSARs and/or SAEs as required.

Outcome of SAEs/SUSARs
All SAEs/SUSARs will be followed-up until resolution. Investigators will be asked to provide follow-up information if the event has not resolved at the time of the initial report.

Annual Safety Reports
LUMC will provide an annual report of all SAEs/SUSARs to Country Co-ordinating Groups. The CRUK and UCL Cancer Trials Centre will forward this report to the MHRA and MREC.

TO REPORT A PORTEC-3 SAE

An SAE form must be completed and faxed to the CR UK and UCL Cancer Trials Centre within 24 hours of the clinician becoming aware of the event.

Please fax to:

- CR UK and UCL Cancer Trials Centre (Fax: 020 7679 9871)

- On receipt of the SAE form, the Cancer Trials Centre will fax a copy to the IKW Trial Office at Leiden University Medical Centre (Fax: +31 71 526 6712)

Appendix K: ANZGOG Specific Appendix to PORTEC-3 Protocol

This appendix is to be read in conjunction with the current approved version of the PORTEC-3 protocol

ANZGOG Study Coordinator/Chair: Dr. Linda MILESHKIN
TROG Study Chair: Dr Pearly Khaw

Investigator authorisation procedure, randomisation and procedures for collecting data.

1. Trial organisation

This trial is an Intergroup Trial, jointly conducted by the Gynaecological Cancer Intergroup (GCIG), and including ANZGOG, following the protocol designed by the Steering Committee (main protocol).

- The IKW Trial Office, Leiden University Medical Centre (LUMC) is the coordinating data centre in this Intergroup trial and LUMC is responsible for the overall trial conduct (including protocol finalisation, trial activation, data management, statistical analysis and publication).
- ANZGOG will use the main protocol developed by the Steering Committee. The present Group Specific Appendix details the participation of all ANZGOG members and **therefore it supercedes entirely or partially the corresponding chapters in the protocol.**
- The ANZGOG Coordinating Centre is the collaborating Data Centre in this trial.
- All investigator members of the ANZGOG will contact the ANZGOG Coordinating Centre to randomise patients.
- The ANZGOG Coordinating Centre will follow a “standard mail-box” procedure for this trial:
- Only the IKW Trial Office, LUMC will code the data, perform consistency checks on data and modify them. Only the IKW Trial Office, LUMC will do the analysis.
- There will be no direct communication between investigator members of ANZGOG and the IKW Trial Office, LUMC.

2. Investigator authorisation procedure

All regulatory procedures must be completed in cooperation with the ANZGOG Coordinating Centre before investigators can be authorised to register patients in this trial. Investigators will be authorised to register or randomise patients in this trial only when they have returned to the ANZGOG Coordinating Centre:

- Copy of the authorisation of the local Health Research Ethics Committee (HREC)
- Copy of Clinical Trial Notification Form and Therapeutic Goods Administration acknowledgement letter
- Signed Investigator’s agreement to participate in the study
- Copy of the participant information sheet and consent form approved by their local HREC
- Signature log signed by all staff who will complete study documentation
- Copy of laboratory reference ranges
- Copy of laboratory accreditation
- Completed Hospital Registration Form, Radiotherapy Registration Form, and Chemotherapy Registration Form

Each time an institution has become authorised to enter patients in this trial, the ANZGOG Coordinating Centre will inform the IKW Trial Office, LUMC, and will send a copy of the necessary authorization forms. The IKW Trial Office, LUMC will provide the ANZGOG Coordinating Centre with the institution number for the new investigator immediately.

NOTE: Patient registration/randomisation from centres not authorised will not be accepted.

3. Patient randomisation procedure

Patient registration will only be accepted from authorised investigators (see "Authorisation procedure").
An exhaustive list of questions to be answered during the randomisation procedure is included in the Registration Checklist. The responsible investigator should complete the Registration Checklist and all the baseline forms before the patient is randomised. The Pathology Review form should be faxed to the ANZGOG Coordinating Centre along with the Randomisation Checklist. All investigator members of ANZGOG should send the randomisation checklist to the ANZGOG Coordinating Centre by fax;

Australian sites to fax 02 9562 5026
New Zealand sites to fax +800 0279 2999

Randomisations will be completed Monday to Friday, 8am to 5.30pm (AUS Eastern Time). When the randomisation form is received, the ANZGOG Coordinating Centre will randomise the patients via the Internet at the IKW Trial Office, LUMC. The ANZGOG Coordinating Centre will randomise patients as described in Section 10 of the main protocol. At the end of this procedure, the sequential identification number and the treatment will be allocated to the patient. The ANZGOG Coordinating Centre and the local investigator will receive this information by an automatic E-mail. This patient identification number must be recorded on all forms. All forms should be then sent to the ANZGOG Coordinating Centre, which will send it to the IKW Trial Office, LUMC.

4. Drug Supply

Upon randomisation of an Australian patient to the chemotherapy arm of the trial, the ANZGOG Coordinating Centre will send a drug order form to the central pharmacy (at Peter MacCallum Cancer Centre), along with a copy of the confirmation of randomisation, and a fax coversheet which includes the patients height, weight and BSA. The drug will be sent by World Couriers for overnight delivery in metropolitan areas but may take longer for regional centres.

5. Group specific sub-study

ANZGOG is participating in a sub-study which collects data on patient preferences for chemotherapy. The sub-study protocol is found in Group Specific Appendix L.

6. Procedures for collecting data

The data will be reported on the LUMC case report forms (CRFs). However, these forms will be adapted to include some ANZGOG specific administrative information in the header.
All the Investigators participating on behalf of ANZGOG will send all the forms to:

PORTEC-3 Trial Coordinator
NHMRC Clinical Trial Centre
Locked Bag 77
CAMPERDOWN NSW 1450
AUSTRALIA

The ANZGOG Coordinating Centre will follow a “mail-box” procedure for this trial.
This means that:

- Signed original CRFs will be collected by the ANZGOG Coordinating Centre and sent regularly to the IKW Trial Office, LUMC according to the form flow schedule (provided with the CRFs)
- Investigators will not be allowed to send CRFs directly to the IKW Trial Office, LUMC
- The ANZGOG Coordinating Centre will not modify the forms nor enter them into the computer
- The IKW Trial Office, LUMC will enter the data in the computer for quality control and analysis.

When necessary, queries will be transmitted to the ANZGOG Coordinating Centre, which will send them to the investigators. The ANZGOG Coordinating Centre will then send the reply of the investigators back to the IKW Trial Office, LUMC.

7. Chemotherapy administration and hypersensitivity management

Chemotherapy administration and hypersensitivity management should be 'as per local institutional standard protocol'

8. Reporting adverse events

8.1 Definitions

All ANZGOG investigators will use the same definitions as defined in the main protocol (Section 9.2)

8.2 Reporting procedure

All serious adverse events (SAE and SUSAR) related or not to the study treatment that occur during the study treatment and during the 30 days after the last study treatment administration should be documented on the SAE form and must be reported by fax within one working day of the initial observation of the event to the IKW Trial Office, LUMC and copied to ANZGOG Coordinating Centre.

Any late Serious Adverse Drug Reaction (SADR), occurring after this 30 day period, should be reported if considered possible, probably or definitely related to the protocol treatment.

IKW Trial Office Fax: +31 71 526 6712

ANZGOG Coordinating Centre Fax for Australian sites 02 9562 5026
ANZGOG Coordinating Centre Fax for New Zealand sites:+800 0279 2999

Any questions raised by the LUMC/ IKW Trial Office will be forwarded via the ANZGOG Coordinating Centre who will ensure that these queries will be forwarded to the appropriate investigator and that follow-up information is also forwarded to the LUMC Safety Desk within one working day of receipt.

The ANZGOG Coordinating Centre will receive Safety Reports from the IKW Trial Office, LUMC. The ANZGOG Coordinating Centre will distribute these Safety Reports to all participating investigators who will in turn inform their local HRECs as per local requirements.

9. Quality assurance

ANZGOG/CTC follows the “standard mailbox” system (see procedures for collecting data).
Therefore, data forms will not be entered in the database of the ANZGOG Coordinating Centre and the ANZGOG Coordinating Centre will not perform any consistency checks on the CRFs.

On-site quality control

The ANZGOG Trial Coordinator will review queries generated by the IKW Trial Office, LUMC. The results of this review will be used to determine aspects of the trial and data management that require clarification for the ANZGOG participating sites. The Trial Coordinator will provide this information to the participating investigators and data managers. No modifications will be made to the CRFs during these procedures. ANZGOG will receive electronic reports of:

- Timeliness of data submission
- Completeness of data submission
- Summary of queries generated.

This trial will be reviewed as part of ANZGOG’s site audit program. For any site participating in this study, a proportion of patients from this trial may be selected for source data verification, the regulatory files and pharmacy records will be reviewed. The investigator, by agreeing to participate in this protocol, agrees to cooperate fully with any quality assurance visit undertaken by third parties, including representatives of ANZGOG, national and/or foreign regulatory authorities as well as to allow direct access to documentation pertaining to the clinical trial (including CRFs, patient hospital charts, other source documents and other study files) to these individuals.

External histology review

External histology review will be carried out to confirm eligibility of every patient. This will be carried out by nominated pathologists in each participating state and in New Zealand. Histology review information from these nominated pathologists is required for the patient registration procedure.

Victoria: Dr Jan Pyman and Dr Sarah Swain
New Zealand: Dr Diane Kenwright
NSW: Dr Peter Russell and Dr Catherine Coumaris
Queensland: Dr Gael Phillips and Dr Diane Payton
Tasmania: Dr David Challis
Western Australia: Dr Colin Stewart
South Australia: Dr Thomas Dodd

Radiotherapy guidelines and quality control
In order to ensure compliance with protocol guidelines, PORTEC 3 has been submitted and duly activated as a collaborative trial with the Trans Tasman Radiation Oncology Group (TROG). As of November 2008, this trial has been activated by TROG, where it is known as “TROG 08.04”.

In accordance with TROG policy, a technical review will be conducted for this study. Remote technical audits will be conducted by an independent reviewer. All Radiation Oncologists participating in this study will be required to submit a case study for review, prior to trial participation. Further audit of patients on trial will also be undertaken. Guidelines associated with this review process are contained within the TROG documentation relevant to this study.

Copies of source documents will be used to verify the accuracy of the CRF data and to confirm compliance with the protocol treatment requirements.

A checklist of information required for each RT QA case will be provided by the TROG QA Office. Review material will be required within four weeks of treatment completion. Copies of source documents required for review will include:

- Treatment prescription (including total dose and number of fractions)
- Daily dose record (including dates of treatment delivery)
- Radiotherapy treatment planning (RTP) electronic data file exported in DICOM RT or RTOG format for SWAN review
- Documentation to verify the dates of portal images taken during radiotherapy

The TROG QA Office will coordinate the review in consultation with the TROG Study PI and report to the Trial Management Committee. Results will be reported to the TMC at least 6 monthly and to the TROG Scientific Committee biannually.

All queries relating to the radiotherapy guidelines and quality assurance procedures should be directed to the TROG Study Chair.

TROG Study Chair: Dr Pearly Khaw
Division of Radiation Oncology
Peter MacCallum Cancer Centre
Locked Bag 1
A’Beckett Street VIC 8006
Tel: +61 3 96561111
Fax: +61 3 96561424
Email: pearly.khaw@petermac.org

Other quality controls
The IKW Trial Office, LUMC will assess timeliness of data submission every 3 months. The ANZGOG Coordinating Centre will provide a report of due and overdue data to each participating centre.

10. Ethical considerations

The responsible investigator will ensure that this study is conducted in compliance with the National Statement on Ethical Conduct in Human Research 2007 or Health Research Council of New Zealand guidelines as applicable. The study will be conducted according to the ICH Harmonised Tripartite Guideline for Good Clinical Practice as adopted by the Australian Therapeutic Goods Administration and applicable Privacy Laws.

11. Administrative responsibilities

The Principal Investigator

The Principal Investigator of ANZGOG will be responsible for clarifying protocol-specific and associated medical questions, and for overseeing satisfactory trial conduct in Australia and New Zealand.

Principal Investigator:

Dr. Linda MILESHKIN
Peter MacCallum Cancer Centre
St Andrews Place
EAST MELBOURNE VIC 3002
Phone: +61 3 9656 1697
Fax: +61 3 9656 1408
E-mail: Linda.Mileshkin@petermac.org

The Data Center

The ANZGOG Coordinating Center is responsible for handling investigator authorisation procedures, for randomisation of patients and will act as a "mailbox" in this trial (see forms and procedures for data collection). All queries should be addressed to the ANZGOG Coordinating Center who will forward them to the appropriate person and then send responses to the IKW Trial Office, LUMC contact in charge of this trial.

The ANZGOG Co-coordinating Centre is responsible for all administrative procedures required for the trial following ANZGOG procedures and applicable regulatory requirements. The Coordinating Centre is also responsible, as an intermediate, to guarantee the fluent communication between the IKW Trial Office, LUMC and the Investigators participating on behalf of the ANZGOG.

Data Center

ANZGOG Coordinating Centre
NHMRC Clinical Trial Centre
Locked Bag 77
Camperdown NSW 1450
Australia
Phone: 02 9562 5000
Fax: 02 9562 5094
Email: portec3@ctc.usyd.edu.au

Key contacts:

ANZGOG Associate Program Manager
Julie Martyn
Tel: +61 2 9562 5092
Fax: +61 2 9562 5094
E-mail: Julie.martyn@ctc.usyd.edu.au

The Group

ANZGOG is responsible as a group to guarantee the general compliance of their members with procedures described in this appendix.

All questions concerning membership in ANZGOG should be addressed to the chairman and/or associate program manager of ANZGOG.

Chairman:
Prof Michael Quinn
Department of Obstetrics and Gynaecology
The Royal Women's Hospital
Locked Bag 300
Grattan St & Flemington Rd
Parkville VIC 3052, Australia
Phone: + 61 3 8345 2000
E-mail: maquinn@unimelb.edu.au

ANZGOG Associate Program Manager:
Julie Martyn
ANZGOG Coordinating Centre
NHMRC Clinical Trial Centre
Locked Bag 77
Camperdown NSW 1450, Australia
Phone: +61 2 9562 5092
Fax: +61 2 9562 5094
E-mail: Julie.martyn@ctc.usyd.edu.au

12. Trial sponsorship and/or funding

The University of Sydney is the legal entity representing ANZGOG, responsible for the conduct of the study in Australia and New Zealand.

13. Trial Insurance

PORTEC-3 is an investigator initiated collaborative group study for which there is no industry sponsor designated to provide indemnity for the trial. **Therefore each participating centre must provide its own indemnity for the study.** Mutual indemnity arrangements are as set out in the investigator agreement between the participating centres and University of Sydney. The University of Sydney holds no-fault patient compensation insurance for trial participants

All employees of University of Sydney and staff at the ANZGOG Coordinating Centre are covered by Professional Indemnity; however this does not extend to other personnel conducting the trial at the participating centres.

14. Translational Research

ANZGOG centres may participate in the translational research aspects of this study. The level of participation for each site will be determined by them according to their ability to meet the protocol requirements for collecting, processing and storing the tissue and plasma samples. Patient information sheets and consent forms will be modified by each site according to the site’s level of participation. These will be reviewed by the ANZGOG coordinating centre prior to submission to the local Ethics Committee.

All patient samples will be retained at the site and at a time to be determined by the LUMC, samples will be sent to a central laboratory for analysis. Sites will provide the ANZGOG Coordinating Centre with a sample inventory log.

No study supplies will be provided for this trial, and the costs of shipping the samples will be covered by the sites. However, separate funds to cover these expenses will be sought by future applications for translational research grants by LUMC in collaboration with the participating groups.

Appendix K: UNICANCER French Group Specific Appendix to PORTEC-3 Protocol

The following protocol adaptations should be followed by the French sites:

5. Patient selection

5.1 Inclusion Criteria

To be eligible for this trial, patients should be older than 18 years.

7. Treatment

7.3 Radiotherapy

7.3.3 Adjustment or rules for Radiotherapy interruption

In case of acute toxicities during concomitant chemoradiation or exclusive radiation some adjustment of the RT treatment should be done according to the usual standard RT practice and according to the decision by the clinicians:

<u>Interruption criteria of the EBRT:</u>

⇨ *in case of extra-haematological toxicities:*

- Diarrhea Grade 3 => interruption of the RT until recovery of diarrhea < grade3
- Diarrhea Grade 4 => stop RT
- Hemorrhagic Cystitis => interruption of the RT until the stop of bleeding
- Other grade 4 toxicities due to RT => stop RT

⇨ *in case of haematological toxicities:*

- PNN< $500/mm^3$ (grade 4) ≤ 7 days => interruption of the RT until recovery PNN≥$1000mm^3$
- PNN< 500/ mm^3 (grade 4) > 7 days => stop RT
- Febrile neutropenia => stop to the RT
- Platelets < 25000/ mm^3 (grade 4) ≤ 7 days => interruption of the RT until recovery Platelets ≥ 75000/ mm^3
- Platelets < 25000/ mm^3 (grade 4) > 7days => stop RT

***7*.- In case of late toxicities related to radiotherapy**

The usual RT practices will be followed.

You will find below some bibliographies about it.

1- *Abayomi J, Kirwan J, Hackett A: The prevalence of chronic radiation enteritis following radiotherapy for cervical or endometrial cancer and its impact on quality of life. Europ. Journ. of Onco. Nurs.13 (2009) 262-267*

2- *Johnson N, Cornes P: Survival and recurrent disease after postoperative radiotherapy for early endometrial cancer: systematic review and meta-analysis. RCOG 2007 BJOG 1313-1020*

3- *Andreyev J: Gastrointestinal complications of pelvic radiotherapy: are they of any importance?. Gut 2005;54;1051-1054*

4- *Jereczek-Fossa B A, Badzio A, Jassem J:Time without symptoms and toxicity (TWIST) analysis of adjuvant radiation therapy for endometrial cancer. Radiotherapy and Oncol. 72 (2004) 175-181*

7.1.3. External beam pelvic radiation

<u>Technique:</u>

- IMRT is not allowed for French sites:

So, 3D conformal RT should be used.

- TDM procedure for dosimetry with vessel opacification:
In the absence of contraindications (essentially renal insufficiency or allergy to iodine) radiotherapy scanner is done with injection of contrast product. The procedure follows the recommendations related to the contrast agents used.

In conventional treatment conformational, the calculation of dose distributions is performed on the injected scanner as it does not take into account heterogeneities in the calculation of dose distributions.

Reporting Guidelines for Serious Adverse Events/SUSARs

1- European Definitions :

Adverse event : any untoward medicinal occurrence in a patient or clinical trial subject administered a medicinal product and wich does not necessarily have a causal relationship with this treatment.

Adverse reaction : all untoward and unintended responses to an investigational medicinal product related to any dose administered.

Serious adverse event or serious adverse reaction : any untoward medicinal occurrence or effect that at any dose results in death, is lifethreatning, requires hospitalisation or prolongation of existing hospitalisation, results in persistent or significant disability or incapacity, or is a congenital anomaly or birth defect.

Unexpected adverse reaction (SUSAR) : an adverse reaction, the nature or severity of wich is not consistent with the applicable product information (e.g. iinvestigator's brochure for an unauthorised investigational product or summary of product characteristics for an authorised product.

2 – Notification of adverse events (Directive 2001/20/EC – article 16-1 and 16-4)

The investigator shall report all serious adverse events immediately to the sponsor.

The sponsor shall keep detailed records of all adverse events which are reported to him by the investigator or investigators. Theses records shall be submitted to the Member States in whose territory the clinical trial is being conducted, if they so request.

So participating sites will report all serious adverse events as usual to UNICANCER – PV Unit as soon as possible and within 24h of investigator knowledge of event, using the SAE Reporting Form.

For all notification :

All reports must be sent to the Pharmacovigilance Unit:

UNICANCER/BECT
To the attention of Carole Delavault
E-mail address: pv-bect@UNICANCER
Phone: +33 (0)1 44 23 04 16
Fax: +33 (0)1 44 23 55 70
